# Supplementary material for: Awareness of climate change's impacts and motivation to adapt are not enough to drive action: A look of Puerto Rican farmers after Hurricane Maria
Source: PLoS One. 2021 Jan 27;16(1):e0244512. doi: 10.1371/journal.pone.0244512 (PMC7840010; doi:10.1371/journal.pone.0244512)
Supplement: S1 Table — Factor loadings are shown, as well as the Cronbach’s alpha for statements altogether. (DOCX) [file pone.0244512.s001.docx]

**S1 Table: Exploratory factor analysis results for pilot study’s assessment of the psychological distance of climate change scale**. Factor loadings are shown, as well as the Cronbach’s alpha for statements altogether.

| Statement | Factor 1 | Factor 2 | alpha |
| --- | --- | --- | --- |
| The effects of climate change are being felt today. | 0.9373 | -0.1532 | 0.860 |
| Climate change presents more risks than benefits to agriculture in Puerto Rico. | 0.9751 | -0.1041 |  |
| Climate change presents more risks than benefits to agriculture globally. | 0.8668 | -0.2320 |  |
| Farmers like me are likely to be negatively affected by climate change. | 0.9812 | -0.0758 |  |
| People who are not farmers are likely to be negatively affected by climate change. | 0.9604 | -0.1384 |  |
| There is scientific uncertainty about the potential impacts of climate change on Puerto Rico. | 0.5661 | 0.0902 |  |
| There is scientific uncertainty about the causes of climate change. | 0.3784 | 0.7184 |  |
| I am uncertain that the occurrence of strong hurricanes in the Atlantic is related to climate change. | 0.4303 | 0.7682 |  |

Note: The eigenvalue for Factor 1 was 5.1148, and 1.2273 for Factor 2.
