# Supplementary material for: Awareness of climate change's impacts and motivation to adapt are not enough to drive action: A look of Puerto Rican farmers after Hurricane Maria
Source: PLoS One. 2021 Jan 27;16(1):e0244512. doi: 10.1371/journal.pone.0244512 (PMC7840010; doi:10.1371/journal.pone.0244512)
Supplement: S2 Table — Frequencies and percentage were calculated based upon the 397 farmers that answered this section of the survey. (DOCX) [file pone.0244512.s002.docx]

**S2 Table. Reported adoption of agricultural practices and management strategies after Hurricane Maria to prepare for future extreme weather events.** Frequencies and percentage were calculated based upon the 397 farmers that answered this section of the survey.

| Agricultural practice | Frequency (%) |
| --- | --- |
| Acquire insurance/improve the plan I have | 42 (10.6) |
| Acquire solar panels | 37 (9.3) |
| Apply more synthetic inputs (e.g. fertilizers, herbicides, pesticides) | 34 (8.4) |
| Collect rainwater for irrigation | 50 (12.6) |
| Crop rotation | 84 (21.2) |
| Decrease tillage | 52 (13.1) |
| Diversify crops | 78 (19.6) |
| Expand my agricultural land | 34 (8.6) |
| Forage conservation | 15 (3.8) |
| Improve irrigation system | 62 (15.6) |
| Increase tillage | 35 (8.8) |
| Integrated management of diseases | 97 (24.4) |
| Plant trees to reduce erosion | 55 (13.9) |
| Seek new agricultural markets | 56 (14.1) |
| Countouring (*Siembras al contorno*) | 68 (17.1) |
| Switch from an annual crop (for example, peppers) to a perennial crop (for example plantains) | 34 (8.6) |
| Switch from a perennial crop to an annual crop | 31 (7.8) |
| Use compost | 65 (16.4) |
| Use of mulch | 26 (6.5) |
| Use of native crops/species | 37 (9.3) |
