# Supplementary material for: Awareness of climate change's impacts and motivation to adapt are not enough to drive action: A look of Puerto Rican farmers after Hurricane Maria
Source: PLoS One. 2021 Jan 27;16(1):e0244512. doi: 10.1371/journal.pone.0244512 (PMC7840010; doi:10.1371/journal.pone.0244512)
Supplement: S3 Table — Standardized coefficients (β), bootstrap standard error (SE), and significance level (p) are included. (DOCX) [file pone.0244512.s003.docx]

**S3 Table: Structural equation model structural results for our hypothesized model**. Standardized coefficients (β) of main and control variables, bootstrap standard error (SE), and significance level are included.

| Relationship | *b* | SE | *p* = |
| --- | --- | --- | --- |
| Age 🡪 Reported experience | 0.109 | 0.057 | 0.054 |
| Farm size 🡪 Reported experience | 0.007 | 0.058 | 0.903 |
| Gender 🡪 Reported experience | 0.016 | 0.059 | 0.783 |
| Education 🡪 Reported experience | -0.099 | 0.066 | 0.131 |
| Household income 🡪 Reported experience | 0.070 | 0.057 | 0.217 |
| Bonafide 🡪 Reported experience | -0.110 | 0.059 | 0.062 |
| Age 🡪 Reported damages | -0.064 | 0.054 | 0.240 |
| Farm size 🡪 Reported damages | -0.009 | 0.063 | 0.892 |
| Gender 🡪 Reported damages | -0.006 | 0.056 | 0.920 |
| Education 🡪 Reported damages | -0.188 | 0.059 | 0.001 |
| Household income 🡪 Reported damages | -0.077 | 0.060 | 0.199 |
| Bonafide 🡪 Reported damages | 0.136 | 0.062 | 0.028 |
| Reported experience 🡪 Psychological distance of climate change | 0.029 | 0.058 | 0.618 |
| Reported damages 🡪 Psychological distance of climate change | -0.018 | 0.063 | 0.778 |
| Psychological distance of climate change 🡪 Perceived self-capacity | -0.138 | 0.060 | 0.022 |
| Psychological distance of climate change 🡪 Perceived vulnerability | -0.162 | 0.065 | 0.013 |
| Perceived self-capacity 🡪 Motivation to adapt | 0.584 | 0.066 | 0.000 |
| Psychological distance of climate change 🡪 Motivation to adapt | -0.021 | 0.044 | 0.627 |
| Perceived vulnerability 🡪 Motivation to adapt | 0.254 | 0.075 | 0.001 |
| Motivation to adapt 🡪 Actual adoption of agricultural practices and management strategies | -0.018 | 0.055 | 0.743 |
