# Supplementary material for: Awareness of climate change's impacts and motivation to adapt are not enough to drive action: A look of Puerto Rican farmers after Hurricane Maria
Source: PLoS One. 2021 Jan 27;16(1):e0244512. doi: 10.1371/journal.pone.0244512 (PMC7840010; doi:10.1371/journal.pone.0244512)
Supplement: S4 Table — Standardized coefficients (β), bootstrap standard error (SE), and significance level (p) are included. (DOCX) [file pone.0244512.s004.docx]

**S4 Table. Structural equation model structural results for indirect effects of our hypothesized** **model.** Standardized coefficients (β), bootstrap standard error (SE), and significance level (*p*) are included.

| Relationship | *b* | SE | *p* = |
| --- | --- | --- | --- |
| Age 🡪 Psychological distance of climate change | 0.000 | 0.000 | 0.547 |
| Farm size 🡪 Psychological distance of climate change | 0.000 | 0.000 | 0.869 |
| Gender 🡪 Psychological distance of climate change | 0.001 | 0.004 | 0.781 |
| Education 🡪 Psychological distance of climate change | 0.000 | 0.004 | 0.976 |
| Household income 🡪 Psychological distance of climate change | 0.002 | 0.003 | 0.588 |
| Bonafide 🡪 Psychological distance of climate change | -0.007 | 0.013 | 0.569 |
| Reported experience 🡪 Perceived self-capacity | -0.003 | 0.063 | 0.613 |
| Reported damages 🡪 Perceived self-capacity | 0.004 | 0.013 | 0.773 |
| Age 🡪 Perceived self-capacity | -0.000 | 0.000 | 0.530 |
| Farm size 🡪 Perceived self-capacity | -0.000 | 0.000 | 0.867 |
| Gender 🡪 Perceived self-capacity | -0.000 | 0.001 | 0.783 |
| Education 🡪 Perceived self-capacity | -0.000 | 0.001 | 0.975 |
| Household income 🡪 Perceived self-capacity | -0.000 | 0.001 | 0.571 |
| Bonafide 🡪 Perceived self-capacity | 0.002 | 0.003 | 0.548 |
| Reported experience 🡪 Perceived vulnerability | -0.003 | 0.005 | 0.625 |
| Reported damages 🡪 Perceived vulnerability | 0.003 | 0.012 | 0.777 |
| Age 🡪 Perceived vulnerability | -0.000 | 0.000 | 0.552 |
| Farm size 🡪 Perceived vulnerability | -0.000 | 0.000 | 0.868 |
| Gender 🡪 Perceived vulnerability | -0.000 | 0.001 | 0.782 |
| Education 🡪 Perceived vulnerability | -0.000 | 0.001 | 0.976 |
| Household income 🡪 Perceived vulnerability | -0.000 | 0.001 | 0.590 |
| Bonafide 🡪 Perceived vulnerability | 0.002 | 0.003 | 0.571 |
| Reported experience 🡪 Motivation to adapt | -0.003 | 0.005 | 0.621 |
| Reported damages 🡪 Motivation to adapt | 0.003 | 0.012 | 0.773 |
| Age 🡪 Motivation to adapt | -0.000 | 0.000 | 0.540 |
| Farm size 🡪 Motivation to adapt | -0.000 | 0.000 | 0.868 |
| Gender 🡪 Motivation to adapt | -0.000 | 0.001 | 0.782 |
| Education 🡪 Motivation to adapt | -0.000 | 0.001 | 0.975 |
| Household income 🡪 Motivation to adapt | -0.000 | 0.001 | 0.576 |
| Bonafide 🡪 Motivation to adapt | 0.002 | 0.003 | 0.555 |
| Psychological distance of climate change 🡪 Motivation to adapt | -0.176 | 0.072 | 0.015 |
| Reported experience 🡪 Actual adoption of agricultural practices and management strategies | 0.000 | 0.001 | 0.758 |
| Reported damages 🡪 Actual adoption of agricultural practices and management strategies | -0.000 | 0.001 | 0.831 |
| Psychological distances of climate change 🡪 Actual adoption of agricultural practices and management strategies | 0.014 | 0.044 | 0.729 |
| Perceived capacity 🡪 Actual adoption of agricultural practices and management strategies | -0.034 | 0.107 | 0.728 |
| Perceived vulnerability 🡪 Actual adoption of agricultural practices and management strategies | -0.019 | 0.059 | 0.722 |
| Age 🡪 Actual adoption of agricultural practices and management strategies | 0.000 | 0.000 | 0.752 |
| Gender 🡪 Actual adoption of agricultural practices and management strategies | 0.000 | 0.000 | 0.827 |
| Education 🡪 Actual adoption of agricultural practices and management strategies | 0.000 | 0.000 | 0.976 |
| Household income 🡪 Actual adoption of agricultural practices and management strategies | 0.000 | 0.000 | 0.761 |
| Bonafide 🡪 Actual adoption of agricultural practices and management strategies | -0.000 | 0.000 | 0.761 |

Note: The structural pathway between farm size and actual adoption of agricultural practices was constrained by the model. Thus, it is not shown in the table.
