# Supplementary material for: Awareness of climate change's impacts and motivation to adapt are not enough to drive action: A look of Puerto Rican farmers after Hurricane Maria
Source: PLoS One. 2021 Jan 27;16(1):e0244512. doi: 10.1371/journal.pone.0244512 (PMC7840010; doi:10.1371/journal.pone.0244512)
Supplement: Attachment — Submitted filename: PONE-D-20-19200_reviewer.pdf [file pone.0244512.s006.pdf]

PLOS ONE

Dear PLOS ONE editor(s),
The full title of our article is "Puerto Rican farmers' psychological awareness of climate change, and adaptation perceptions after Hurricane Maria".
Best,
Luis Alexis Rodríguez-Cruz

--Manuscript Draft--

**Manuscript Number:**	PONE-D-20-19200
**Article Type:**	Research Article
**Full Title:**	Dear PLOS ONE editor(s),<br>The full title of our article is "Puerto Rican farmers' psychological awareness of climate change, and adaptation perceptions after Hurricane Maria".<br>Best,<br>Luis Alexis Rodríguez-Cruz
**Short Title:**	Psychological distance of climate change after a natural hazard
**Corresponding Author:**	Meredith T Niles<br>University of Vermont<br>UNITED STATES
**Keywords:**	psychological distance of climate change; structural equation model; natural hazards; small island developing states; adaptive capacity
**Abstract:**	Strengthening farmers' adaptive capacity is important for decreasing their vulnerability to natural hazards in this changing climate, and for safeguarding local food systems. One key step in strengthening adaptive capacity is understanding the relationship between farmers' experience with natural hazards and their perceptions of climate change, and its role in farmers' decision making around climate change adaptation. Here we explore Puerto Rican farmers' psychological distance of climate change after experiencing category 4 Hurricane Maria, in order to intersect the roles of experience, perceptions, and motivation for farmers' decision-making around climate change adaptation. Farmers throughout Puerto Rico were surveyed by Extension Services' agents (n = 405, 87% response rate) in 2018, eight months after Hurricane Maria. A structural equation model was used to evaluate how reported experience with past events and direct damages by Hurricane Maria related to farmers' psychological distance of climate change, and the association of these variables to farmers' motivation to adapt to climate change. We found that farmers psychological distance of climate change is both near and far, since they show a broad awareness to climate change's impacts both locally and globally in different dimensions (temporal, geographical, and social). Reported experience and direct damages by the hurricane were not linked to their psychological distance of climate change, and these did not relate to their motivation to adapt. These results suggest that using extreme events as a driver for climate belief and action may no longer be relevant, especially in a context where there is a high level of climate change belief and continued threat of extreme events. Thus, broadening analysis beyond individual perceptions as drivers of climate adaptation to how structural dynamics are linked to adaptive capacity could provide better understanding. This study is among the first to study the role of climate change experience and perceptions on farmers' climate change adaption after an extreme weather event in an island-archipelago setting.
**Order of Authors:**	Luis Alexis Rodríguez-Cruz, M.S.
	Meredith T. Niles
**Additional Information:**	
**Question**	**Response**
**Financial Disclosure**<br><br>Enter a financial disclosure statement that	MTN allocated funding from this study, which came from the College of Agriculture and Life Science and the Food Systems Graduate Program of the University of Vermont. Include this sentence at the end of your statement: The funders had no role in study

Powered by Editorial Manager® and ProduXion Manager® from Aries Systems Corporation

describes the sources of funding for the work included in this submission. Review the submission guidelines for detailed requirements. View published research articles from *PLOS ONE* for specific examples.<br><br>This statement is required for submission and **will appear in the published article** if the submission is accepted. Please make sure it is accurate.<br><br>**Unfunded studies**<br>Enter: *The author(s) received no specific funding for this work.*<br><br>**Funded studies**<br>Enter a statement with the following details:<br>• Initials of the authors who received each award<br>• Grant numbers awarded to each author<br>• The full name of each funder<br>• URL of each funder website<br>• Did the sponsors or funders play any role in the study design, data collection and analysis, decision to publish, or preparation of the manuscript?<br>• **NO** - Include this sentence at the end of your statement: *The funders had no role in study design, data collection and analysis, decision to publish, or preparation of the manuscript.*<br>• **YES** - Specify the role(s) played.<br><br>* typeset	design, data collection and analysis, decision to publish, or preparation of the manuscript.
**Competing Interests**<br><br>Use the instructions below to enter a competing interest statement for this submission. On behalf of all authors, disclose any competing interests that could be perceived to bias this work—acknowledging all financial support and any other relevant financial or non-financial competing interests.<br><br>This statement **will appear in the published article** if the submission is accepted. Please make sure it is accurate. View published research articles from *PLOS ONE* for specific examples.	I have read the journal's policy and the authors of this manuscript have the following competing interests: MTN is a member of the board of directors of The Public Library of Science (PLOS). This role has in no way influenced the outcome or development of this work or the peer review process, nor does it alter our adherence to PLOS ONE policies on sharing data and materials.

Powered by Editorial Manager® and ProduXion Manager® from Aries Systems Corporation

**NO authors have competing interests**<br><br>Enter: *The authors have declared that no competing interests exist.*<br><br>**Authors with competing interests**<br><br>Enter competing interest details beginning with this statement:<br><br>*I have read the journal's policy and the authors of this manuscript have the following competing interests: [insert competing interests here]*<br><br>* typeset	
**Ethics Statement**<br><br>Enter an ethics statement for this submission. This statement is required if the study involved:<br><br>• Human participants<br>• Human specimens or tissue<br>• Vertebrate animals or cephalopods<br>• Vertebrate embryos or tissues<br>• Field research<br><br>Write "N/A" if the submission does not require an ethics statement.<br><br>General guidance is provided below. Consult the submission guidelines for detailed instructions. **Make sure that all information entered here is included in the Methods section of the manuscript.**	The Committees on Human Subjects Serving the University of Vermont and the UVM Medical Center at the Research Protections Office approved our study on 04-Dec-2017. The study (CHRBSS: 18-0258) received Exemption Category 2. The consent obtained from the participants was oral. The consent was part of the survey booklet.

Powered by Editorial Manager® and ProduXion Manager® from Aries Systems Corporation

**Format for specific study types**<br><br>**Human Subject Research (involving human participants and/or tissue)**<br>• Give the name of the institutional review board or ethics committee that approved the study<br>• Include the approval number and/or a statement indicating approval of this research<br>• Indicate the form of consent obtained (written/oral) or the reason that consent was not obtained (e.g. the data were analyzed anonymously)<br><br>**Animal Research (involving vertebrate animals, embryos or tissues)**<br>• Provide the name of the Institutional Animal Care and Use Committee (IACUC) or other relevant ethics board that reviewed the study protocol, and indicate whether they approved this research or granted a formal waiver of ethical approval<br>• Include an approval number if one was obtained<br>• If the study involved *non-human primates*, add *additional details* about animal welfare and steps taken to ameliorate suffering<br>• If anesthesia, euthanasia, or any kind of animal sacrifice is part of the study, include briefly which substances and/or methods were applied<br><br>**Field Research**<br><br>Include the following details if this study involves the collection of plant, animal, or other materials from a natural setting:<br>• Field permit number<br>• Name of the institution or relevant body that granted permission	
**Data Availability**<br><br>Authors are required to make all data underlying the findings described fully available, without restriction, and from the time of publication. PLOS allows rare exceptions to address legal and ethical concerns. See the PLOS Data Policy and FAQ for detailed information.	No - some restrictions will apply

Powered by Editorial Manager® and ProduXion Manager® from Aries Systems Corporation

A Data Availability Statement describing where the data can be found is required at submission. Your answers to this question constitute the Data Availability Statement and **will be published in the article**, if accepted.<br><br>**Important:** Stating 'data available on request from the author' is not sufficient. If your data are only available upon request, select 'No' for the first question and explain your exceptional situation in the text box.<br><br>Do the authors confirm that all data underlying the findings described in their manuscript are fully available without restriction?	
**Describe where the data may be found in full sentences. If you are copying our sample text, replace any instances of XXX with the appropriate details.**<br><br>• If the data are **held or will be held in a public repository**, include URLs, accession numbers or DOIs. If this information will only be available after acceptance, indicate this by ticking the box below. For example: *All XXX files are available from the XXX database (accession number(s) XXX, XXX.).*<br>• If the data are all contained **within the manuscript and/or Supporting Information files**, enter the following: *All relevant data are within the manuscript and its Supporting Information files.*<br>• If neither of these applies but you are able to provide **details of access elsewhere**, with or without limitations, please do so. For example:<br><br>*Data cannot be shared publicly because of [XXX]. Data are available from the XXX Institutional Data Access / Ethics Committee (contact via XXX) for researchers who meet the criteria for access to confidential data.*<br><br>*The data underlying the results presented in the study are available from (include the name of the third party*	Data cannot be shared publicly because only a portion of the data was used in the reported study. We will release the de-identified data that we have used in the manuscript to a data repository upon acceptance of the article.

and contact information or URL).<br>• This text is appropriate if the data are owned by a third party and authors do not have permission to share the data.<br><br>* typeset	
Additional data availability information:	

Manuscript_Puerto Rican farmers' psychological awareness of climate change and adaptation perceptions after Hurricane Maria
Click here to download Manuscript Manuscript_June2020.docx

Puerto Rican farmers' psychological awareness of climate change, and adaptation perceptions after Hurricane Maria

Luis Alexis Rodríguez-Cruz[1] & Meredith T. Niles[2*]

[1]Food Systems Graduate Program, University of Vermont, Burlington, Vermont

[2]Department of Nutrition and Food Sciences and Food Systems Graduate Program, University of Vermont, Burlington, Vermont

*Corresponding author

E-mail: mtniles@uvm.edu (MTN)

LARC contributions: conceptualized research, survey development, project liaison, data entry, wrote manuscript draft.

MTN contributions: Conceptualized research, survey development, funding acquisition, edited manuscript draft.

Abstract

Strengthening farmers' adaptive capacity is important for decreasing their vulnerability to natural hazards in this changing climate, and for safeguarding local food systems. One key step in strengthening adaptive capacity is understanding the relationship between farmers' experience with natural hazards and their perceptions of climate change, and its role in farmers' decision making around climate change adaptation. Here we explore Puerto Rican farmers' psychological distance of climate change after experiencing category 4 Hurricane Maria, in order to intersect the roles of experience, perceptions, and motivation for farmers' decision-making around climate change adaptation. Farmers throughout Puerto Rico were surveyed by Extension Services' agents (n = 405, 87% response rate) in 2018, eight months after Hurricane Maria. A structural equation model was used to evaluate how reported experience with past events and direct damages by Hurricane Maria related to farmers' psychological distance of climate change, and the association of these variables to farmers' motivation to adapt to climate change. We found that farmers psychological distance of climate change is both near and far, since they show a broad awareness to climate change's impacts both locally and globally in different dimensions (temporal, geographical, and social). Reported experience and direct damages by the hurricane were not linked to their psychological distance of climate change, and these did not relate to their motivation to adapt. These results suggest that using extreme events as a driver for climate belief and action may no longer be relevant, especially in a context where there is a high level of climate change belief and continued threat of extreme events. Thus, broadening analysis beyond individual perceptions as drivers of climate adaptation to how structural dynamics are linked to adaptive capacity could provide better understanding. This study is among the first to study the

role of climate change experience and perceptions on farmers' climate change adaption after an extreme weather event in an island-archipelago setting.

Introduction

Farmers across the globe are at the forefront of climate change-related impacts, and are thus important actors in safeguarding our food systems [1,2] . Natural hazards, such as hurricanes and droughts, have made farming more difficult due to an increased frequency, and, in many cases, negatively affected farmers' livelihoods [3,4]. This is notably true of those who farm in the tropics, especially farmers in island states and territories, who are more vulnerable to the impacts of natural hazards given islands' unique characteristics, such as small territories and economies, dependence on imports, and disproportionate exposure to sea-level rise and extreme weather events [5–8].

Exposure, sensitivity, and adaptive capacity are three main determinants of vulnerability to natural hazards [9,10]. Farmers in small island states and territories, most of which are located in the tropics, farm in regions that are often disproportionately exposed and sensitive to natural hazards [10–12]. Exposure refers to islands being situated in regions prone to extreme weather events (e.g. Atlantic's 'Hurricane Belt'), and sensitivity relates to the degree to which such hazards change physical systems (e.g. farms) [9,12–14]. As such, strengthening adaptive capacity —defined as the set of abilities and resources individuals and populations have to anticipate, withstand, cope with, and recover from a hazard, and the potential or abilities they have to modify the system in order to be more resistant to impacts [11,15,16]—is key in decreasing vulnerability to natural hazards. This paper utilizes the psychological distance of

climate change as its framework to explore Puerto Rican farmers' experience with Hurricane Maria, and their perceptions of climate change adaptation to understand potential drivers and barriers for adopting behaviors that strengthen adaptive capacity. This framework, which has been primarily used in non-island settings, allow us to intersect experience, perceptions, and their association to adaptation [17–19]. Focusing on Puerto Rico fills a current gap on food systems research in small island states and territories focused on exposure and sensitivity to natural hazards. Furthermore, it gives us the ability to generate novel information for scholarship focused on Puerto Rico as well.

Adaptive capacity is complex, and involves many diverse and interrelated determinants [11,12]. In the case of farmers, the adoption of new adaptation technologies and practices, as well as the access to institutional resources and networks of support, are important for strengthening adaptive capacity [3,4,20–22]. Research in the Caribbean and Central America—areas exposed to Atlantic hurricanes—have shown that farm and farmers' characteristics, as well as their access to diverse markets and sources of support are important in reducing vulnerability to natural hazards [3,23–26]. Nevertheless, given the heterogeneity of islands' sociopolitical systems, context-specific research is important to better understand adaptive capacity [12,27]. This is evident in the Caribbean, given the region's neocolonial dynamics, where many islands' sovereignty resides on continental countries [28–30]. This is the case of Puerto Rico as an Unincorporated Territory of the United States. Studying Puerto Rican farmers' climate change adaptation could reflect broader sociopolitical structures' roles in the process of strengthening adaptive capacity.

Understanding the role of perceptions and beliefs as determinants of adaptive capacity is key to determining effective strategies [11,31]. As such, it is important to understand to what extent

beliefs and perceptions play a role in farmers' decision-making processes around climate change adaptation. Climate change adaptation occurs at different scales [20,32], and it is contextualized, highlighting that understanding the degree to which cognitive beliefs impact individuals' decision-making and willingness to act is important in assessing adaptive capacity [13,17,31,33]. While existing research demonstrates a link between climate perceptions and beliefs, and climate change adaptation amongst the general public [34], and farmers [17], there are varied results [33,35,36]. Importantly, most research focused on perceptions about climate and the role of cognitive beliefs in climate adaptation is from high-income countries [33]. There is limited evidence of this topic from small island developing states and territories, whose unique exposure and sensitivity to climate change and extreme events may influence how residents perceive climate change and their adaptive capacity.

To contribute to ~~fill~~ the current gaps in the literature, we examine survey results from 405 Puerto Rican farmers following Hurricane Maria, a category four hurricane, which impacted Puerto Rico in September 2017. We examine farmers' perceptions and beliefs about climate change, its potential links to their experience with the hurricane, as well as the extent to which these perceptions and beliefs influence their motivation to adapt to climate change using the theoretical framework of the psychological distance of climate change—simply put, how near or far people perceive climate change to be from themselves in various contexts [19,34,37,38]. This work can expand our understanding of the role of cognitive beliefs and perceived psychological distance for prompting adaptation behaviors amongst individuals beyond high-income and continental countries.

Theoretical framework

Construal Level Theory states that humans imagine the past and future through abstract constructs, since we are embedded in the here and now [37,38]. Psychological distance, a component of the aforementioned theory, is a subjective experience that uses the self as a reference point [37,38]. Psychological distance is measured in four components: temporal, social, geographical, and uncertainty of the distance or issue. The more distant, we perceive an issue or event to be across these dimensions, the more we rely on abstract constructs to make sense of it. In contrast, things we perceive as closer are more concrete. For example, as an event gets closer in time (e.g. conference presentation), the awareness of the dynamics involved become more concrete (e.g. transportation to the venue, nervousness, time management, and other variables). Thus, Construal Level Theory explains that we use mental constructs to build understanding of what transcends the here and now.

Applied in the realm of climate change communication and adaptation research, the psychological distance of climate change [19] refers to how near or far people (e.g. farmers) perceive climate change to be from themselves across the four dimensions: temporal (when climate change will or is occurring), geographical (where it happens), social (to whom it happens), and the hypothetical or uncertainty dimension (certainty of whether it occurs or not). Theoretically, this posits that individuals who are psychologically close to climate change are prone to perceive higher risks to hazards, and a greater motivation to act to adapt or mitigate climate change. Nonetheless, this assumption has not been consistent in research [35,36].

Research on the psychological distance of climate change

The aforementioned hypothesis has been the basis for many existing studies on the psychological distance of climate change (e.g. understanding how psychological distance affects the propensity for individuals to take action to mitigate or adapt to climate change). The current body of research focused on the psychological distance of climate change is both experimental [39,40], and observational [34,41). More specifically, scholarship has explored (1) the interrelatedness of the different dimensions, (2) the role of experiencing extreme weather events on individuals' psychological distance of climate change, and (3) the degree to which psychological distance of climate change affects behavior. Overall, evidence indicates inconsistent outcomes related to the psychological distance of climate change and its capacity to prompt action, as well as the understanding of how experiences with extreme events affects psychological distance, and the relatedness of the four dimensions varies [36]. These results may suggest that context matters; a natural hazard (e.g. hurricane), for example, can be experienced and perceived differently depending on context (e.g. sociodemographic characteristics, beliefs, adaptive capacity) [9,10]. Some studies support that the four dimensions of the psychological distance of climate change are interrelated. Moreover, studies suggest that being psychologically close impacts behavior. For example, Spence and colleagues (2011, 2012) demonstrated that experience with floods was linked to individuals' perceptions of climate change in the United Kingdom. Their studies showed that the four dimensions of the psychological distance of climate change were interrelated, and that reduced psychological distance related to promptness to climate change mitigation actions [19,34]. Similarly, but based in the United States, Singh et al. (2017) found

that the four dimensions were interrelated, and that those psychologically close expressed more support towards climate change adaptation policies. In that study, the temporal and uncertainty dimensions, by their own, did not have significant effect on policy support; however, those that expressed higher uncertainty over climate change and were socially distant, expressed less support towards policies [41]. Furthermore, research has shown that the dimensions, though often interrelated, depending on context, manifest differently [36]. Focused on New Zealand's farmers, Niles et al. (2015) applied the psychological distance of climate change framework in combination with Liebig's Law of the Minimum to assess adaptation behaviors, finding that decreasing psychological distance may prompt action from farmers. Farmers who perceived climate change impacts as geographically close, were more likely to feel more concern for climate change and motivation to adapt [17].

Research has also described how experience with extreme weather events relates to being psychologically close [17,34,36,41]. Asharibasam and Anuga (2018) found that farmers who had experienced extreme events were socially and geographically close, and that psychological distance mediated emotional regulation among farmers—the mental processes through which individuals decide how emotions are expressed and experienced [42]. Nevertheless, a recent meta-analysis performed by van Valkengoed and colleagues (2019) found that experience had small-to-moderate effects on climate change adaptation. Moreover, Larcom (2019) found that experience with a heatwave amongst the general population did not elicit adaptation behaviors in the United Kingdom, but did elevate concern [43]. Similarly, Albright and Crow (2019) found that direct flooding experience was not a predictor of concern for climate change amongst Colorado, United States, residents [44]. These more recent studies suggest that there may be a threshold of individual exposure to natural hazards and their potential to catalyze behavioral

change; indeed, as the rate of extreme events increases, it could be probable that such events may saturate in people's experiences and become "more normal".

The aforementioned studies also highlight the importance of context, but also bring attention to how psychological distance of climate change research is limited in its generalizability [35]. Furthermore, most research on the matter have used data on reported experience, and not on direct experience (e.g. material impacts such as damage). Zanocco et al. (2018) performed case studies of residents whose communities were directly impacted by extreme events to understand the relationship between experience of extreme event and views of climate change. Applying Construal Level Theory perspectives of psychological distance, they found that geographical proximity to the event did not align with climate change views, but reported harm did [45]. This quote from Demski et al. (2017), as cited by Zanocco et al. (2018), underscores the aforementioned: "Self-reported measures of experience that include: direct physical experience and material impacts of an event, well-defined in terms of personal effects and damage, are less susceptible to biased reporting, providing the best proxy available for objective experience" (p. 152) [45] .

This paper attempts to fill several gaps in the research by assessing the psychological distance of climate change amongst Puerto Rican farmers after experiencing Hurricane Maria. First, we will assess Puerto Rican farmers' psychological distance of climate change after experiencing an extreme weather event. To the best of our understanding, there is no study that looks at the relationship between actual experience with extreme events and its relationship with the psychological distance of climate change in small island developing states and territories. Second, we will assess the extent to which ~~the experience with Maria (~~both reported experience with other extreme events, and direct damages caused by Hurricane Maria~~)~~ relate to

psychological distance of climate change. As stated above, most research has looked on reported experience. Third, in order to increase our understanding on the degree to which cognitive beliefs play a role in climate change adaptation and therefore relate to individual adaptive capacity, we examine the relationship of psychological distance of climate change to farmers' adaptation perceptions.

The present study

This study focuses in Puerto Rico, an unincorporated territory of the United States that imports around 85% of the food it consumes, and that is going through a social-economic crisis because of its billion dollar debt and political situation [46–49]. In response to Puerto Rico's debt crisis in 2016-, the United States' government created a Fiscal Management and Oversight Board that imposed austerity measures, and made visible Puerto Rico's lack of political agency as a territory of the United States [28,29]. Simultaneous and previous to this change, the agricultural sector in Puerto Rico was experiencing positive changes, such as a rise in new farmers, production increases, and increased awareness of food security [23,49–51]. Such agricultural gains were important since Puerto Rico, like the broader Caribbean, was experiencing a steady decline in farms since the 1990's due to trade liberalization, globalization, and other external forces [5,24,51,52]. Nevertheless, such gains were erased when both Hurricane Irma and Hurricane Maria, two of the strongest in the Atlantic's history, hit Puerto Rico in September 2017.
The local Department of Agriculture (2018) reported that both hurricanes caused $2 billion in total losses, with the majority of losses from Maria ($228 million in production losses, and $1.8

billion in agricultural infrastructure losses). Such damages significantly impacted Puerto Rican agriculture, which, according to the USDA, is comprised of many small-scale farmers with an average farm size of 59 acres (approximately 23 hectares) [53], who focus mostly on domestic markets, and who have an average annual income of less than $20,000 [52]. Focusing this study on Puerto Rican farmers after experiencing a major storm, not only allow us to advance our understanding of the psychological distance of climate change, but provides us the opportunity to produce important applied knowledge around strengthening adaptive capacity to safeguard local agriculture and food access [17,18].

Objective, research questions, and hypotheses

Our study focuses on understanding how the psychological distance of climate change manifests in an island-archipelago setting, amongst a vulnerable population (farmers). Specifically, we intend to assess (1) the psychological distance of climate change amongst farmers in Puerto Rico, an unincorporated territory of the United States and Caribbean non-UN member Small Island Developing State after an extreme-weather event, (2) the extent to which psychological distance of climate change is related to experience and to (3) other cognitive beliefs such as motivation to adapt, perceived capacity (self-efficacy or perceived instrumentality), and perceived vulnerability. We ask: 1) How do farm and farmer characteristics, and direct experiences with extreme events i.e. reported damages) relate to the psychological distance of climate change? And, 2) in light of experiencing an extreme event, what is the role of psychological distance of climate change in motivating Puerto Rican farmers to adapt to climate

change? Following research on the psychological distance of climate change, we hypothesize that farmers' psychological distance of climate change will be driven by their experience (e.g. reported damages) with Hurricane Maria (H1). Furthermore, we predict that psychological distance of climate change will be related to perceived capacity, perceived vulnerability, and motivation to adapt (H2). Fig 1 shows our hypothesized model.

Fig 1. Hypothesized model. Farm and farmer characteristics' variables included as controls.

Methodology

Data gathering

Previous studies of on-farm management and climate change perceptions, and on the psychological distance of climate change [17,22,41,54] informed the development of a survey instrument, which was contextualized to Puerto Rico and the general objectives of the main study. This study was carried out in collaboration with the Extension Service of the University of Puerto Rico at Mayagüez. Thus, feedback from Extension partners were also included in the survey's development. The survey was translated into Spanish, and piloted in February 2018 with a pool of Puerto Rican farmers (n = 31), and was shared amongst Extension agricultural agents—enumerators of the survey.

Surveys (n = 440) were randomly administered to farmers, according to the five regions the Extension Service divides Puerto Rico: Arecibo, Caguas, Mayagüez, Ponce, and San Juan. Survey deployment was based on Extension Services' recommendations to access a wide range of farmers, and because agricultural agents had an established presence. Surveys were administered between May and July 2018 in person. A total of 405 surveys were completed, resulting in an 87% response rate based on the American Association for Public Opinion calculator.

Assessing the psychological distance of climate change.

Eight questions were used to assess the four dimensions of the psychological distance of climate change (temporal, social, geographical, and uncertainty) (Table 1). The temporal dimension was assessed with one item, while the social and geographical dimensions were assessed with two each. Questions assessing the social and geographical dimensions were intended to separately assess local and distant concerns. Uncertainty was assessed through three statements. Variables were reversed where needed, in order to analyze ascending levels of the psychological distance of climate change. All items were included as a scale variable for the psychological distance of climate change—Cronbach's alpha = 0.74, which suggests good internal validity (55). "Strongly agree" and "agree" answers for dimensions' statements were combined as "psychologically far" for ease of interpretation. The same was done for "psychologically close" with opposite answers. "Neutral" was kept separate.

Structural equation model

A structural equation model was built to explore the extent to which psychological distance of climate change relates to farmers' experience with Hurricane Maria, and their motivation to adapt to climate change. Structural equation models allow quantitative analysis of the direct, indirect, and mediated interactions between variables, and the incorporation of latent constructs [56–58]. Fig 1 shows the hypothesized model. Two variables assessed experience: "reported past experience" and "reported hurricane damages". Variables for perceived capacity and vulnerability were included. Those variables, alongside psychological distance of climate change, were included to grasp an understanding of the role of cognitive beliefs in the context of adaptive capacity. The structural equation model was deployed in Stata 15.1, using Maximum Likelihood with bootstrapping (1000 iterations) to control for Type I errors and increase statistical power [57,59]. The following Goodness-of-fit criteria were used: Chi-square (X2), Standardized Root Mean Square Residual (SRMR), Comparative fit index (CFI), Root mean square error of approximation (RMSEA) [58].

Table 1: Survey variables used in this study.

Category	Variable	Question/Statement	Measure
Climate change perceptions	Global climate	The global climate is changing.	5-point Likert scale—from strongly disagree to strongly agree
	Global temperature	Average global temperatures are increasing.	5-point Likert scale
	Anthropogenic causes	Human activities such as fossil fuel combustions are an important cause of climate change.	5-point Likert scale
Psychological Distance of Climate Change			
Temporal Dimension	Today	The effects of climate change are already being felt today.	5-point Likert scale
Social dimension	Farmers	Farmers like me are not likely to be affected negatively by climate change.	5-point Likert scale

Category	Variable	Question/Statement	Measure
	General public	People who aren’t farmers are likely to be affected negatively by climate change.	5-point Likert scale
Geographical dimension	Local agriculture	Climate change does not presents more risk than benefits to agriculture in Puerto Rico.	5-point Likert scale
	Global agriculture	Climate change presents more risks than benefits to agriculture globally.	5-point Likert scale
Uncertainty dimension	Impact uncertainty	There is scientific uncertainty about the potential impacts of climate change on agriculture.	5-point Likert scale
	Cause uncertainty	There is scientific uncertainty about the causes of climate change.	5-point Likert scale
	Hurricane uncertainty	I am uncertain that the occurrence of strong hurricanes in the Atlantic is related to climate change.	5-point Likert scale

Category	Variable	Question/Statement	Measure
Cognitive beliefs	Motivation to adapt	I feel motivated to change my agricultural practices to prepare for future extreme weather events like Hurricane Maria.	5-point Likert scale
	Perceived self-capacity	I feel that I have the capacity to change my agricultural practices to prepare for future potential extreme weather events like Hurricane Maria.	5-point Likert scale
	Perceived vulnerability	I believe my farm is vulnerable to future extreme weather events like Hurricane Maria.	5-point Likert scale
Experience with extreme weather events	Reported experience	I have faced similar extreme weather events like Hurricane Maria in the past ten years.	5-point Likert scale
	Reported damages	How would you describe the damages, if any, caused by Hurricane Maria to your farm?	5-point Likert—from no damages to total loss
Farmer and farm characteristics*	Age	In which year you were born?	Continuous

Category	Variable	Question/Statement	Measure
	Bonafide[a]	Do you participate in the bonafide program of the Puerto Rico Department of Agriculture?	Binary—yes or no
	Education level	What is the highest level of education you have completed?	Ordinal—from elementary school to PhD degree
	Farm size	How many *cuerdas*[1] of terrain do you manage in your farm?	Continuous
	Gender	What is your gender?	Binary—female or male
	Household income	What is your approximate household income, including all far and off-farm income?	Ordinal—from Less than $20,000 to more than $90,000

[a]The bonafide[a] program of the Puerto Rico Department of Agriculture provides farmers with agricultural incentives, and benefits then with various tax exemptions.

Results

Participants and the impact of Hurricane María

Farmers had an average age of 54, and farmed an average of 58 *cuerdas* (approximately 56 acres). The great majority of respondents had attended college (67%) (Table 2). Table 3 contrasts farmers' demographics and farm characteristics with those reported in Puerto Rico's 2017 Agricultural Census [53]. Respondents generally were similar to average census statistics, with the exception of having higher rates of formal higher education and somewhat higher percentage of income from farming. It is important to note that the recent census is not comparable to the previous one done in 2012 [53]. Moreover the 2017 census, performed in 2018, had a lower response rate, and USDA reported some difficulty in performing the census given post Hurricane Maria conditions [53,60]. Farmer respondents were distributed throughout Puerto Rico with 15% in the Arecibo region, 22% in the Caguas region, 19% in the Mayagüez region, 25% in the Ponce region, and 22% in the San Juan region. (Percentages do not sum 100% because some farms extend beyond one municipality that border with another). The majority of farmers experienced damages from Hurricane María; 42.6% reported total loss of their farms, while 45.6% reported significant damages (Fig 2). The majority of farmers (55.1%) disagreed and strongly disagreed (hereafter disagreed) that they had faced similar events to Hurricane Maria.

Table 2: Mean statistics of control variables.

Variable	Scale	Frequency (%)	Mean ± SD	n
Age	Continuous	-	54.0 ± 13.5	391
Bonafide	Yes	210 (52.8)	-	398
	No	188 (47.2)		
Education level	Elementary School	21 (5.2)	-	401
	Junior High School	13 (3.2)		
	Some High School	15 (3.7)		
	High School Diploma	82 (20.5)		
	Some College	42 (10.5)		
	Technical Degree	25 (6.2)		
	Associate Degree	41 (10.2)		
	Bachelor's Degree	109 (27.2)		
	Master's Degree	46 (11.5)		
	PhD	7 (1.8)		
Farm size	Continuous	-	58.1 ± 98.5	383
Gender	Female	55 (14.0)	-	395
	Male	340 (86.0)		
Household income	Less than $20,000	138 (36.4)	-	379
	$20,000 - $40,999	125 (33.0)		
	$41,000 - $60,999	52 (13.7)		
	$61,000 - $80,999	36 (9.5)		

Variable	Scale	Frequency (%)	Mean ± SD	n
	More than $90,000	28 (7.4)		

Table 3: Demographic variables of our study's surveyed farmers in comparison to USDA 2017 Agricultural Census data for Puerto Rico.

Category	Present study	USDA 2017 Census data
Average farm size	58.1 *cuerdas* (majority farmed > 20 *cuerdas*)	59.3 *cuerdas* (majority farmed > 20 *cuerdas*)
Years farming	Majority > 10 years	Majority > 10 years
Average age	54	61
Gender	Great majority is male	Great majority is male
Education	Majority reported High School Diploma or more	Majority reported High School Diploma or less
Income from farming	> 50%	< 50%
Household income	Majority reported < $20,000	Majority reported < $20,000

Note: Data from the census is for farms' principal operators. In Puerto Rico, this does not necessarily means that they are the sole owner of the farm. Neither the census or the local Department of Agriculture detail the number of Puerto Rican farmers who participate in the bonafide program; however, in a media report in 2019 (61), according to the Secretary of Agriculture, Carlos Flores, among Puerto Rico's 17,000 farmers, 4,000 are part of the bonafide program; 24% in comparison to the 53% in our study. It is also important to note that the Secretary's comment contrast with the number of farmers provided by the USDA census, which was conducted in 2018, and had a low response rate (53).

Fig 2: Farmer's past experience with events similar to Hurricane Maria (top) and reported hurricane damage (bottom).

Fig 3 shows results for farmers' perceptions around climate change and adaptation. An overwhelming majority of Puerto Rican farmers believe that the global climate is changing (86% strongly agree and agree; hereafter agreed), and that it has anthropogenic causes (91.4%). As well, 84.4% of Puerto Rican farmers agreed that they feel motivated to change their agricultural practices to better prepare for future extreme events. Though farmers perceive their farms to be vulnerable to future extreme weather events (93.5%), they understand themselves capable to adapt to climate change (79.0%).

Fig 3: Climate change perceptions and behavioral motivations among Puerto Rican farmers.

Puerto Rican farmers' psychological distance of climate change

We found that Puerto Rican farmers understand climate change as both a local and global phenomenon, being both psychologically close and psychologically distant across various measures. While we find evidence across all four dimensions of psychological distance for perceived "closeness", we also find that farmers demonstrate awareness for climate change effects beyond Puerto Rico in all dimensions (psychologically far) (Fig 4).

Fig 4: Results for variables of the psychological distance of climate change. To ease understanding, answers were collapsed: “agree” and “strongly agreed” are shown as “psychologically far”, and “disagree” and “strongly disagree” are shown as “psychologically close”.

Respondents demonstrated a temporally close relationship to climate change with the majority disagreeing (95.1%) that the effects of climate change are not being felt today. Regarding the geographical dimension, Puerto Rican farmers perceive that climate change will ~~both~~ negatively impact agriculture at both local and global scales (Fig 4). The majority disagreed (89.4%) that climate change does not presents more risk than benefits to agriculture in Puerto Rico (e.g. Puerto Rico’s agriculture is at risk). Farmers also agreed (90.0%) with the statement focused on the global scale, “Climate change presents more risks than benefits to agriculture globally”. At the social dimension, farmers expressed that climate change will both affect farmers (people like them), and the general public (people not like them). The majority (92%) agreed that farmers like them will likely be affected negatively by climate change (psychologically close); 89% agreed that people who aren’t farmers will also likely to be affected negatively by climate change (psychologically far).

We found the greatest variability in the uncertainty dimensions of psychological distance of climate change. Thirty nine percent (39%) of respondents agreed that there is scientific uncertainty about the potential impacts of climate change on agriculture, 14% were neutral, while 47% disagreed with the statement. The majority (60%) disagreed that there is scientific

uncertainty about the causes of climate change; 16% were neutral, and 24% agreed with the statement. Regarding the statement related to feeling uncertain about the relationship between climate change and occurrence of strong hurricanes in the Atlantic, 25% agreed, 13% were neutral, and 63% disagreed. Furthermore, respondents overwhelmingly agreed that human activities are an important cause of climate change 3.2% disagreed, 5.4% were neutral, and 91.4% agreed).

Structural equation model

The structural equation model (S1 table, S2 table, S3 table, and fig 5) demonstrates that four of the seven hypothesized pathways were statistically significant ($p < 0.05$); S1 table shows results for all variables, including control variables. Goodness-of-fit criteria suggests the model is acceptable: RMSEA = 0.059, CFI = 0.861, SRMR = 0.058. Furthermore, the model showed that for motivation to adapt, our main dependent variable, $R^2 = 0.410$.

Figure 5: Structural equation model results for the hypothesized model. Thick arrows show significance ($p < 0.05$). Standardize coefficients are also shown (*b*). The model controls for farmer and farm characteristics, including age, gender, household income, farm size, education, and bonafide. Goodness-of-fit criteria suggest an overall good fit: RMSEA = 0.059, CFI = 0.861, SRMR = 0.058. R^2 for motivation to adapt was 0.410. Results of indirect effects and total effects are in S2 table and S3 table, respectively.

The model shows that direct experience (reported hurricane damages) and reported past experience with extreme events are not significantly predictive of the psychological distance of

climate change amongst Puerto Rican farmers, a rejection of H1. Moreover, none of the control variables (age, gender, farm size, bonafide, education, and income) were linked to the psychological distance of climate change (S1 table). However, both perceived capacity and vulnerability are significantly negatively related to the psychological distance of climate change ($p < 0.05$) (H2). In other words, farmers that perceived climate change as more distant were more likely to report lower rates of perceived capacity ($b = -0.140$, $p = 0.017$), and were less likely to report higher rates of perceiving their farms as vulnerable to future extreme weather events ($b = -0.164$, $p = 0.011$). We find no significant direct relationship between psychological distance of climate change and motivation to adapt; however, both perceived capacity ($b = 0.578$, $p = 0.000$) and perceived vulnerability ($b = 0.256$, $p = 0.000$) were positively linked to motivation to adapt. We find no significant relationships between control variables and past experience with extreme events (S1 Table). Regarding reported hurricane damage, farmers who are participants of the bonafide program of the Puerto Rico Department of Agriculture were more likely ($b = 0.134$, $p = 0.034$) to report higher rates of damages to their farms due to Hurricane Maria. Furthermore, education level was negatively linked to reported damages by Hurricane Maria ($b = -0.177$, $p = 0.002$), meaning that, increasing levels of formal education were linked to reporting lower rates of damages.

Discussion

This study examined Puerto Rican farmer's psychological distance of climate change, and its relationship to reported damages following Hurricane Maria and experience with past extreme

weather events, in order to better understand potential drivers and barriers to strengthen adaptive capacity. We expand the current literature to utilize psychological distance of climate change beyond continental and high-income countries, and to understand how farmers' psychological distance of climate change relates to other cognitive beliefs in a vulnerable island-archipelago setting. We found that Puerto Rican farmers recognize climate change as a local and global issue (fig 4). Hence, they are neither psychologically close nor far, but psychologically aware of climate change's impacts across multiple time frames, geographies, and social constructs. We also found that neither reported experience with extreme weather events, nor the direct damages effected by Maria were linked to farmers' psychological distance of climate change. Furthermore, farmers overwhelmingly perceived themselves self-capable and motivated to adapt to climate change. Nevertheless, motivation was not directly linked to their psychological distance of climate change.

Studies have used psychological distance to climate change to better understand whether this framing can prompt individual mitigation or adaptation actions. Moreover, the trend in the literature is that reduced psychological distance—mainly through experience with extreme weather events—will correlate with action [17–19,35,36,39,41]. Our results provide a different picture—we found that Puerto Rican farmers cannot be characterized as psychologically close or psychologically distant to climate change, but are psychologically aware of the impacts related to climate change at different scales. Furthermore, we showed that direct experience with extreme weather events may not be the principal link to reduce psychological distance to climate change (Fig 5). As well, psychological distance did not have a direct effect on motivation to adapt, but it did have an indirect effect on it through perceived self-capacity and vulnerability (S2 table and S3 table).

These results show that Puerto Rican farmers recognize climate change as being both near and far, which indicates that in the Caribbean context, where climate change is recognized as happening [62], a psychological distance framing may not be relevant. Our results on overall climate change perceptions, and farmers psychological distance of climate change, align with data the Extension Service has on northeastern Puerto Rican farmers climate change opinions, and with a study by the Puerto Rican Department of Natural Resources that state that Puerto Ricans believe in, are aware of, and are concerned about climate change [63]. Puerto Rican farmers, overwhelming, understand that the climate is changing. This awareness of climate change as a threat is strong in Latin America and the Caribbean [62], where farmers and non-farmers are aware of the impacts related to climate change [64–66]. Surveyed farmers understand that climate change is happening now (temporal), that it will affect farmers and non-farmers (social), and that it will affect local and global agriculture (geographical). This could suggest that when a threshold for climate change belief is passed, individuals recognize it as a problem that goes beyond their own self-interest. Furthermore, it could be that there is an understanding of the interactions (e.g. feedback loops) and interconnectedness amongst scales in and beyond a place, and indicates that there may be a saturation point for the psychological distance of climate change framing. In other words, when enough people recognize the problem, both locally and globally, it may longer catalyze action. This should be further studied.

Our respondents were heavily affected by Hurricane María; 42.6% reported total loss, while 45.6% reported significant losses. Given the absolute prevalence of damage across our population, these outcomes may relate to the fact that we find no relationship of reported damage to psychological distance. As such, these results suggest that there may also be a threshold for level of damage and frequency of extreme events that may then fail to impact psychological

distance. When a group of people consistently face extreme events, especially ones that are wholly devastating, they could no longer think about such events as being close and driving climate change perceptions.

Our structural equation model showed that farmers who are bonafide participants in the Puerto Rico Department of Agriculture (52.8%) were more likely to report higher rates of damages (R^2 = .050). To participate in that program, 50% or more of farmers' income must come from agriculture. Farmers who are bonafide are exempted from some state and municipal taxes, and have access to other agricultural incentives. In our study, those that were bonafide had an average farm size of 85 *cuerdas*, and 76% of their income came from agriculture. This could point to the relationship of structural determinants' (e.g. policy structures) to climate change's effects on farmers. We did not have variables looking at type of agricultural incentives, or access to governmental, community-based resources or assets; future studies could look at how policy structures, coupled with federal laws and Puerto Rico's context as a US territory, impact farmers' adaptive capacity. Furthermore, another line of research could focus on the extent to whichh dependence on Puerto Rico Department of Agriculture incentives relate to on-farm management and farm systems. This is timely given that Puerto Rico's recent incentives code, Law 60 of 2019, changed various regulations that have been in place for over 30 years [67]. Furthermore, given that farmers overwhelmingly reported feeling self-capable and motivated to adapt—variables that have been shown positively linked to decision-making around climate change adaptation [33]—future research and practitioners could focus on actual adaptation of agricultural practices (beyond intentions and cognitive factors), and those drivers and barriers at broader levels (e.g. community, institutional, regional, national) to strengthen adaptive capacity.

The structural equation model (Fig 5) shows that perceiving climate change to be psychologically distant is linked to reporting lower rates of perceived self-capacity and perceived vulnerability to extreme weather events. This aligns with the psychological distance of climate change assumptions on how one's awareness relates to our promptness to act to mitigate or adapt to climate change. Nevertheless, farmers' psychological distance of climate change (and other variables in the model) does not explain much of the variability in both perceived self-capacity ($R^2 = 0.019$) and vulnerability ($R^2 = 0.026$). This further suggests that in areas constantly affected by extreme weather events, other variables beyond the ones explored here may be more relevant in explaining and fostering adaptation. Farmers in Latin America, the Caribbean, and Africa have expressed concern for climate change at different scales, but such perceptions are poorly linked to actual climate change adaptation [42,65,66,68]. Puerto Rican farmers overwhelmingly feel motivated to adapt to climate change, and that is positively linked to perceived self-capacity and perceived vulnerability. Nonetheless, taking into account the damage caused by Hurricane María and its aftermath, as well as the poor subsequent recovery efforts, our findings suggest that to strengthen farmers' adaptive capacity we must look at other determinants beyond the individual level.

In small island developing states and territories, where climate change impacts are already being felt to a greater extent than in high-income continental countries, cognitive beliefs nor psychological distance to climate change may be strong components of adaptive capacity. Farmers in Latin America [65,66], and the Caribbean [68,69] have been noted to lack institutional structures of support. In Puerto Rico, Perfecto and colleagues (2019) found that social capital and support networks were pivotal for Puerto Rican coffee farmers' recovery after Hurricane María, and that their agroecological practices, such as agroforestry, and other

management styles were not sufficient for farms to be resilient and resistant within the catastrophic context of Hurricane Maria [70]. Hurricane María affected all of Puerto Rico, causing 2,975 deaths [71], and decimated the archipelago's agriculture. As such, we argue that reasons and circumstances beyond the self (cognitive beliefs) deserve more attention to better understand Puerto Rican farmers' decision-making, and increase their adaptive capacity. Taken together, these results show that the psychological distance of climate change may not be an appropriate framework to understand cognitive beliefs' role in adaptive capacity, especially in an island setting, but potentially increasingly as climate change beliefs become saturated towards acknowledgement of the issue. We understand we are reaching a point where climate change-related conversations and perceptions are widely accepted. Future research could further generate understanding on that. Farmers in island and disadvantaged areas, who are at the forefront of constant climate change-related impacts, might have already gotten to a point where experiencing climate change is not the most important driver of concern nor motivation to adapt to climate change. Caribbean farmers face institutional barriers to better adapt to climate change [6,23,24,49,51], and are localized in a region that still today is subject to colonial relationships and power dynamics that increase their vulnerability to natural hazards [28–30]. It is important to understand the degree to which psychological awareness (not distance) of climate change relates to other cognitive beliefs and decision-making, but this cannot be done through ignoring the circumstances in which farmers are embedded. Climate change adaptation cannot be reduced to something that is dependent on the individual because then we could be contributing on the assertion that the genesis of vulnerability lies on the individual [72]. Vulnerability to natural hazards is a systemic issue, and farmers adaptation to climate change, as a decision-making process, is subject to structural interactions that cannot be overlooked.

Limitations

We note some limitations in this study. First, to the best of our knowledge, there is no previous study looking at Puerto Rican or Caribbean farmers' psychological distance of climate change before and after an extreme weather event. We lack information on the issue before Hurricane Maria or other events. This limits our ability to understand if their psychological awareness is driven by experience. Nonetheless, Extension Service's internal data before Hurricane Maria showed that farmers were aware of climate change's impacts at different scales. Second, farmers surveyed showed higher percentage of income from farming than that reported in census data [53], and we had an overrepresentation of bonafide farmers. Nevertheless, most of the demographic factors of surveyed farmers here align with those of the latest agricultural census for Puerto Rico (table 3). Furthermore, given that most farmers surveyed for this study receive information from, or are linked to the Extension Service, this might suggest that they have higher access to information and resources, which may affect their understanding of climate change and other related issues. As research in island systems on this topic continues to grow, there is opportunity for broader engagement with farmers, including those not directly integrated with Cooperative Extension.

Conclusion

Farmers' adaptive capacity must be strengthened to decrease their vulnerability to natural hazards. In order to do so, cognitive beliefs must be considered given that climate change

adaptation—a set of decisions at the individual level—is one key action to increase adaptive capacity. Here we assessed Puerto Rican farmers' climate change adaptation perceptions after Hurricane Maria to understand the role of experience with extreme events on the psychological distance of climate change, and its relation to motivation to adapt. We found that Puerto Rican farmers are psychologically aware of climate change impacts at different levels—local to global—and that such awareness is not driven by their experience with extreme events. These findings contrast with research in continental and high-income countries that seeks to reduce psychological distance to climate change amongst individuals to prompt mitigation and adaptation actions. Furthermore, we expand literature cemented on the assumption that being psychologically close enacts change, and that such psychological distance is driven by experience. We showed that neither reported experience with extreme events, nor farmers' actual experienced damages by Hurricane Maria were determinants of farmers psychological distance of climate change. Furthermore, here we demonstrate that context matters, and that the adaptive capacity of individuals embedded in vulnerable contexts—farmers in an island disproportionately exposed and sensitive to climate change's impacts—is not highly dependable on individual actions. We suggest that variables, such as institutional frameworks of support, must be better understood. Furthermore, research on the psychological distance of climate change should consider that we might be arriving to a threshold where climate change perceptions are not significant drivers of change. Hence, we suggest that understanding peoples' "psychological awareness of climate change" and its relation to context could provide a more nuance comprehension of climate change adaptation than focusing on how being "close" or "far" enacts change.

Acknowledgements

We acknowledge all the Puerto Rican farmers that participated in this project, and the Extension Service's agricultural agents who enumerated the surveys. We are grateful to Aníbal Ruiz-Lugo, assistant interim dean of the Extension Service, for leading the logistics and efforts on-site to carry out this project. Much thanks to Maritzabel Morales and the administrative team of the Extension Service for scanning all the surveys, and to Olivia Peña for assisting in data entry. We are also thankful to María del Carmen Rodríguez-Rodríguez, Robinson Rodríguez-Pérez, and Fernando Pérez-Muñoz for assisting in the completion of the project. We are grateful to Jim Vigoreaux, Kiria Hurtado, and Carmen González Toro for proofreading the Spanish version of the survey. We also acknowledge the colleagues who read the first draft, and provide us with meaningful review comments. Specially, we thank Amy Trubek, Teresa Mares, Maya Moore, Carolyn Hricko, and Caitlin B. Morgan. Much thanks as well to the University of Vermont's Food Systems Graduate Program and College of Agriculture and Life Sciences for providing funding in a timely manner to complete this research.

References

1. FAO. The state of food security and nutrition in the world: safeguarding against economic slowdowns and downturns. 2019.
2. Porter J, Xie L, Challinor A, Cochraen K, Howden M, Mohsin M, et al. Food Security and Food Production Systems. In: Climate Change 2014: Impacts, Adaptation, and

Vulnerability Part A: Global and Sectoral Aspects Contribution of Working Group II to the Fifth Assessment Report of the Intergovernmental Panel on Climate Change. Field, C.B., V.R. Barros, D.J. Dokken, K.J. Mach, M.D. Mastrandrea, T.E. Bilir, M. Chatterjee, K.L. Ebi, Y.O. Estrada, R.C. Genova, B. Girma, E.S. Kissel, A.N. Levy, S. MacCracken, P.R. Mastrandrea, and L.L. White. Cambridge, UK, and New York, NY: Cambridge University Press; 2014. p. 485–533.

3. Holt-Giménez E. Measuring farmers' agroecological resistance after Hurricane Mitch in Nicaragua: a case study in participatory, sustainable land management impact monitoring. Agriculture, Ecosystems & Environment. 2002 Dec;93(1–3):87–105.
4. Shinbrot XA, Jones KW, Rivera-Castañeda A, López-Báez W, Ojima DS. Smallholder Farmer Adoption of Climate-Related Adaptation Strategies: The Importance of Vulnerability Context, Livelihood Assets, and Climate Perceptions. Environmental Management. 2019 May;63(5):583–95.
5. FAO. Food security and nutrition in small island developing states (SIDS). Policy Paper. 2014;16.
6. Graham B. Profile of the Small-Scale Farming in the Caribbean. Food and Agriculture Organization; 2012 p. 62.
7. IPCC. The IPCC's Fifth Assessment Report: What's in it for Small Island Developing States? Climate & Development Knowledge Network. 2014;
8. Scobie M. Accountability in climate change governance and Caribbean SIDS. Environment, Development and Sustainability. 2018;20(2):769–87.
9. Adger WN. Vulnerability. Global Environmental Change. 2006 Aug;16(3):268–81.

10. Wisner B, Gaillard J, Kelman I. Framing disaster: Theories and stories seeking to understand hazards, vulnerability and risk. In: The Routledge Handbook of Hazards and Disaster Risk Reduction. 1st ed. London; New York: Routledge; 2012. p. 18–33.
11. López-Marrero T. An integrative approach to study and promote natural hazards adaptive capacity: a case study of two flood-prone communities in Puerto Rico: An integrative approach to study and promote natural hazards adaptive capacity. Geographical Journal. 2010 Feb 25;176(2):150–63.
12. López-Marrero T, Wisner B. Not in the Same Boat: Disasters and Differential Vulnerability in the Insular Caribbean. Caribbean Studies. 2012;40(2):129–68.
13. Adger WN, Vincent K. Uncertainty in adaptive capacity. Comptes Rendus Geoscience. 2005 Mar;337(4):399–410.
14. Smit B, Wandel J. Adaptation, adaptive capacity and vulnerability. Global Environmental Change. 2006 Aug;16(3):282–92.
15. Brooks N, Adger WN. Assessing and Enhancing Adaptive Capacity. In: Adaptation Policy Frameworks for Climate Change: Developing Strategies, Policies and Measures. Cambridge, UK: Cambridge University Press; 2005. p. 165–82.
16. Wisner B, Blaikie P, Cannon T, Davis I. At Risk: Natural hazards, people’s vulnerability and disasters. 2nd ed. New York and London: Routledge; 2004.
17. Niles MT, Lubell M, Brown M. How limiting factors drive agricultural adaptation to climate change. Agriculture, Ecosystems and Environment. 2015;200:178–85.
18. Ray A, Hughes L, Konisky DM, Kaylor C. Extreme weather exposure and support for climate change adaptation. Global Environmental Change. 2017;46:104–13.

19. Spence A, Poortinga W, Pidgeon N. The Psychological Distance of Climate Change. Risk Analysis. 2012;32(6):957–72.

20. Adger WN. Social Capital, Collective Action, and Adaptation to Climate Change. Economic Geography. 2003;79(4):387–404.

21. Fernandez M, Méndez VE. Subsistence under the canopy: Agrobiodiversity's contributions to food and nutrition security amongst coffee communities in Chiapas, Mexico. Agroecology and Sustainable Food Systems. 2019 May 28;43(5):579–601.

22. Niles MT, Mueller ND. Farmer perceptions of climate change: Associations with observed temperature and precipitation trends, irrigation, and climate beliefs. Global Environmental Change. 2016;39:133–42.

23. Álvarez-Berríos NL, Soto-Bayó S, Holupchinski E, Fain SJ, Gould WA. Correlating drought conservation practices and drought vulnerability in a tropical agricultural system. Renewable Agriculture and Food Systems. 2018 Jun;33(3):279–91.

24. Lowitt K, Hickey GM, Saint Ville A, Raeburn K, Thompson-Colón T, Laszlo S, et al. Factors affecting the innovation potential of smallholder farmers in the Caribbean Community. Regional Environmental Change. 2015 Oct;15(7):1367–77.

25. Rosset PM, Sosa BM, Jaime AMR, Lozano DRÁ. The Campesino-to-Campesino agroecology movement of ANAP in Cuba: Social process methodology in the construction of sustainable peasant agriculture and food sovereignty. Journal of Peasant Studies. 2011;38(1):161–91.

26. Saint Ville AS, Hickey GM, Phillip LE. Institutional analysis of food and agriculture policy in the Caribbean: The case of Saint Lucia. 2017 [cited 2018 Aug 15]; Available from: http://dx.doi.org/10.1016/j.jrurstud.2017.03.004

27. Scandurra G, Romano AA, Ronghi M, Carfora A. On the vulnerability of Small Island Developing States: A dynamic analysis. Ecological Indicators. 2017;84:382–92.

28. Borges-Méndez R, Caron C. Decolonizing Resilience: The Case of Reconstructing the Coffee Region of Puerto Rico After Hurricanes Irma and Maria. Journal of Extreme Events. 2019 Mar;06(01):1940001.

29. Félix G, Holt-Giménez E. Hurricane María: An Agroecological Turning Point for Puerto Rico? Food First Backgrounder. 2017;23(4).

30. Moulton AA, Machado MR. Bouncing Forward After Irma and Maria: Acknowledging Colonialism, Problematizing Resilience and Thinking Climate Justice. Journal of Extreme Events. 2019 Mar;06(01):1940003.

31. Grothmann T, Patt A. Adaptive capacity and human cognition: The process of individual adaptation to climate change. Global Environmental Change. 2005 Oct;15(3):199–213.

32. Ostrom E. Polycentric systems for coping with collective action and global environmental change. Global Environmental Change. 2010 Oct;20(4):550–7.

33. van Valkengoed AM, Steg L. Meta-analyses of factors motivating climate change adaptation behaviour. Nature Climate Change. 2019 Feb;9(2):158–63.

34. Spence A, Poortinga W, Butler C, Pidgeon NF. Perceptions of climate change and willingness to save energy related to flood experience. Nature Climate Change. 2011;1(1):46–9.

35. Brügger A. Understanding the psychological distance of climate change: The limitations of construal level theory and suggestions for alternative theoretical perspectives. Global Environmental Change. 2020 Jan;60:102023.

36. McDonald RI, Chai HY, Newell BR. Personal experience and the "psychological distance" of climate change: An integrative review. Journal of Environmental Psychology. 2015;44:109–18.
37. Trope Y, Liberman N. Construal-Level Theory of Psychological Distance. Psychological Review. 2010;117(2):440–63.
38. Liberman N, Trope Y. The Psychology of Transcending the Here and Now. Vol. 322, Source: Science, New Series. 2008.
39. Brügger A, Morton TA, Dessai S. "Proximising" climate change reconsidered: A construal level theory perspective. Journal of Environmental Psychology. 2016;46:125–42.
40. Jones C, Hine DW, Marks ADG. The Future is Now: Reducing Psychological Distance to Increase Public Engagement with Climate Change. Risk Analysis. 2017;37(2):331–41.
41. Singh AS, Zwickle A, Bruskotter JT, Wilson R. The perceived psychological distance of climate change impacts and its influence on support for adaptation policy. Environmental Science & Policy. 2017;73:93–9.
42. Acharibasam JW, Anuga SW. Psychological distance of climate change and mental health risks assessment of smallholder farmers in Northern Ghana: Is habituation a threat to climate change? Climate Risk Management. 2018;21:16–25.
43. Larcom S, She P-W, van Gevelt T. The UK summer heatwave of 2018 and public concern over energy security. Nature Climate Change. 2019 May;9(5):370–3.
44. Albright EA, Crow D. Beliefs about climate change in the aftermath of extreme flooding. Climatic Change. 2019 Jul;155(1):1–17.

45. Zanocco C, Boudet H, Nilson R, Satein H, Whitley H, Flora J. Place, proximity, and perceived harm: extreme weather events and views about climate change. Climatic Change. 2018;149(3–4):349–65.

46. Bueno R. Puerto Rico, Climatic Extremes, and the Economics of Resilience. Climate Policy Brief. 2017;(6).

47. Bueno R, Herzfeld C, Stanton E a., Ackerman F. The caribbean and climate change: The costs of inaction [Internet]. Stockholm Environment Institute and Global Development and Environment Institute, Tufts University; 2008 [cited 2018 Apr 12] p. 35. Available from: http://www.sei-us.org/climate-and-energy/Caribbean_Inaction_Cost.htm%5Cnhttp://www.gdae.org/CaribbeanClimate.html

48. Carro-Figueroa V. Agricultural Decline and Food Import Dependency in Puerto Rico: A Historical Perspective on the Outcomes of Postwar Farm and Food Policies. Caribbean Studies. 2002;30(2):77–107.

49. Gould WA. Caribbean Regional Climate Sub Hub Assessment of Climate Change Vulnerability and Adaptation and Mitigation Strategies. 2015;67.

50. Myrna Comas-Pagán. Plan de sguridad alimentaria. San Juan, Puerto Rico: Puerto Rico Department of Agriculture; 2014 p. 40.

51. Irizarry-Ruiz C. Desafíos de la agricultura en Puerto Rico para garantizar la seguridad alimentaria. In: Retos que conlleva la reutilización de aguas residuales en la agricultura. San Juan, Puerto Rico; 2016.

52. Weis T. Small farming and radical imaginations in the Caribbean today. Race & Class. 2007 Oct;49(2):112–7.

53. USDA NASS. 2017 Census of Agriculture - Puerto Rico Data [Internet]. 2020 [cited 2020 Jun 12] p. 117. Available from: https://www.nass.usda.gov/Publications/AgCensus/2017/index.php
54. Haden VR, Niles MT, Lubell M, Perlman J, Jackson LE. Global and Local Concerns: What Attitudes and Beliefs Motivate Farmers to Mitigate and Adapt to Climate Change? PLoS ONE [Internet]. 2012 [cited 2018 Sep 3];7(12). Available from: www.plosone.org
55. Nunnally JC. Psychometric Theory. McGraw-Hill; 1978.
56. Iacobucci D. Everything you always wanted to know about SEM (structural equations modeling) but were afraid to ask. Journal of Consumer Psychology. 2009 Oct;19(4):673–80.
57. Luu TA, Nguyen AT, Trinh QA, Pham VT, Le BB, Nguyen DT, et al. Farmers' Intention to Climate Change Adaptation in Agriculture in the Red River Delta Biosphere Reserve (Vietnam): A Combination of Structural Equation Modeling (SEM) and Protection Motivation Theory (PMT). Sustainability. 2019 May 27;11(10):2993.
58. Schreiber JB, Nora A, Stage FK, Barlow EA, King J. Reporting Structural Equation Modeling and Confirmatory Factor Analysis Results: A Review. The Journal of Educational Research. 2006 Jul;99(6):323–38.
59. Tang J-W, Chen M-L, Chiu T-H. An Exploratory Study on Local Brand Value Development for Outlying Island Agriculture: Local Food System and Actor-Network Theory Perspectives. Sustainability. 2018 Nov;10(11):4186.
60. USDA NASS. USDA Releases 2018 Puerto Rico Census of Agriculture [Internet]. 2020 [cited 2020 Jun 12]. Available from: https://www.nass.usda.gov/Newsroom/2020/06-09-2020.php

61. Díaz Rolón A. Aspira a que haya más agricultores bonafide [Internet]. El Vocero de Puerto Rico. 2019 [cited 2020 Jun 12]. Available from: https://www.elvocero.com/gobierno/aspira-a-que-haya-m-s-agricultores-bonafide/article_017eaa76-a2b8-11e9-9255-8773684782d5.html

62. Lee TM, Markowitz EM, Howe PD, Ko CY, Leiserowitz AA. Predictors of public climate change awareness and risk perception around the world. Nature Climate Change. 2015;5(11):1014–20.

63. Departamento de Recursos Naturales de Puerto Rico. Estudio de percepción pública sobre el riesgo y la resiliencia al cambio climático [Internet]. San Juan, Puerto Rico: Puerto Rico Department of Natural Resources; 2018 Sep [cited 2020 Jun 13] p. 95. Available from: http://drna.pr.gov/wp-content/uploads/2019/01/Informe-final-Estudio-de-percepcion-publica-sobre-cambio-climatico.pdf

64. Forero EL, Hernández YT, Zafra CA. Percepción latinoamericana de cambio climático: metodologías, herramientas y estrategias de adaptación en comunidades locales. Una revisión. Revista UDCA Actualidad & Divulgación Científica. 2014 Mar 10;17(1):73–85.

65. Viguera B, Alpizar F, Harvey CA, Martínez-Rodríguez MR, Saborío-Rodríguez M. Percepciones de cambio climático y respuestas adaptativas de caficultores costarricenses de pequeña escala. Agronomía Mesoamericana. 2019 May 1;333–51.

66. Viguera B, Alpizar F, Harvey CA, Martínez-Rodríguez MR, Saborío-Rodríguez M, Contreras L. Climate change perceptions and adaptive responses of small-scale farmers in two Guatemalan landscapes. Agronomía Mesoamericana. 2019;30(2):313'331.

67. Código de incentivos de Puerto Rico. P. de la C. 1635, 60 Jul 1, 2019.

68. Campbell, Donovan, Barker D, McGregor D. Dealing with drought: Small farmers and environmental hazards in southern St. Elizabeth, Jamaica. Applied Geography. 2011;31:146–58.

69. Stancioff C, Stojanov R, Kelman I, Němec D, Landa J, Tichy R, et al. Local Perceptions of Climate Change Impacts in St. Kitts (Caribbean Sea) and Malé, Maldives (Indian Ocean). Atmosphere. 2018 Nov 22;9(12):459.

70. Perfecto I, Hajian-Forooshani Z, Iverson A, Irizarry AD, Lugo-Perez J, Medina N, et al. Response of Coffee Farms to Hurricane Maria: Resistance and Resilience from an Extreme Climatic Event. Scientific Reports [Internet]. 2019 Dec [cited 2019 Nov 5];9(1). Available from: http://www.nature.com/articles/s41598-019-51416-1

71. Santos-Burgoa C. Acertainment of the Estimated Excess Mortality from Hurricane Maria in Puerto Rico.pdf [Internet]. 2018 [cited 2019 May 20]. Available from: https://publichealth.gwu.edu/sites/default/files/downloads/projects/PRstudy/Acertainment%20of%20the%20Estimated%20Excess%20Mortality%20from%20Hurricane%20Maria%20in%20Puerto%20Rico.pdf

72. Ribot J. Cause and response: vulnerability and climate in the Anthropocene. The Journal of Peasant Studies. 2014 Sep 3;41(5):667–705.

Supporting information

S1 Table. Structural equation model structural results for our hypothesized model. Standardized coefficients (β), bootstrap standard error (SE), and significance level (*p*) are included.

S2 Table. Structural equation model structural results for indirect effects of our hypothesized model. Standardized coefficients (β), bootstrap standard error (SE), and significance level (*p*) are included.

S3 Table. Structural equation model structural results for total effects of our hypothesized model. Standardized coefficients (β), bootstrap standard error (SE), and significance level (p) are included.

Fig 1_Hypothesized model

Click here to download Figure Fig1.tiff.tif

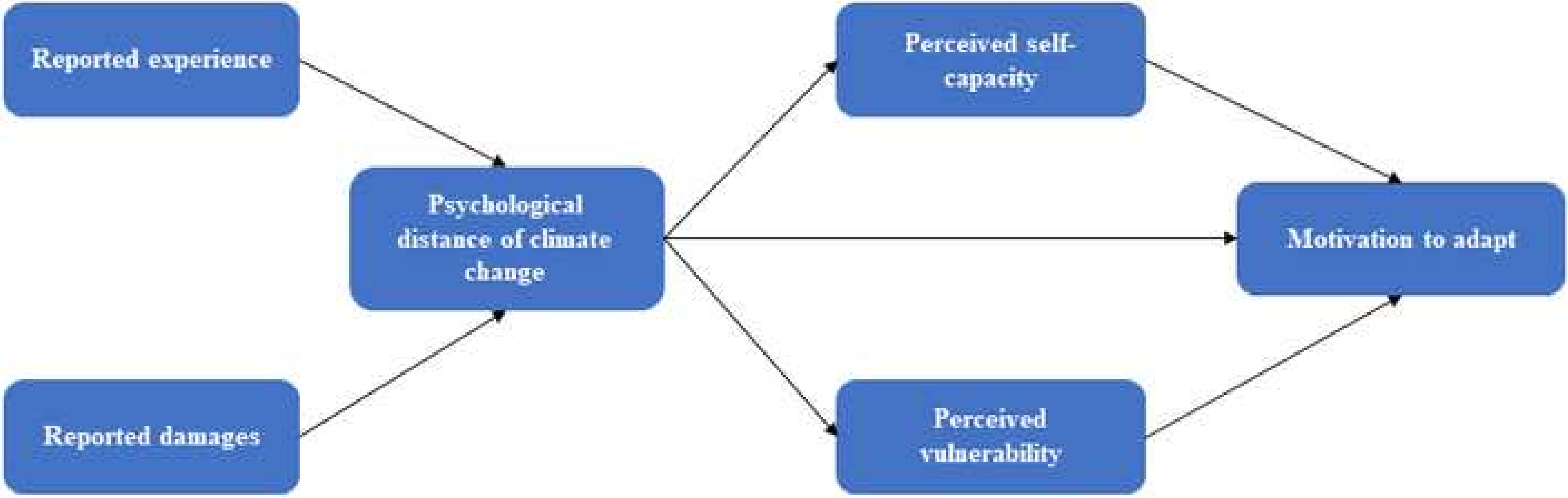

Fig 2_Experience with extreme events

Click here to download Figure Fig 2.tiff.tif

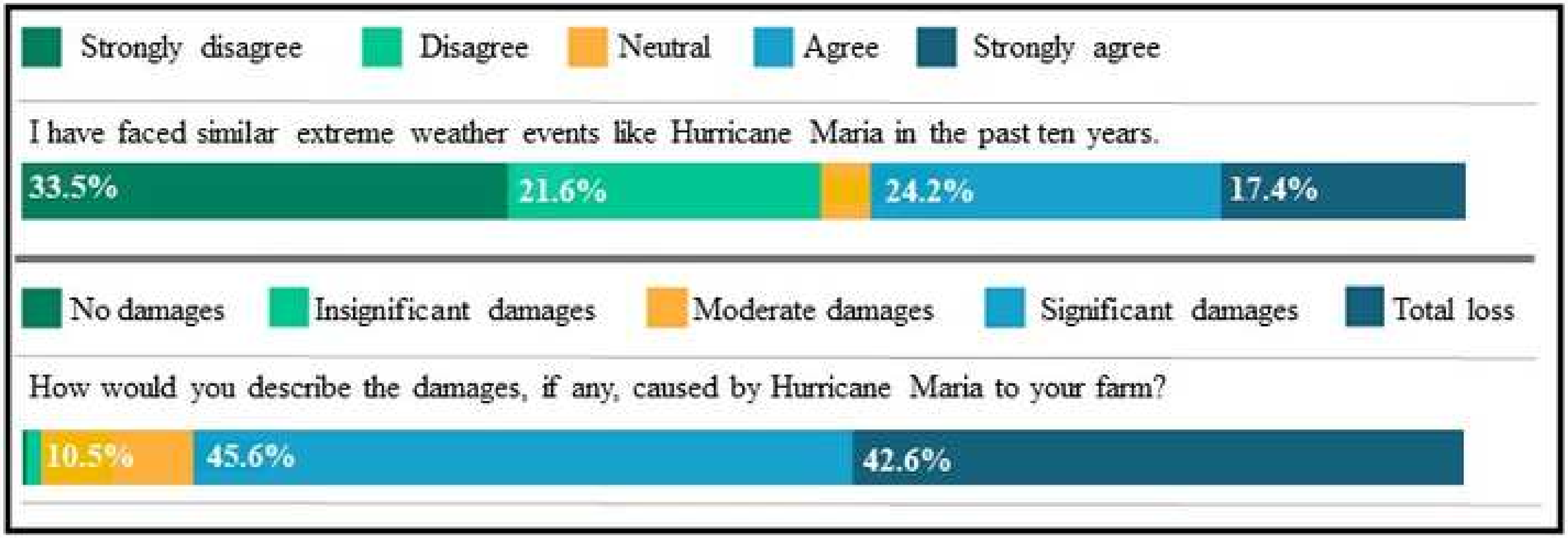

Fig 3_General climate change and adaptation perceptions

Click here to download Figure Fig 3.tiff.tif

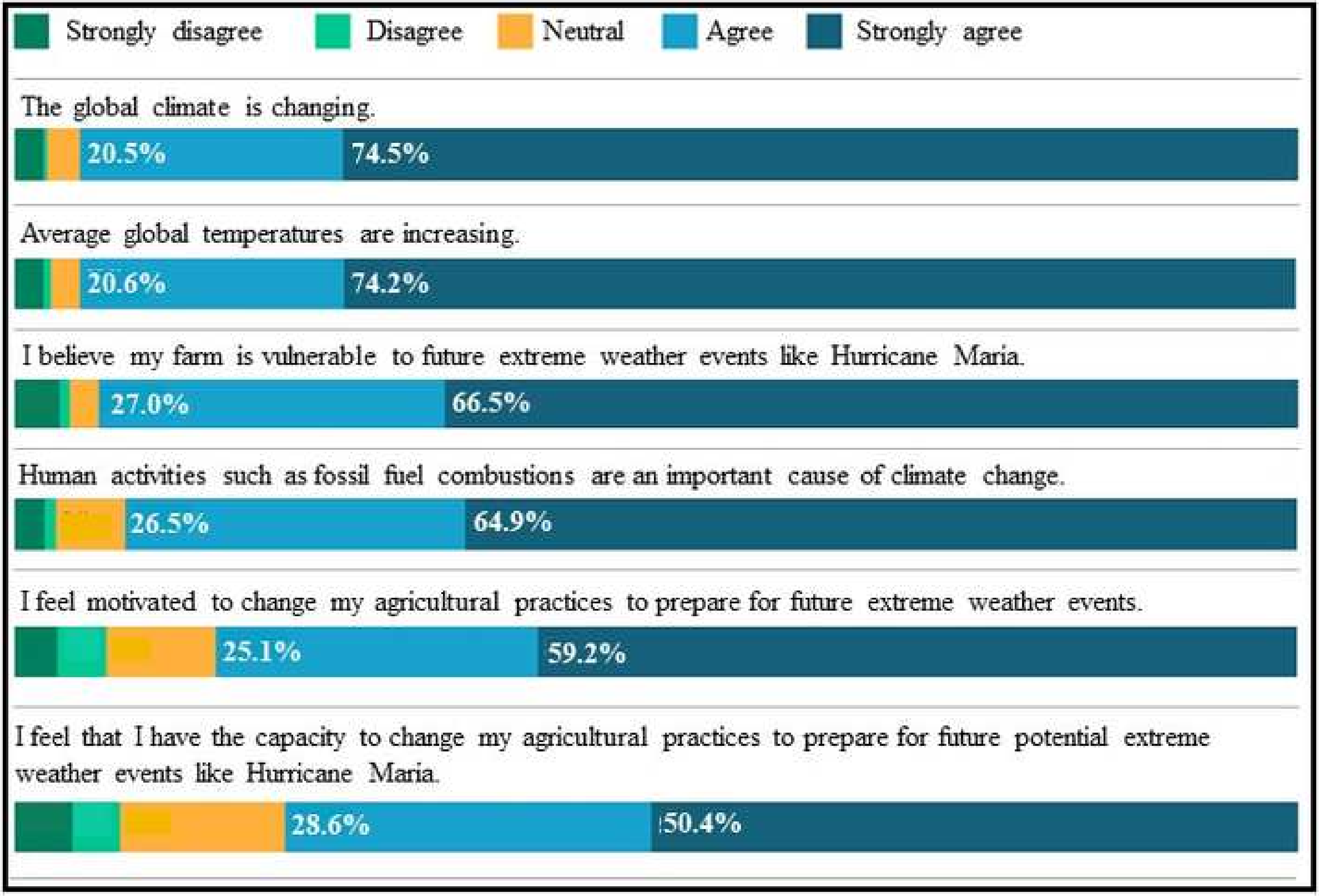

Fig 4_Farmers' psychological distance of climate change

Click here to download Figure Fig 4.tiff.tif

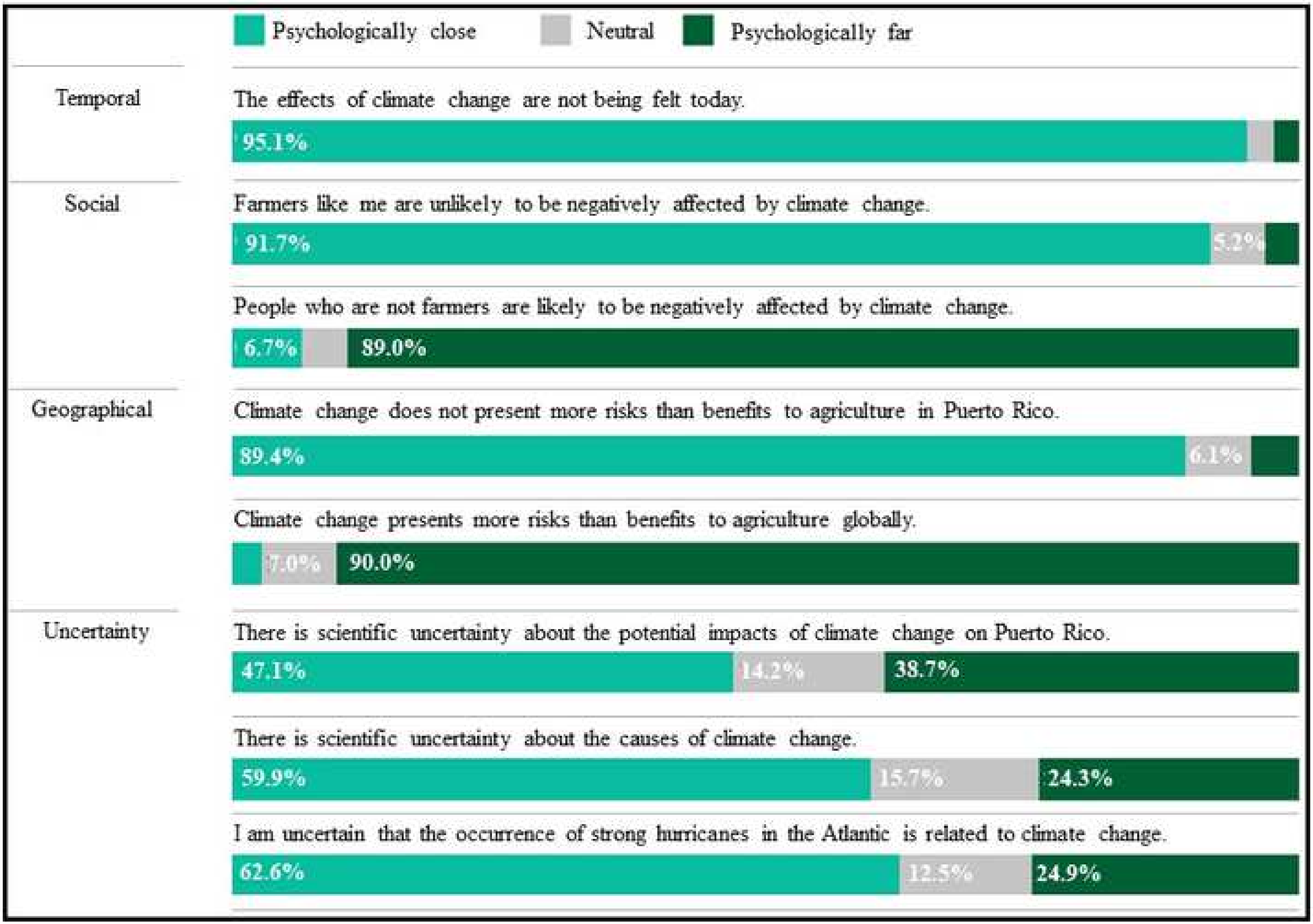

Fig 5_Structural equation model

Click here to download Figure Fig 5.tiff.tif

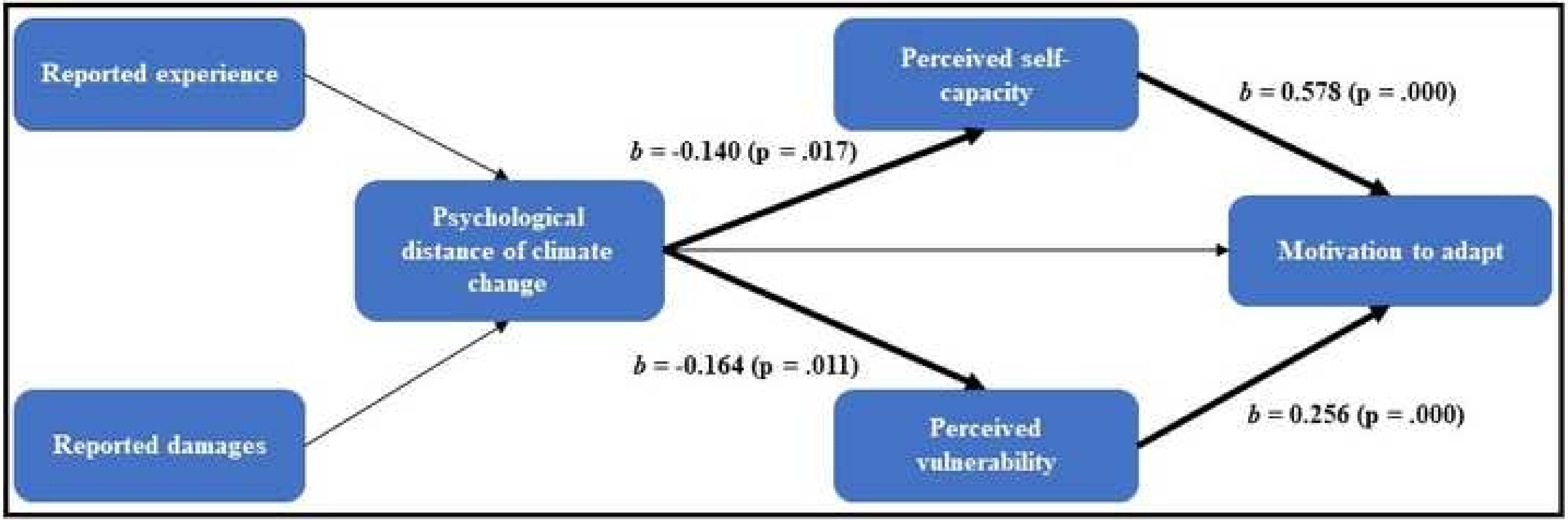

S1 table_structural equation model full results

Click here to access/download
Supporting Information
S1_Table.docx

S2 table_structural equation model indirect effects

Click here to access/download
Supporting Information
S2_Table.docx

S3 table_structural equation model total effects

Click here to access/download
Supporting Information
S3_Table.docx
