# Supplementary material for: Awareness of climate change's impacts and motivation to adapt are not enough to drive action: A look of Puerto Rican farmers after Hurricane Maria
Source: PLoS One. 2021 Jan 27;16(1):e0244512. doi: 10.1371/journal.pone.0244512 (PMC7840010; doi:10.1371/journal.pone.0244512)
Supplement: Attachment — Submitted filename: PONE-D-20-19200_R1.pdf [file pone.0244512.s008.pdf]

# PLOS ONE

# Puerto Rican farmers' psychological awareness of climate change, and adaptation perceptions after Hurricane Maria

--Manuscript Draft--

| | |
|---|---|
| **Manuscript Number:** | PONE-D-20-19200R1 |
| **Article Type:** | Research Article |
| **Full Title:** | Puerto Rican farmers' psychological awareness of climate change, and adaptation perceptions after Hurricane Maria |
| **Short Title:** | Farmers' psychological distance of climate change after a natural hazard |
| **Corresponding Author:** | Luis Alexis Rodríguez-Cruz, M.S.<br>University of Vermont<br>Burlington, VT UNITED STATES |
| **Keywords:** | psychological distance of climate change;  structural equation model;  natural hazards; small island developing states;  adaptive capacity |
| **Abstract:** | Understanding how experience with extreme events are linked to climate change adaptation’s social and cognitive drivers is key in strengthening adaptive capacity. Here we use the psychological distance of climate change as a framework to explore Puerto Rican farmers’ adaptation perceptions after Hurricane Maria. Farmers were surveyed eight months after the hurricane by Extension agents (n = 405, 87% response rate). A structural equation model assessed how reported experience with past extreme events and hurricane damages related to their psychological distance of climate change, and how these variables were linked to their adaptation perceptions. Results showed that Puerto Rican farmers have a high level of climate change belief, and a psychological distance of climate change that is both near and far, since they show a broad awareness to climate change’s impacts both locally and globally in different dimensions (temporal, spatial, and social). Further, we find that reported previous experience with similar extreme events to Maria, and intensity of hurricane damages caused by Maria were not related to their psychological distance of climate change, and these did not relate to their motivation to adapt. These results suggest that we may be reaching a tipping point for extreme events as a driver for climate belief and action, especially in a context where there is a high level of climate change belief and continued threat of extreme events. Instead, these results indicate that factors beyond individual perceptions and beliefs, such as structural, institutional or political factors, may be critical barriers to climate change adaptation. Thus, broadening analysis beyond individual perceptions as drivers of climate adaptation to how structural dynamics are linked to adaptive capacity may provide better understanding of adaptation barriers. This study is amongst the first to explore farmers’ psychological distance of climate change in an island-archipelago setting after an extreme event. |
| **Order of Authors:** | Luis Alexis Rodríguez-Cruz, M.S. |
| | Meredith T. Niles |
| **Response to Reviewers:** | Response to Reviewers<br><br>Dear Editor and Reviewers,<br><br>We thank you for taking the time and energy to review our manuscript. Your thoughtful comments and recommendations have been valuable in improving our manuscript’s clarity and rigor. Below you can find our responses to specific reviewer comments in bold. At the end, we also include the updated Competing Interests section, as requested in the PLOS ONE Decision email.<br>PONE-D-20-19200<br>Reviewer #1: This article reports the results of a survey of Puerto Rican farmers aimed at measuring their motivation to adapt to climate-exacerbated hazards (hurricanes) as a function of psychological distance, vulnerability and capacity. They also examine the effect of prior and recent (Maria) experience on distance through a structural equation model. While I found the study to be timely and an interesting contrast to the majority of the literature in this space (e.g., subsistence farmers in developing contexts, big scale |

commodity farms in developed contexts, etc), I have some issues with the theoretical framing and the conclusions that are drawn given the variability in the data and the way that variables were operationalized. I think perhaps the most interesting story in this data is this idea that psychological distance may not be a critical lens for understanding motivation in some contexts. The dominant narrative is to "bring the risk closer", but there is some evidence that this is not always better, and this data in particular demonstrates that the risk is both near and far, but that it is the perceived capacity to adapt that best explains motivation. I do agree with the author's conclusions that moving beyond individual or psychological variables may be necessary to really understand motivation and adaptation in contexts like this (and others). This is an argument made in a recent review in Nature Climate Change by Wilson et al.

While the analysis seems appropriate and rigorous, I do worry about the fit of the data for a parametric analysis - it is not normally distributed. And given that reality, along with some issues in measurement, it causes me to question some of the conclusions. I do not see that the data has been made available, but perhaps I missed this. And while the writing is fairly strong, there are some grammatical issues that need addressed. My specific line by line comments are in the attached PDF, but I would make these major suggestions.

1) Be more clear in your introduction about your theoretical framework. The intro focuses largely on psychological distance, and to some degree the role of experience, in explaining motivation to adapt. But then other variables are brought into play (vulnerability, capacity). Why are these included? Why are these the likely theoretical drivers of one's motivation to adapt? Why would we expect distance to help explain vulnerability and capacity? What have other studies found about this suite of variables? Again, you focus on experience and distance, but this must be a reason you included these other variables in your model. And given they turn out to be the most important variables, you need a better review up front. While this may be the first study of farmers in small island developing contexts, there are likely other studies that look at actual experience with a hazard and distance...this was not clear from your review. I think you need to think carefully about what background literature is necessary to situate your findings - and it seems that you need to speak to what is known about experience and distance broadly (and in particular actual experience), and then you need to speak to the role of distance, vulnerability and capacity on motivation. I would start by broadly summarizing what might be known in the adaptation literature at large, but then narrowing in on what is known specifically in the context of agriculture (where that exists).
Response: We have restructured our introduction, and have broadened specific lines, as well as in the literature review section, to be more specific about the extent to which experience and psychological distance relate, and about why we included the variables you mentioned. We focused on literature that used the psychological distance of climate change (PDCC) as its framework to understand behavior or literature that evaluated PDCC after an event. The main argument of such literature is that reducing psychological distance will prompt mitigation or adaptation behaviors. Here, instead of evaluating adoption of practices, for example, we assessed the extent to which PDCC is linked to reported levels of perceived capacity, vulnerability, and motivation to adapt, which are variables important in adaptive capacity research from a social and psychological perspective. We argued that in order to understand adaptation, which reflects adaptive capacity, we must understand social and cognitive drivers. Thus, we used PDCC to understand how Puerto Rican farmers perceive climate change after the brunt event of Hurricane Maria, how that then that was linked to sociodemographic and reported experience variables, and how PDCC was linked to other adaptation perceptions. Hence, our main goal in this paper was to assess variables that to the best of our understanding have not been studied in Puerto Rico. In the reviewed discussion we underscored that future studies should inquire more deeply about what explains farmers’ perceived capacity, vulnerability, and motivation to adapt from different angles, given that those variables have been shown to be pivotal in adaptation research. It is important to reiterate that this paper is about understanding farmers’ PDCC after a category 4 hurricane.
In terms of your comments regarding the discussion, we appreciate that you highlighted Wilson et al. (2020). We were not aware of that review paper, and we think that their argument aligns we our discussion. We included it in our discussion, thank you.

Regarding your comments on the structural equation model (SEM), we have used bootstrapping (n=1,000), a documented method to control for a number of issues in structural equation models, including non-normal data. We have clarified in the methods section that using Bootstrap in SEM is recommended for non-normal data. We also intend to share the non-identified data in a repository after the acceptance of the paper.

2) Similarly, you kind of gloss over some of the variables in your methods in terms of how they were operationalized. I also have some other issues with your operationalization and then the conclusions you draw. To what extent might experience have played a bigger role if it was a more nuanced measure? See excellent work by Julie Demuth on measuring experience with natural hazards, her work is largely on tornados, but might be worth perusing. I am not convinced that experience does not matter, it just seems that your metric of experience did not matter. One could argue that accumulated experience with hurricanes over time has moved climate change from a distant to a distant and near threat, but that your study does not capture this dynamic process. Greater clarification on your measures is necessary in general - why these items? Are they validated scales? Have they been used elsewhere?
Response: Thank you for your comments on experience and for bringing Demuth's work to our attention. We clarified in the paper about our use of the experience variables, both reported hurricane damages and reported experience with similar events. The word "reported" was added to be specific about the variables. Furthermore, in the reviewed discussion, we expanded on "experience" to capture your comments and recommendations. It was also included in the limitations section. Nevertheless, it is important to note that in in the literature review section (Research on the psychological distance of climate change), we highlighted the work by Zanocco et al. (2018) to inform why used reported damages as one measure of experience. Other PDCC studies have used the single reported experience scale, and other studies have been done in an area after an extreme event without asking about that experience. We clarified in the methods about the studies that informed our survey instrument, and how variables have been used in previous studies. Nevertheless, as Demuth shows, the use of different experience measures is a current barrier in existing studies. We recommended the further investigation of what it means to experience an extreme event in Puerto Rico, and we also added historical information on landing and non-landing hurricanes and tropical storms in Puerto Rico. It is important to note that our questions were framed to capture participants' opinions and perceptions around Hurricane Maria. Puerto Rico was still recovering when the survey was carried out. We also included a section to explain the assessment of social and cognitive variables, and we included more information on how the PDCC scale was built. Finally, we included supplementary material on the pilot's PDCC analysis, which in demonstrating reliable validity, was another factor in our design.

3) Finally, I think the discussion could better situate your findings in the context of other research on behavioral/psychological factors and climate adaptation, as well as other work on farmers and climate beliefs. You mentioned the van Valkengoed and Steg meta-analysis - how do your results match or not with their findings? You mention in your intro that experience was not significant int heir analysis, but what about your other variables? Vulnerability? Capacity? To what extent have these variables been studied in farmer specific studies? Also, there were several studies out of Purdue on Midwestern US farmer climate beliefs. It would be helpful to situate Puerto Rican farmers within this broader context of global agriculture - how are they similar? How are they different? It is not surprising to me that they are taking this threat more seriously, but that the biggest issue might be capacity to act. A more nuanced discussion of this decision process for this group vs. other farmer groups studied would be useful. Behavior change is often a function of both motivation and ability - Puerto Rican farmers seem to have the motivation (concern, perceived personal risk, etc) but not the ability. While other US farmers (like in big Midwest commodity ag) may lack that initial motivation or concern.
Response: Thank you for your comments and recommendations. We have expanded our discussion. We think that understanding the decision-making process around adaptation should be further looked into in future studies. We explain in our discussion why this is important, and how our results support those recommendations.
Reviewer #2: This paper provided a comprehensive understanding about the relationships among Puerto Rican farmers' psychological distance of climate change,

their experience of the category 4 Hurricane Maria and their perceptions of climate change. Then the paper explored further the association of these variables to farmers' motivation to climate change adaptation.

It is worth understanding the roles of extreme weather events experience and perceived psychological distance in farmers' decision making around climate change adaptation. Besides, this paper supplement a research in an island-archipelago setting to a more comprehensive view of perceptions and adaptation of climate change, which is valuable for future researches and practices.

However, there are some issues (such as the definition of psychological distance and its analysis in the models) that need to be better considered according to the survey and objective design. Also, the results need to be better interpreted for a more concrete conclusion. Below are my comments:

1. Main: about the psychological distance

In the Theoretical framework section (P6), it gave the explanation of psychological distance with the four components. Then according to the Objective, research questions, and hypotheses section (P11), it's mainly on how the degree of psychological distance (how far or how close) linked to the extreme experience or damage, and how it associates with the cognitive perceptions and motivation of adaptation. However, in Methodology on Assessing the psychological distance (P13, line 285) and the survey (Table 1), it redefined "psychologically far" and "psychologically close" for each item. There are some questions about it:

1 ) Line 285, "Strongly agree" and "agree" answers for dimensions' statements were combined as "psychologically far" — How about the reversed items? Usually, negatively worded statements were used as reversed items for controlling the response bias with reverse scoring. But here, it seems only the meaning of local or global were used as the reversed items, instead of negative statements. Need to be careful about this for the further analysis.
Response: Thank you for the opportunity to further clarify our method, and we apologize for the initial confusion. The psychological distance of climate change (PDCC) scale was developed from eight, separate 5-point Likert scale items. Your reference above is related to our presentation of results in figure 4. Statements were not combined for statistical analysis. Given that the scale measured ascending PDCC (e.g. the higher the number, the more distant the farmer perceives climate change), we presented the results as "psychologically far" for the combination of "agree" and "strongly agree" responses, and "psychologically close" for "disagree" and "strongly disagree responses". We have decided to eliminate that way of showing results to prevent confusion, and we clarified how we analyzed results in the Methods section.

2) According to such redefinition and analysis, it has regarded close and far as a one-dimensional variable (either close or far) instead of two-dimensional (how close and how far in those four components at the same time). Nevertheless, the design of the survey items divided close and far into two items, which made it a bit confusing. The results also showed the farmers could perceive both close and far. So maybe there is also a need to consider the close or far into two dimensions for analysis.
It could change to the exploration of the relationship between how close they perceived (the degree of close) in four components and their experience or beliefs. Further, how far they perceived (the degree of far) in those components need to be separated for the two items (General public and Global agriculture).
Response: All the items were evaluated as 5-point Likert scales. To ideally control for "straight-lining" in a survey, we intentionally had some questions worded oppositely so that we could conduct data quality checks.  Some questions were reversed so that responses were shown in ascending levels of PDCC. In terms of interpretation, we interpreted "agree" and "strongly agree" to be "psychologically far", and "agree" and "strongly agree" to be psychologically close. We have clarified in the manuscript how we analyzed and interpret these items and the PDCC scale.

3) Based on above, if focusing on the degree of psychological distance perceived, maybe keeping 5-point Likert scale coding for analyses would be suitable than 3-points.

Powered by Editorial Manager® and ProduXion Manager® from Aries Systems Corporation

Response: We clarified in the manuscript that they were interpreted and statistically analyzed as 5-point Likert scales.

4) on Line 284, it showed the Reliability Analysis with Cronbach's alpha value for the all items in psychological distance scale, could you also provide the Cronbach's alpha value for each component (more than one item) to see the internal consistency?
Response: We included in the Methods section more information on how we created the scale from the pilot study's data. We report reliability analysis with Cronbach's alpha and factor analysis results. Alpha is recommended for scales with three or more items. We expanded in our Methods section about what informed our variable use.

5) Besides Reliability Analysis, could you also provide the results of validity analysis, such as factor analysis? This could help to see whether the analysis of psychological distance scale could provide the support for the two dimensions on close and far, or four dimension on theoretical components. Then according to the factor analysis, it could be better consider whether still regard psychological distance as one variable in the models.

Based on the above, I suggest that the authors better consider and interpret the psychological distance and related results. Would they be confident of their final results that "the experiences were not linked to their psychological distance of climate change, and these did not relate to their motivation to adapt", when using the current ways on the definition and analyses.
Response: Thank you for your comments and recommendations. We better explained what we meant by "experience" in the paper, and we included historical records of landing and non-landing hurricanes in Puerto Rico to our discussion. We also performed a reliability analysis and factor analysis with the pilot data, which informed our PDCC analysis. We expanded on Methods, and included the new supplementary materials.

2. Main: about the experience (with events and damages)

1 ) Line 184-185, it seems the direct experience in this paper means the reported degree of damage. But how does one distinguish the reported experience from reported damage level? People could perceive the events and reported the experience through the damage experience. So, it is hard to say that those researches only used data on reported experience that were not related to direct experience, such as Spence's work cited in this paper.
Response: In our revision, we clarified on what we meant by experience, how we assessed it, and what can be done in the future to further understand what it means to experience an extreme weather event in Puerto Rico. We had two variables, one that asked about reported experience with similar events in the past 10 years, and one that asked about farm-level damaged caused by Maria. It is important to note that Hurricane Maria affected all of Puerto Rico. It has been the most devastating storm to hit Puerto Rico in 89 years. People were still recovering when the study was carried out. We also included historical records in the discussion to further expand on experience and PDCC.

2 ) Line 201, it said the paper would assess the extent to which the experience with Maria (both reported experience with other extreme events…). And in Objective section, the Objective 2 and H1 all mentioned the experience with Maria. While based on the only one item for the reported experience in table 1, is the statement framing mainly for the experience with other extreme weather events except Hurricane Maria or for the experience with Hurricane Maria?
Response: Thank you, we clarified the variables in the objectives, methods, and overall discussion.

3 ) If the reported experience is focusing on the experience with other extreme weather events except Hurricane Maria, could you provide more introduction on the extreme weather events happened in this area before Maria?
Response: We included data on historical records regarding hurricanes in the discussion.

4 ) Besides, it is needed to clarify the interpretation framing with the experience related

| | |
|---|---|
| | results, if the experience from the survey is not experiencing Maria, e.g. in Abstract "Reported experience and direct damages by the hurricane were not linked to their psychological distance".<br>Response: We clarified how we understand and assessed experience.<br><br>5 ) Line 180, it mentioned the extreme events may have become "more normal" and this view was also mentioned in discussion to explain the weak role of experience in the model. It also could be better if you could provide more background information on the historical extreme events the people there may experienced.<br>Response: We have included these suggestions.<br><br>Other comments:<br><br>3. line 267, could you clarify the ways that the surveys were randomly administered to farmers in Data gathering section?<br>Response: We expanded on this in the methods section.<br><br>4. Adding the Cronbach's alpha value into the table 1 if it is available.<br>Response: Cronbach's alpha for the main study's scale is included in the text of the Assessing the psychological distance of climate change section in Methods.<br><br>5. Adding the citation into table 1 if there are some items referring to other researches.<br>Response: Thank you for the recommendation. We added the citations in text.<br><br>6. Could combine table 2 and table 3 into one comprehensive table, then could consider to put it into supplementary sheet if there is more table/figures than requested.<br>Response: We have decided to keep the tables separate. Thank you for the recommendation.<br><br>Here is our update Competing Interests section:<br>"I have read the journal's policy and the authors of this manuscript have the following competing interests: MTN is a member of the board of directors of The Public Library of Science (PLOS). This role has in no way influenced the outcome or development of this work or the peer review process, nor does it alter our adherence to PLOS ONE policies on sharing data and materials. This does not alter our adherence to PLOS ONE policies on sharing data and materials." |
| **Additional Information:** | |
| **Question** | **Response** |
| **Financial Disclosure**<br><br>Enter a financial disclosure statement that describes the sources of funding for the work included in this submission. Review the submission guidelines for detailed requirements. View published research articles from *PLOS ONE* for specific examples.<br><br>This statement is required for submission and **will appear in the published article** if the submission is accepted. Please make sure it is accurate. | MTN allocated funding from this study, which came from the College of Agriculture and Life Science and the Food Systems Graduate Program of the University of Vermont. Include this sentence at the end of your statement: The funders had no role in study design, data collection and analysis, decision to publish, or preparation of the manuscript. |

| | |
|---|---|
| **Unfunded studies**<br>Enter: *The author(s) received no specific funding for this work.*<br><br>**Funded studies**<br>Enter a statement with the following details:<br>• Initials of the authors who received each award<br>• Grant numbers awarded to each author<br>• The full name of each funder<br>• URL of each funder website<br>• Did the sponsors or funders play any role in the study design, data collection and analysis, decision to publish, or preparation of the manuscript?<br>• **NO** - Include this sentence at the end of your statement: *The funders had no role in study design, data collection and analysis, decision to publish, or preparation of the manuscript.*<br>• **YES** - Specify the role(s) played.<br><br>* typeset | |
| **Competing Interests**<br><br>Use the instructions below to enter a competing interest statement for this submission. On behalf of all authors, disclose any competing interests that could be perceived to bias this work—acknowledging all financial support and any other relevant financial or non-financial competing interests.<br><br>This statement **will appear in the published article** if the submission is accepted. Please make sure it is accurate. View published research articles from *PLOS ONE* for specific examples. | I have read the journal's policy and the authors of this manuscript have the following competing interests: MTN is a member of the board of directors of The Public Library of Science (PLOS). This role has in no way influenced the outcome or development of this work or the peer review process, nor does it alter our adherence to PLOS ONE policies on sharing data and materials. |

| | |
|---|---|
| **NO authors have competing interests**<br><br>Enter: *The authors have declared that no competing interests exist.*<br><br>**Authors with competing interests**<br><br>Enter competing interest details beginning with this statement:<br><br>*I have read the journal's policy and the authors of this manuscript have the following competing interests: [insert competing interests here]*<br><br>* typeset | |
| **Ethics Statement**<br><br>Enter an ethics statement for this submission. This statement is required if the study involved:<br><br>• Human participants<br>• Human specimens or tissue<br>• Vertebrate animals or cephalopods<br>• Vertebrate embryos or tissues<br>• Field research<br><br>Write "N/A" if the submission does not require an ethics statement.<br><br>General guidance is provided below. Consult the submission guidelines for detailed instructions. **Make sure that all information entered here is included in the Methods section of the manuscript.** | The Committees on Human Subjects Serving the University of Vermont and the UVM Medical Center at the Research Protections Office approved our study on 04-Dec-2017. The study (CHRBSS: 18-0258) received Exemption Category 2. The consent obtained from the participants was oral. The consent was part of the survey booklet. |

Powered by Editorial Manager® and ProduXion Manager® from Aries Systems Corporation

| | |
|---|---|
| **Format for specific study types**<br><br>**Human Subject Research (involving human participants and/or tissue)**<br>• Give the name of the institutional review board or ethics committee that approved the study<br>• Include the approval number and/or a statement indicating approval of this research<br>• Indicate the form of consent obtained (written/oral) or the reason that consent was not obtained (e.g. the data were analyzed anonymously)<br><br>**Animal Research (involving vertebrate animals, embryos or tissues)**<br>• Provide the name of the Institutional Animal Care and Use Committee (IACUC) or other relevant ethics board that reviewed the study protocol, and indicate whether they approved this research or granted a formal waiver of ethical approval<br>• Include an approval number if one was obtained<br>• If the study involved *non-human primates*, add *additional details* about animal welfare and steps taken to ameliorate suffering<br>• If anesthesia, euthanasia, or any kind of animal sacrifice is part of the study, include briefly which substances and/or methods were applied<br><br>**Field Research**<br><br>Include the following details if this study involves the collection of plant, animal, or other materials from a natural setting:<br>• Field permit number<br>• Name of the institution or relevant body that granted permission | |
| **Data Availability**<br><br>Authors are required to make all data underlying the findings described fully available, without restriction, and from the time of publication. PLOS allows rare exceptions to address legal and ethical concerns. See the PLOS Data Policy and FAQ for detailed information. | No - some restrictions will apply |

| | |
|---|---|
| A Data Availability Statement describing where the data can be found is required at submission. Your answers to this question constitute the Data Availability Statement and **will be published in the article**, if accepted.<br><br>**Important:** Stating 'data available on request from the author' is not sufficient. If your data are only available upon request, select 'No' for the first question and explain your exceptional situation in the text box.<br><br>Do the authors confirm that all data underlying the findings described in their manuscript are fully available without restriction? | |
| **Describe where the data may be found in full sentences. If you are copying our sample text, replace any instances of XXX with the appropriate details.**<br><br>• If the data are **held or will be held in a public repository**, include URLs, accession numbers or DOIs. If this information will only be available after acceptance, indicate this by ticking the box below. For example: *All XXX files are available from the XXX database (accession number(s) XXX, XXX.)*.<br>• If the data are all contained **within the manuscript and/or Supporting Information files**, enter the following: *All relevant data are within the manuscript and its Supporting Information files.*<br>• If neither of these applies but you are able to provide **details of access elsewhere**, with or without limitations, please do so. For example:<br><br>*Data cannot be shared publicly because of [XXX]. Data are available from the XXX Institutional Data Access / Ethics Committee (contact via XXX) for researchers who meet the criteria for access to confidential data.*<br><br>*The data underlying the results presented in the study are available from (include the name of the third party* | Data cannot be shared publicly because only a portion of the data was used in the reported study. We will release the de-identified data that we have used in the manuscript to a data repository upon acceptance of the article. |

| | |
|---|---|
| *and contact information or URL).*<br>• This text is appropriate if the data are owned by a third party and authors do not have permission to share the data.<br><br>* typeset | |
| Additional data availability information: | |

Powered by Editorial Manager® and ProduXion Manager® from Aries Systems Corporation

Manuscript Click here to access/download;Manuscript;Manuscript.docx

# Puerto Rican farmers' psychological awareness of climate change, and adaptation perceptions after Hurricane Maria

Luis Alexis Rodríguez-Cruz[1,3*] & Meredith T. Niles[2,3]

[1]Food Systems Graduate Program, University of Vermont, Burlington, Vermont

[2]Department of Nutrition and Food Sciences and Food Systems Graduate Program, University of Vermont, Burlington, Vermont

[3]Gund Institute for the Environment, University of Vermont, Burlington, Vermont.

*Corresponding author

E-mail: lrodrig2@uvm.edu (LARC)

LARC contributions: conceptualized research, survey development, project liaison, data entry and analysis, wrote manuscript draft.

MTN contributions: Conceptualized research, survey development, funding acquisition, data analysis, edited manuscript draft.

# Abstract

Understanding how experience with extreme events are linked to climate change adaptation's social and cognitive drivers is key in strengthening adaptive capacity. Here we use the psychological distance of climate change as a framework to explore Puerto Rican farmers' adaptation perceptions after Hurricane Maria. Farmers were surveyed eight months after the hurricane by Extension agents (n = 405, 87% response rate). A structural equation model assessed how reported experience with past extreme events and hurricane damages related to their psychological distance of climate change, and how these variables were linked to their adaptation perceptions. Results showed that Puerto Rican farmers have a high level of climate change belief, and a psychological distance of climate change that is both near and far, since they show a broad awareness to climate change's impacts both locally and globally in different dimensions (temporal, spatial, and social). Further, we find that reported previous experience with similar extreme events to Maria, and intensity of hurricane damages caused by Maria were not related to their psychological distance of climate change, and these did not relate to their motivation to adapt. These results suggest that we may be reaching a tipping point for extreme events as a driver for climate belief and action, especially in a context where there is a high level of climate change belief and continued threat of extreme events. Instead, these results indicate that factors beyond individual perceptions and beliefs, such as structural, institutional or political factors, may be critical barriers to climate change adaptation. Thus, broadening analysis beyond individual perceptions as drivers of climate adaptation to how structural dynamics are linked to

adaptive capacity may provide better understanding of adaptation barriers. This study is amongst the first to explore farmers' psychological distance of climate change in an island-archipelago setting after an extreme event.

# Introduction

Exposure, sensitivity, and adaptive capacity are three main determinants of farmers' vulnerability to natural hazards [1,2]. Farmers in small island states and territories, most of which are located in the tropics, farm in regions that are often disproportionately exposed and sensitive to natural hazards [2–4]. Exposure refers to islands being situated in regions highly prone to extreme weather events (e.g. Atlantic's 'Hurricane Belt'), and sensitivity relates to the degree to which such hazards change physical systems (e.g. farms) [1,4–6]. As such, strengthening adaptive capacity—defined as the set of abilities and resources individuals and populations have to anticipate, withstand, cope with, and recover from a hazard, and the potential or abilities they have to modify the system in order to be more resistant to impacts [3,7,8]—is key in decreasing vulnerability to natural hazards.

Adaptive capacity is complex, and involves many diverse and interrelated determinants [3,4]. In the case of farmers, the adoption of new adaptation technologies and practices, as well as the access to institutional resources and networks of support, are important for strengthening adaptive capacity [9,10,11–13]. Research in the Caribbean and Central America—areas exposed to Atlantic hurricanes—have shown that farms and farmers' characteristics, as well as their access to diverse markets and sources of support are important in reducing vulnerability to natural hazards [9,14–17]. Nevertheless, given the heterogeneity of the regions' sociopolitical

systems, context-specific research is important to better understand adaptive capacity [4,18]. This is evident in the Caribbean, given the region's neocolonial dynamics, where many islands' sovereignty resides on continental countries [19–21]. This is the case of Puerto Rico as an Unincorporated Territory of the United States. Studying Puerto Rican farmers' climate change adaptation could allow us to understand how broader sociopolitical structures reflect in the process of strengthening adaptive capacity.

Understanding the role of perceptions and beliefs as determinants of adaptive capacity is key to determining effective strategies [3,6,22] . As such, it is important to understand to what extent beliefs and perceptions are linked in farmers' decision-making processes around climate change adaptation, which reflects adaptive capacity [5,6,11]. Climate change adaptation occurs at different scales [11,23], and it is contextualized, highlighting that understanding the degree to which cognitive beliefs impact individuals' decision-making and willingness to act is important in assessing adaptive capacity [5,24,22,25]. Studies have found that individuals' perceived capacity, perceived vulnerability, motivation to adapt, and other social and cognitive variables are key in understanding adaptation behaviors [10,22,24,25]. The ways farmers perceived their capacity to adopt new adaptation behaviors, and the risks they are exposed to have been shown to drive the adoption of new agricultural practices and pro-environmental behaviors [10,24,26–28]. While existing research demonstrates a link between climate perceptions, beliefs, and climate change adaptation amongst the general public [29], and farmers [24], there are varied results [25,30,31]. Importantly, most research focused on perceptions about climate and the role of cognitive beliefs in climate adaptation is from high-income countries [25]. There is limited evidence of this topic from small island developing states and territories, whose unique exposure

and sensitivity to climate change and extreme events may influence how residents perceive climate change and their adaptive capacity.

To contribute to fill the current gaps in the literature, we examine survey results from 405 Puerto Rican farmers following Hurricane Maria, a category four hurricane, which impacted Puerto Rico in September 2017. We examine farmers' perceptions and beliefs about climate change, its potential link to their reported damages from the hurricane as a proxy for direct experience, their reported past experience with similar events, and the extent to which these perceptions and beliefs influence their motivation to adapt to climate change. We use the theoretical framework of the psychological distance of climate change—simply put, how near or far people perceive climate change to be from themselves in various contexts [29,32,33,34]. We intend to evaluate the degree to which the psychological distance of climate change amongst Puerto Rican farmers manifests after the brunt event of a hurricane. This baseline work can expand our understanding of the role of cognitive beliefs and perceived psychological distance for prompting adaptation behaviors to strengthen adaptive capacity amongst individuals beyond high-income and continental countries. Furthermore, it gives us the ability to generate novel information for scholarship focused on farmers' adaptive capacity in Puerto Rico as well.

# Theoretical framework

Construal Level Theory states that humans imagine the past and future through abstract constructs, since we are embedded in the here and now [33,34]. Psychological distance, a component of the aforementioned theory, is a subjective experience that uses the self as a

reference point [33,34]. Psychological distance is measured in four components: temporal, social, spatial, and hypothetical (uncertainty of the distance or issue). The more distant we perceive an issue or event to be across these dimensions, the more we rely on abstract constructs to make sense of it. In contrast, things we perceive as closer are more concrete. For example, as an event gets closer in time (e.g. conference presentation), the awareness of the dynamics involved become more concrete (e.g. transportation to the venue, nervousness, time management, and other variables). Thus, Construal Level Theory explains that we use mental constructs to build understanding of what transcends the here and now.

Applied in the realm of climate change communication and adaptation research, the psychological distance of climate change [32] refers to how near or far people (e.g. farmers) perceive climate change to be from themselves across the four dimensions: temporal (when climate change will occur or if is occurring), spatial (where it happens), social (to whom it happens), and the hypothetical or uncertainty dimension (certainty of whether it occurs or not). Theoretically, this posits that individuals who are psychologically close to climate change are prone to perceive higher risks to hazards, and a greater motivation to act to adapt or mitigate climate change. Nonetheless, this assumption has not been consistent in research [30,31].

## Research on the psychological distance of climate change

The aforementioned hypothesis has been the basis for many existing studies on the psychological distance of climate change (e.g. understanding how psychological distance affects the propensity for individuals to take action to mitigate or adapt to climate change). The current body of

research focused on the psychological distance of climate change is both experimental [35,36], and observational [32,37]. More specifically, scholarship has explored (1) the interrelatedness of the different dimensions, (2) the role of experiencing extreme weather events on individuals' psychological distance of climate change, and (3) the degree to which psychological distance of climate change affects behavior. Overall, evidence indicates inconsistent outcomes related to the psychological distance of climate change and its capacity to prompt action, as well as the understanding of how experiences with extreme events affects psychological distance, and the relatedness of the four dimensions varies [31]. These results may suggest that context matters; a natural hazard (e.g. hurricane), for example, can be experienced and perceived differently depending on context (e.g. sociodemographic characteristics, beliefs, adaptive capacity) [1,2]. Some studies support that the four dimensions of the psychological distance of climate change are interrelated. Moreover, studies suggest that being psychologically close impacts behavior. For example, Spence and colleagues (2011, 2012) demonstrated that reported experience with floods was linked to individuals' perceptions of climate change in the United Kingdom. Their studies showed that the four dimensions of the psychological distance of climate change were interrelated, and that reduced psychological distance related to prompting  climate change mitigation actions amongst individuals [32,29]. Similarly, but based in the United States, Singh et al. (2017) found that the four dimensions were interrelated, and that those psychologically close expressed more support towards climate change adaptation policies. In that study, psychological distance was linked to experience with extreme events. The temporal and uncertainty dimensions, by their own, did not have significant effect on policy support; however, those that expressed higher uncertainty over climate change and were socially distant, expressed less support towards policies [37].Furthermore, research has shown that the dimensions, though

often interrelated, depending on context, manifest differently [31]. Focused on New Zealand's farmers, Niles et al. (2015) applied the psychological distance of climate change framework in combination with Liebig's Law of the Minimum to assess adaptation behaviors, finding that decreasing psychological distance may prompt action from farmers. Farmers who perceived climate change impacts as spatially close, were more likely to feel more concern for climate change and motivation to adapt [24].

Research has also described how experience with extreme weather events relates to being psychologically close [24,29,31,37]. Acharibasam and Anuga (2018) found that farmers who had experienced extreme events were socially and spatially close, and that psychological distance mediated emotional regulation among farmers—the mental processes through which individuals decide how emotions are expressed and experienced [38]. Nevertheless, a recent meta-analysis performed by van Valkengoed and colleagues (2019) found that experience had small-to-moderate effects on climate change adaptation. Moreover, Larcom (2019) found that experience with a heatwave amongst the general population did not elicit adaptation behaviors in the United Kingdom, but did elevate concern [39]. Similarly, Albright and Crow (2019) found that direct flooding experience was not a predictor of concern for climate change amongst Colorado, United States, residents [40]. These more recent studies suggest that there may be a threshold of individual exposure to natural hazards and their potential to catalyze behavioral change; indeed, as the rate of extreme events increases, it could be probable that such events may saturate in people's experiences and become "more normal".

The aforementioned studies also highlight the importance of context, but also bring attention to how psychological distance of climate change research is limited in its generalizability [30]. Furthermore, most research on the matter has used data on reported experience, and not on direct

experience (e.g. material impacts such as damage). Zanocco et al. (2018) performed case studies of residents whose communities were directly impacted by extreme events, such as tornados and wildfires, or were proximate to where the events occurred, in order to understand the relationship between experience of extreme event and views of climate change. Applying Construal Level Theory perspectives of psychological distance, they found that spatial proximity to the event did not align with climate change views, but reported harm did [41]. This quote from Demski et al. (2017), as cited by Zanocco et al. (2018), underscores the aforementioned: “Self-reported measures of experience that include: direct physical experience and material impacts of an event, well-defined in terms of personal effects and damage, are less susceptible to biased reporting, providing the best proxy available for objective experience” (p. 152) [41] .

This paper attempts to fill several gaps in the research.. First, we will assess Puerto Rican farmers’ psychological distance of climate change after experiencing an extreme weather event. Hurricane Maria affected all of Puerto Rico in September 2017, causing significant damages to infrastructure, agriculture, and livelihoods. The 2017 Atlantic Hurricane Season is noted for being a devastating one in the Caribbean region [42]. To the best of our understanding, there are no studies exploring the psychological distance of climate change amongst farmers in small island developing states and territories after an extreme weather event. Second, we will assess the extent to which ~~reported experience with Maria (~~both reported experience with other similar extreme events, and direct damages reported by Hurricane Maria~~)~~ relate to psychological distance of climate change. As stated above, there are mixed results on how reported experience and actual experienced damages are linked to climate change perceptions. Third, in order to increase our understanding on the degree to which cognitive beliefs play a role in climate change adaptation and therefore relate to individual adaptive capacity, we examine farmers motivation to

adopt adaptation practices, and its relationship to perceived capacity and vulnerability, as well to their psychological distance of climate change.

## The present study

This study focuses in Puerto Rico, an unincorporated territory of the United States that imports around 85% of the food it consumes, and that is going through a social-economic crisis because of its billion dollar debt and political situation [42–45]. In response to Puerto Rico's debt crisis in 2016 , the United States' government created a Fiscal Management and Oversight Board that imposed austerity measures, and made visible Puerto Rico's lack of political agency as a territory of the United States [19,20]. Simultaneous and previous to this change, the agricultural sector in Puerto Rico was experiencing positive changes, such as a rise in new farmers, production increases, and increased awareness of food security [14,45–47]. Such agricultural gains were important since Puerto Rico, like the broader Caribbean, was experiencing a steady decline in farms since the 1990's due to trade liberalization, globalization, and other external forces [,15,47, 48, 49]. Nevertheless, such gains were erased when both Hurricane Irma and Hurricane Maria, two of the strongest in the Atlantic's history, hit Puerto Rico in September 2017 [42,51–53]. The local Department of Agriculture (2018) reported that both hurricanes caused $2 billion in total losses, with the majority of losses from Maria ($228 million in production losses, and $1.8 billion in agricultural infrastructure losses). Such damages significantly impacted Puerto Rican agriculture, which, according to the USDA, is comprised of many small-scale farmers with an average farm size of 59 acres (approximately 23 hectares) [50], who focus mostly on domestic

markets, and who have an average annual income of less than $20,000 [49]. Focusing this study on Puerto Rican farmers after experiencing a major storm, not only allow us to advance our understanding of the psychological distance of climate change, but provides us the opportunity to produce important applied knowledge around strengthening adaptive capacity to safeguard local agriculture and food access [24,51].

## Objective, research questions, and hypotheses

Our study focuses on understanding how the psychological distance of climate change manifests in an island-archipelago setting, amongst a vulnerable population (farmers). Specifically, we intend to assess (1) the psychological distance of climate change amongst farmers in Puerto Rico, an unincorporated territory of the United States and Caribbean non-UN member Small Island Developing State, after an extreme-weather event, (2) the extent to which psychological distance of climate change is related to reported experience, and reported damages caused by Maria, and to (3) other cognitive beliefs important in adaptation research, such as motivation to adapt, perceived capacity (self-efficacy or perceived instrumentality), and perceived vulnerability. We ask: 1) How do farm and farmer characteristics, and experiences with extreme events (i.e. reported damages and reported experience with similar events) relate to the psychological distance of climate change? And, 2) in light of experiencing an extreme event, what is the role of psychological distance of climate change in motivating Puerto Rican farmers to adapt to climate change? Following research on the psychological distance of climate change, we hypothesize that farmers' psychological distance of climate change will be driven by their

experience (e.g. reported damages) with Hurricane Maria (H1). Furthermore, we predict that psychological distance of climate change will be related to perceived capacity, perceived vulnerability, and motivation to adapt (H2). Fig 1 shows our hypothesized model.

**Fig 1. Hypothesized model.** Farm and farmer characteristics' variables included as controls.

# Methodology

## Data gathering

Previous studies of on-farm management and climate change perceptions, and on the psychological distance of climate change [13, 24,37,52] informed the development of a survey instrument, which was contextualized to Puerto Rico and the general objectives of the main study. This study was carried out in collaboration with the Extension Service of the University of Puerto Rico at Mayagüez. Thus, feedback from Extension partners were also included in the survey's development. The survey was translated into Spanish, and piloted in February 2018 with a pool of Puerto Rican farmers (n = 32), and was also shared amongst Extension agricultural agents for feedback—enumerators of the survey. Minimal language and structural changes were made to the instrument.

Surveys (n = 440) were randomly administered to farmers by Extension agents, according to the five regions the Extension Service divides Puerto Rico: Arecibo, Caguas, Mayagüez, Ponce, and

San Juan. Each region has several agents that provide service to the municipalities that comprise each region. Survey deployment was based on Extension Services' recommendations to access a wide range of farmers, and because agricultural agents had an established presence. Each regional office received a set of surveys; San Juan, Caguas, and Ponce received 100 surveys each, and Mayagüez and Arecibo received 75 surveys each. Participant Extension agents randomly surveyed farmers in the municipalities they worked in. Surveys were administered between May and July 2018 in person. Farmers from 65 of Puerto Rico's 78 municipalities answered the survey. A total of 405 surveys were completed, resulting in an 87% response rate based on the American Association for Public Opinion Response Rate Calculator [53].

## Assessing the psychological distance of climate change

Eight questions were used to assess the four dimensions of the psychological distance of climate change (temporal, social, spatial, and uncertainty) (Table 1). These questions were adapted from previous research on the topic, and contextualized to Puerto Rico [24,32,37,52]. The temporal dimension was assessed with one item, while the social and spatial dimensions were assessed with two each. Questions assessing the social and spatial dimensions were intended to separately assess local and distant concerns. Uncertainty was assessed through three statements. Variables were reversed where needed, in order to analyze ascending levels of the psychological distance of climate change; "5" in the scale would represent "psychologically distant". We ~~utilized a~~ Cronbach's alpha to measure internal validity of these constructs in the pilot analysis, demonstrating good internal reliability (alpha=0.86). Furthermore, a factor analysis using

principle components was carried out on these items to further assess consistency. S1 Table shows the pilot data's analysis results, which indicate all items aligned well under one factor (Factor loadings were >.40). This supported our decision to keep a variable scale in the study —Cronbach's alpha for the main study's psychological distance of climate change scale was 0.74, which suggests good internal validity [58].

## Assessing social and cognitive variables

Adaptation is a set of contextualized decisions that reflect individuals' adaptive capacity.. Given the role of social and cognitive variables in predicting adaptation behaviors amongst farmers [10,22,24,26,52], we included several variables that, to the best of our knowledge, have not been evaluated in Puerto Rico. Farmers were asked about their motivation to adapt to climate change through adopting new agricultural practices, their perceived capacity to do so, and their perceived vulnerability to extreme events (Table 1).

## Structural equation model

A structural equation model was built to explore the extent to which psychological distance of climate change relates to farmers' reported experience with similar events to Hurricane Maria and reported degree of damage due to Maria, and to their motivation to adapt to climate change. Structural equation models allow quantitative analysis of the direct, indirect, and mediated interactions between variables, and the incorporation of latent constructs [28, 55, 56]. Fig 1 shows the hypothesized model. Two variables assessed experience: "reported past experience" and "reported hurricane damages" (Table 1). Variables for perceived capacity and vulnerability,

as well as for motivation to adapt, were included. Those variables, alongside psychological distance of climate change, were included to grasp an understanding of the role of cognitive beliefs in the context of adaptive capacity. The structural equation model was deployed in Stata 15.1, using Maximum Likelihood with bootstrapping (1000 iterations) to control for Type I errors, for non-normal data, and to increase statistical power [28,57,58].The following Goodness-of-fit criteria were used: Chi-square (X2), Standardized Root Mean Square Residual (SRMR), Comparative fit index (CFI), Root mean square error of approximation (RMSEA) [56].

**Table 1: Survey variables used in this study.**

| Category | Variable | Question/Statement | Measure |
|---|---|---|---|
| Climate change perceptions | Global climate | The global climate is changing. | 5-point Likert scale—from strongly disagree to strongly agree |
| | Global temperature | Average global temperatures are increasing. | 5-point Likert scale |
| | Anthropogenic causes | Human activities such as fossil fuel combustions are an important cause of climate change. | 5-point Likert scale |
| Psychological Distance of Climate Change | | | |
| Temporal Dimension | Today | The effects of climate change are already being felt today. | 5-point Likert scale |
| Spatial dimension | Local agriculture | Climate change does not presents more risk than benefits to agriculture in Puerto Rico. | 5-point Likert scale |

| Category | Variable | Question/Statement | Measure |
| --- | --- | --- | --- |
| | Global agriculture | Climate change presents more risks than benefits to agriculture globally. | 5-point Likert scale |
| Social dimension | Farmers | Farmers like me are not likely to be affected negatively by climate change. | 5-point Likert scale |
| | General public | People who are not farmers are likely to be affected negatively by climate change. | 5-point Likert scale |
| Hypothetical or uncertainty dimension | Impact uncertainty | There is scientific uncertainty about the potential impacts of climate change on agriculture. | 5-point Likert scale |
| | Cause uncertainty | There is scientific uncertainty about the causes of climate change. | 5-point Likert scale |
| | Hurricane uncertainty | I am uncertain that the occurrence of strong hurricanes in the Atlantic is related to climate change. | 5-point Likert scale |

| Category | Variable | Question/Statement | Measure |
| --- | --- | --- | --- |
| Cognitive beliefs | Motivation to adapt | I feel motivated to change my agricultural practices to prepare for future extreme weather events like Hurricane Maria. | 5-point Likert scale |
| | Perceived self-capacity | I feel that I have the capacity to change my agricultural practices to prepare for future potential extreme weather events like Hurricane Maria. | 5-point Likert scale |
| | Perceived vulnerability | I believe my farm is vulnerable to future extreme weather events like Hurricane Maria. | 5-point Likert scale |
| Experience with extreme weather events | Reported experience | I have faced similar extreme weather events like Hurricane Maria in the past ten years. | 5-point Likert scale |
| | Reported damages | How would you describe the damages, if any, caused by Hurricane Maria to your farm? | 5-point Likert—from no damages to total loss |
| Farmer and farm characteristics* | Age | In which year you were born? | Continuous |

| Category | Variable | Question/Statement | Measure |
|---|---|---|---|
| | Bonafide[a] | Do you participate in the bonafide program of the Puerto Rico Department of Agriculture? | Binary—yes or no |
| | Education level | What is the highest level of education you have completed? | Ordinal—from elementary school to PhD degree |
| | Farm size | How many *cuerdas*[1] of terrain do you manage in your farm? | Continuous |
| | Gender | What is your gender? | Binary—female or male |
| | Household income | What is your approximate household income, including all far and off-farm income? | Ordinal—from Less than $20,000 to more than $90,000 |

[a]The bonafide[a] program of the Puerto Rico Department of Agriculture provides farmers with agricultural incentives, and benefits then with various tax exemptions.

# Results

## Participants and the impact of Hurricane María

Farmers had an average age of 54, and farmed an average of 58 *cuerdas* (approximately 56 acres). The great majority of respondents had attended college (67%) (Table 2). Table 3 contrasts farmers' demographics and farm characteristics with those reported in Puerto Rico's 2017 Agricultural Census [50]. Respondents generally were similar to average census statistics, with the exception of having higher rates of formal higher education and somewhat higher percentage of income from farming. It is important to note that the recent census is not comparable to the previous one done in 2012 [50]. Moreover the 2017 census, performed in 2018, had a lower response rate, and USDA reported some difficulty in performing the census given post Hurricane Maria conditions [50,59]. Farmer respondents were distributed throughout Puerto Rico with 15% in the Arecibo region, 22% in the Caguas region, 19% in the Mayagüez region, 25% in the Ponce region, and 22% in the San Juan region. (Percentages do not sum 100% because some farms extend beyond one municipality that border with another). The majority of farmers experienced damages from Hurricane María; 42.6% reported total loss of their farms, while 45.6% reported significant damages (Fig 2). The majority of farmers (55.1%) disagreed and strongly disagreed that they had faced similar events to Hurricane Maria.

**Table 2: Mean statistics of control variables.**

| Variable | Scale | Frequency (%) | Mean ± SD | n |
|---|---|---|---|---|
| Age | Continuous | - | 54.0 ± 13.5 | 391 |
| Bonafide | Yes | 210 (52.8) | - | 398 |
| | No | 188 (47.2) | | |
| Education level | Elementary School | 21 (5.2) | - | 401 |
| | Junior High School | 13 (3.2) | | |
| | Some High School | 15 (3.7) | | |
| | High School Diploma | 82 (20.5) | | |
| | Some College | 42 (10.5) | | |
| | Technical Degree | 25 (6.2) | | |
| | Associate Degree | 41 (10.2) | | |
| | Bachelor’s Degree | 109 (27.2) | | |
| | Master’s Degree | 46 (11.5) | | |
| | PhD | 7 (1.8) | | |
| Farm size | Continuous | - | 58.1 ± 98.5 | 383 |
| Gender | Female | 55 (14.0) | - | 395 |
| | Male | 340 (86.0) | | |
| Household income | Less than $20,000 | 138 (36.4) | - | 379 |
| | $20,000 - $40,999 | 125 (33.0) | | |
| | $41,000 - $60,999 | 52 (13.7) | | |
| | $61,000 - $80,999 | 36 (9.5) | | |

| Variable | Scale | Frequency (%) | Mean ± SD | n |
|---|---|---|---|---|
| | More than $90,000 | 28 (7.4) | | |

**Table 3: Demographic variables of our study's surveyed farmers in comparison to USDA 2017 Agricultural Census data for Puerto Rico.**

| Category | Present study | USDA 2017 Census data |
|---|---|---|
| Average farm size | 58.1 *cuerdas* (majority farmed > 20 *cuerdas*) | 59.3 *cuerdas* (majority farmed > 20 *cuerdas*) |
| Years farming | Majority > 10 years | Majority > 10 years |
| Average age | 54 | 61 |
| Gender | Great majority is male | Great majority is male |
| Education | Majority reported High School Diploma or more | Majority reported High School Diploma or less |
| Income from farming | > 50% | < 50% |
| Household income | Majority reported < $20,000 | Majority reported < $20,000 |

Note: Data from the census is for farms' principal operators. In Puerto Rico, this does not necessarily mean that they are the sole owner of the farm. Neither the census or the local Department of Agriculture detail the number of Puerto Rican farmers who participate in the bonafide program; however, in a media report in 2019 (64), according to the Secretary of Agriculture, Carlos Flores, among Puerto Rico's 17,000 farmers, 4,000 are part of the bonafide program; 24% in comparison to the 53% in our study. It is also important to note that the Secretary's comment contrast with the number of farmers provided by the USDA census, which was conducted in 2018, and had a low response rate (54).

**Fig 2: Farmers' past experience with events similar to Hurricane Maria (top) and reported hurricane damages (bottom).**

Fig 3 shows results for farmers' perceptions around climate change and adaptation. An overwhelming majority of Puerto Rican farmers believe that the global climate is changing (86% strongly agree and agree), and that it has anthropogenic causes (91.4%). As well, 84.4% of Puerto Rican farmers agreed and strongly agreed that they feel motivated to change their agricultural practices to better prepare for future extreme events. Though farmers perceive their farms to be vulnerable to future extreme weather events (93.5%), they understand themselves capable to adapt to climate change (79.0%).

**Fig 3: Climate change perceptions and behavioral motivations among Puerto Rican farmers.**

## Puerto Rican farmers' psychological distance of climate change

We found that Puerto Rican farmers understand climate change as both a local and global phenomenon, being both psychologically close and psychologically distant across various measures. While we find evidence across all four dimensions of psychological distance for perceived "closeness", we also find that farmers demonstrate awareness for climate change effects beyond Puerto Rico in all dimensions (psychologically far) (Fig 4).

**Fig 4: Results for variables of the psychological distance of climate change.**

Respondents demonstrated a temporally close relationship to climate change with the majority disagreeing and strongly disagreeing (95.1%) that the effects of climate change are not being felt today. Regarding the spatial dimension, Puerto Rican farmers perceive that climate change will both negatively impact agriculture at both local and global scales (Fig 4). The majority disagreed and strongly disagreed (89.4%) with the statement, "climate change does not present more risks than benefits to agriculture in Puerto Rico" (e.g. Puerto Rico's agriculture is at risk). Farmers also agreed and strongly agreed (90.0%) with the statement focused on the global scale, "Climate change presents more risks than benefits to agriculture globally". At the social dimension, farmers expressed that climate change will both affect farmers (people like them), and the general public (people not like them). The majority (92%) disagreed and strongly disagreed that farmers like them will likely be affected negatively by climate change (psychologically close); 89% agreed and strongly agreed with the statement, "people who aren't farmers are likely to be negatively affected by climate change" (psychologically far).

We found the greatest variability in the uncertainty or hypothetical dimension of psychological distance of climate change. Thirty nine percent (39%) of respondents agreed and strongly agreed that there is scientific uncertainty about the potential impacts of climate change on Puerto Rico, 14% were neutral, while 47% disagreed and strongly disagreed with the statement. The majority (60%) disagreed and strongly disagreed that there is scientific uncertainty about the causes of climate change; 16% were neutral, and 24% agreed and strongly agreed with the statement.

Regarding the statement related to feeling uncertain about the relationship between climate change and occurrence of strong hurricanes in the Atlantic, 25% agreed and strongly agreed, 13% were neutral, and 63% disagreed and strongly disagreed. Furthermore, respondents overwhelmingly agreed that human activities are an important cause of climate change (3.2% disagreed and strongly disagreed, 5.4% were neutral, and 91.4% agreed and strongly agreed).

# Structural equation model

The structural equation model (S2 table, S3 table, S4 table, and fig 5) demonstrates that four of the seven hypothesized pathways were statistically significant ($p < 0.05$); S1 table shows results for all variables, including control variables. Goodness-of-fit criteria suggests the model is acceptable: RMSEA = 0.059, CFI = 0.861, SRMR = 0.058. Furthermore, the model showed that for motivation to adapt, our main dependent variable, $R^2 = 0.410$.

**Figure 5: Structural equation model results for the hypothesized model.** Thick arrows show significance ($p < 0.05$). Standardize coefficients are also shown (*b*). The model controls for farmer and farm characteristics, including age, gender, household income, farm size, education, and bonafide. Goodness-of-fit criteria suggest an overall good fit: RMSEA = 0.059, CFI = 0.861, SRMR = 0.058. $R^2$ for motivation to adapt was 0.410. Results of indirect effects and total effects are in S2 table and S3 table, respectively.

The model shows that reported hurricane damages and reported past experience with similar extreme events to Maria are not significantly predictive of the psychological distance of climate change amongst Puerto Rican farmers, a rejection of H1. Moreover, none of the control variables

(age, gender, farm size, bonafide, education, and income) were linked to the psychological distance of climate change (S2 table). However, both perceived capacity and vulnerability are significantly negatively related to the psychological distance of climate change ($p < 0.05$) (H2). In other words, farmers that perceived climate change as more distant were more likely to report lower rates of perceived capacity ($b = -0.140$, $p = 0.017$), and were less likely to report higher rates of perceiving their farms as vulnerable to future extreme weather events ($b = -0.164$, $p = 0.011$). We find no significant direct relationship between psychological distance of climate change and motivation to adapt; however, both perceived capacity ($b = 0.578$, $p = 0.000$) and perceived vulnerability ($b = 0.256$, $p = 0.000$) were positively linked to motivation to adapt. We find no significant relationships between control variables and reported past experience with similar extreme events (S2 Table). Regarding reported hurricane damages, farmers who are participants of the bonafide program of the Puerto Rico Department of Agriculture were more likely ($b =0.134$, $p = 0.034$) to report higher rates of damages to their farms due to Hurricane Maria. Furthermore, education level was negatively linked to reported damages by Hurricane Maria ($b = -0.177$, $p = 0.002$), meaning that increasing levels of formal education were linked to reporting lower rates of damages.

# Discussion

This study examined Puerto Rican farmers' psychological distance of climate change, and its relationship to reported damages following Hurricane Maria and reported experience with past extreme weather events, in order to better understand potential drivers and barriers to strengthen

adaptive capacity. We expand the current literature to utilize psychological distance of climate change beyond continental and high-income countries, and to understand how farmers' psychological distance of climate change relates to other cognitive beliefs in a vulnerable island-archipelago setting. We found that Puerto Rican farmers recognize climate change as a local and global issue (fig 4). Hence, they are neither psychologically close nor far, but psychologically aware of climate change's impacts across multiple time frames, geographies, and social constructs. We also found that neither reported experience with past extreme weather events, nor the reported damages effected by Maria were linked to farmers' psychological distance of climate change. Furthermore, farmers overwhelmingly perceived themselves self-capable and motivated to adapt to climate change. Nevertheless, motivation was not directly linked to their psychological distance of climate change.

Studies have used psychological distance to climate change to better understand whether this framing can prompt individual mitigation or adaptation actions. Moreover, the trend in the literature is that reduced psychological distance—mainly through experience with extreme weather events—will correlate with action [24, 30–32, 35, 37, 51]. Our results provide a different picture—we found that Puerto Rican farmers cannot be characterized as psychologically close or psychologically distant to climate change, but are psychologically aware of the impacts related to climate change at different scales. Furthermore, our model showed that variables assessing hurricane damage and reported experience with similar extreme weather events may not be the principal link to explain farmers' psychological distance to climate change (Fig 5). As well, psychological distance did not have a direct effect on motivation to adapt, but it did have an indirect effect on it through perceived self-capacity and vulnerability (S3 table and S4 table).

These results show that Puerto Rican farmers recognize climate change as being both near and far, which indicates that in the Caribbean context, where climate change is recognized as happening [61,62], a psychological distance framing may not be relevant. Our results on overall climate change perceptions, and farmers' psychological distance of climate change, align with a study by the Puerto Rican Department of Natural Resources that state that Puerto Ricans believe in, are aware of, and are concerned about climate change [63]. Puerto Rican farmers, overwhelmingly, understand that the climate is changing. This awareness of climate change as a threat is strong in Latin America and the Caribbean [61,62], where farmers and non-farmers are aware of the impacts related to climate change [62, 64, 65]. Surveyed farmers understand that climate change is happening now (temporal), that it will affect farmers and non-farmers (social), and that it will affect local and global agriculture (spatial). These results are in contrast to some existing research from the mainland of the U.S., where climate change belief among farmers is not nearly as high, especially in the perception of its anthropogenic causes [e.g.66,67]. Conversely, these results are more aligned with farmer perceptions of climate change from low-income countries, where climate change belief is overwhelmingly high [e.g. 68,69]. These results could suggest that when a threshold for climate change belief is passed (potentially achieved by continued exposure to extreme events or a collective social understanding), individuals recognize it as a problem that goes beyond their own self-interest. Furthermore, it could be that there is an understanding of the interactions (e.g. feedback loops) and interconnectedness amongst scales in and beyond a place, and indicates that there may be a saturation point for the psychological distance of climate change framing.  In other words, when enough people recognize climate change as a problem, both locally and globally, it may no longer catalyze action for climate mitigation or adaptation—other barriers may exist. This should be further studied.

Our respondents were heavily affected by Hurricane María; 42.6% reported total loss, while 45.6% reported significant losses. Given the absolute prevalence of damage across our population, these outcomes may relate to the fact that we find no relationship of reported damage to psychological distance. It is important to underscore that the last hurricane to directly impact Puerto Rico before Maria was 1998's Hurricane Georges. Moreover, Puerto Rico, as well as the Caribbean, have experienced concurrent hazards and been subject to non-landing hurricanes [4,70,71]. And given the age span of our participants, assuming they have lived in Puerto Rico, we can say that they have experienced over 50 tropical storms and hurricanes [70,71]. As such, these results further lend evidence to the idea of a "extreme events" threshold , whereby a certain frequency and intensity of extreme events no longer impacts climate change belief or psychological distance. . Nevertheless, "experience" in this study reported actual damage by the hurricane, as well as reporting experiencing similar events to Maria in the past 10 years. Given that experience has been measured in different ways, resulting in varied results, a future study should inquire deeply about what it means to "experience" an extreme event [72,73]. Furthermore, looking at experience and climate change perceptions from a qualitative perspective could also elicit a broader understanding about their linkage to experience and adaptation [74].

Our structural equation model showed that farmers who are bonafide participants in the Puerto Rico Department of Agriculture (52.8%) were more likely to report higher rates of damages ($R^2$ = .050). To participate in that program, 50% or more of farmers' income must come from agriculture. Farmers who are bonafide are exempted from some state and municipal taxes, and have access to other agricultural incentives. In our study, those that were bonafide had an average farm size of 85 *cuerdas*, and 76% of their income came from agriculture. This could

point to the relationship of structural determinants' (e.g. policy structures) to climate change's effects on farmers. We did not have variables looking at type of agricultural incentives, or access to governmental, community-based resources or assets; future studies could look at how policy structures, coupled with federal laws and Puerto Rico's context as a US territory, impact farmers' adaptive capacity. Furthermore, another line of research could focus on the extent to which dependence on Puerto Rico Department of Agriculture incentives relate to on-farm management and farm systems. This is timely given that Puerto Rico's recent incentives code, Law 60 of 2019, changed various regulations that have been in place for over 30 years [75]. Furthermore, given that farmers overwhelmingly reported feeling self-capable and motivated to adapt—variables that have been shown positively linked to decision-making around climate change adaptation [25]—future research and practitioners could focus on actual adaptation of agricultural practices (beyond intentions and cognitive factors), and those drivers and barriers at broader levels (e.g. community, institutional, regional, national) to strengthen adaptive capacity. The structural equation model (Fig 5) shows that perceiving climate change to be psychologically distant is linked to reporting lower rates of perceived self-capacity. The same was shown for perceived vulnerability to extreme weather events; the more distant climate change is perceived, the less vulnerable farmers perceived their farms to be to the impacts of future extreme weather events.. Nevertheless, farmers' psychological distance of climate change (and other variables in the model) does not explain much of the variability in both perceived self-capacity ($R^2$ = 0.019) and vulnerability ($R^2$ = 0.026). This further suggests that in areas constantly affected by extreme weather events, other variables beyond the ones explored here may be more relevant in explaining and fostering adaptation. Farmers in Latin America, the Caribbean, and Africa have expressed concern for climate change at different scales, but such

perceptions are poorly linked to actual climate change adaptation [38, 64, 65, 76]. Puerto Rican farmers overwhelmingly feel motivated to adapt to climate change, and that is positively linked to perceived self-capacity and perceived vulnerability. Nonetheless, taking into account the damage caused by Hurricane María and its aftermath, as well as the poor subsequent recovery efforts, our findings suggest that to strengthen farmers' adaptive capacity we must look at other determinants beyond the individual level.

In small island developing states and territories, where climate change impacts are already being felt to a greater extent than in high-income continental countries, cognitive beliefs nor psychological distance to climate change may be strong components of adaptive capacity. Farmers in Latin America [64,65], and the Caribbean [76,77] have been noted to lack institutional structures of support. In Puerto Rico, Perfecto and colleagues (2019) found that social capital and support networks were pivotal for Puerto Rican coffee farmers' recovery after Hurricane María, and that their agroecological practices, such as agroforestry, and other management styles were not sufficient for farms to be resilient and resistant within the catastrophic context of Hurricane Maria [78]. Hurricane María affected all of Puerto Rico, causing 2,975 deaths [79], and decimated the archipelago's agriculture. As such, we argue that reasons and circumstances beyond the self (cognitive beliefs) deserve more attention to better understand Puerto Rican farmers' decision-making, and increase their adaptive capacity. This conclusion aligns with a recent review by Wilson and colleagues (2020), which showed that studies on adaptation have taken two roads: one that seeks to understand the role of social and cognitive variables in adaptation behaviors, and another on how structural factors enact adaptation [80]. It is important to fill in the research gap on how societal and governance structures interact with social and cognitive beliefs in eliciting adaptation behaviors [80].

Taken results together, our study shows that the psychological distance of climate change may not be an appropriate framework to understand cognitive beliefs' role in adaptive capacity, especially in island settings, where climate change beliefs are overwhelmingly recognized.. Outside of island contexts, growing public sentiment that climate change is widely accepted as an important phenomenon is growing. While farmers in island and disadvantaged areas, who are at the forefront of constant climate change-related impacts, may have already reached a climate change belief saturation, it could be forthcoming in other regions and types of people. These results suggest that, if such things occur, climate change belief will likely not be the most important driver of climate change adaptation. Instead, structural, political, and other non-individual barriers to adaptation may be increasingly important. In the case of Caribbean farmers, they face institutional barriers to better adapt to climate change [14, 15, 45, 47, 81], and are localized in a region that still today is subject to colonial relationships and power dynamics that increase their vulnerability to natural hazards [19–21]. It is important to understand the degree to which psychological awareness (not distance) of climate change relates to other cognitive beliefs and decision-making, but this should co-exist with further efforts to more clearly examine non-individual barriers to adaptation.. Climate change adaptation cannot be reduced to something that is dependent on the individual because then we could be contributing on the assertion that the genesis of vulnerability lies on the individual [82]. Vulnerability to natural hazards is a systemic issue, and farmers' adaptation to climate change, as a decision-making process, is subject to structural interactions that cannot be overlooked.

# Limitations

We note some limitations in this study. First, to the best of our knowledge, there is no previous study looking at Puerto Rican or Caribbean farmers' psychological distance of climate change before and after an extreme weather event. We lack information on the issue before Hurricane Maria or other events. This limits our ability to understand if their psychological awareness is driven by experience. Nonetheless, Extension Service's internal data before Hurricane Maria showed that farmers were aware of climate change's impacts at different scales. Second, farmers surveyed showed higher percentage of income from farming than that reported in census data [53], and we had an overrepresentation of bonafide farmers. Nevertheless, most of the demographic factors of surveyed farmers here align with those of the latest agricultural census for Puerto Rico (table 3). Third, the variables we used to measure experience might not have capture a nuanced understanding of experience with extreme weather events [75]. Nonetheless, we use variables that have been used in past research regarding the psychological distance of climate change. Furthermore, given that most farmers surveyed for this study receive information from, or are linked to the Extension Service, this might suggest that they have higher access to information and resources, which may affect their understanding of climate change and other related issues.  As research in island systems on this topic continues to grow, there is opportunity for broader engagement with farmers, including those not directly integrated with the Extension Service.

# Conclusion

Farmers' adaptive capacity must be strengthened to decrease their vulnerability to natural hazards. In order to do so, cognitive beliefs should be considered given that climate change adaptation—a set of decisions at the individual level—is one key action to increase adaptive capacity. Here we assessed Puerto Rican farmers' climate change adaptation perceptions after Hurricane Maria to understand the role of experience with extreme events on the psychological distance of climate change, and its relation to motivation to adapt. We found that Puerto Rican farmers are psychologically aware of climate change impacts at different levels—local to global. These findings contrast with research in continental and high-income countries that seeks to reduce psychological distance to climate change amongst individuals to prompt mitigation and adaptation actions. Furthermore, we expand literature cemented on the assumption that being psychologically close enacts change, and that such psychological distance is driven by experience. We showed that neither reported experience with extreme events, nor farmers' reported damages by Hurricane Maria were determinants of farmers' psychological distance of climate change. Furthermore, here we demonstrate that context matters, and that the adaptive capacity of individuals embedded in vulnerable contexts—farmers in an island disproportionately exposed and sensitive to climate change's impacts—is not highly dependable on individual actions. We suggest that variables, such as institutional frameworks of support, must be better understood. Furthermore, research on the psychological distance of climate change should consider that we may have reached a threshold where climate change perceptions are not significant drivers of change. Hence, we suggest that understanding peoples' "psychological awareness of climate change" and its relation to context could provide a more nuance comprehension of climate change adaptation than focusing on how being "close" or "far" enacts change.

# Acknowledgements

We acknowledge all the Puerto Rican farmers that participated in this project, and the Extension Service's agricultural agents who enumerated the surveys. We are grateful to Dr. Aníbal Ruiz-Lugo, dean of the Extension Service, for leading the logistics and efforts on-site to carry out this project. Much thanks to Maritzabel Morales and the administrative team of the Extension Service for scanning all the surveys and assisting with administrative logistics, and to Olivia Peña for assisting in data entry. We are also thankful to Drs. María del Carmen Rodríguez-Rodríguez, Robinson Rodríguez-Pérez, and Fernando Pérez-Muñoz for assisting in the completion of the project. We are grateful to Dr. Jim Vigoreaux, Agro. Kiria Hurtado, and Carmen González-Toro for proofreading the Spanish version of the survey. We also acknowledge the colleagues who read the first draft of this paper, and provide us with meaningful review comments. Specially, we thank Amy Trubek, Teresa Mares, Maya Moore, Carolyn Hricko, and Caitlin B. Morgan. Much thanks as well to the University of Vermont's Food Systems Graduate Program and College of Agriculture and Life Sciences for providing funding in a timely manner to complete this research.

# References

1. Adger WN. Vulnerability. Global Environmental Change. 2006 Aug;16(3):268–81.

2. Wisner B, Gaillard J, Kelman I. Framing disaster: Theories and stories seeking to understand hazards, vulnerability and risk. In: The Routledge Handbook of Hazards and Disaster Risk Reduction. 1st ed. London; New York: Routledge; 2012. p. 18–33.
3. López-Marrero T. An integrative approach to study and promote natural hazards adaptive capacity: a case study of two flood-prone communities in Puerto Rico: An integrative approach to study and promote natural hazards adaptive capacity. Geographical Journal. 2010 Feb 25;176(2):150–63.
4. López-Marrero T, Wisner B. Not in the Same Boat: Disasters and Differential Vulnerability in the Insular Caribbean. Caribbean Studies. 2012;40(2):129–68.
5. Adger WN, Vincent K. Uncertainty in adaptive capacity. Comptes Rendus Geoscience. 2005 Mar;337(4):399–410.
6. Smit B, Wandel J. Adaptation, adaptive capacity and vulnerability. Global Environmental Change. 2006 Aug;16(3):282–92.
7. Brooks N, Adger WN. Assessing and Enhancing Adaptive Capacity. In: Adaptation Policy Frameworks for Climate Change: Developing Strategies, Policies and Measures. Cambridge, UK: Cambridge University Press; 2005. p. 165–82.
8. Wisner B, Blaikie P, Cannon T, Davis I. At Risk: Natural hazards, people’s vulnerability and disasters. 2nd ed. New York and London: Routledge; 2004.
9. Holt-Giménez E. Measuring farmers’ agroecological resistance after Hurricane Mitch in Nicaragua: a case study in participatory, sustainable land management impact monitoring. Agriculture, Ecosystems & Environment. 2002 Dec;93(1–3):87–105.
10. Shinbrot XA, Jones KW, Rivera-Castañeda A, López-Báez W, Ojima DS. Smallholder Farmer Adoption of Climate-Related Adaptation Strategies: The Importance of

Vulnerability Context, Livelihood Assets, and Climate Perceptions. Environmental Management. 2019 May;63(5):583–95.

11. Adger WN. Social Capital, Collective Action, and Adaptation to Climate Change. Economic Geography. 2003;79(4):387–404.

12. Fernandez M, Méndez VE. Subsistence under the canopy: Agrobiodiversity's contributions to food and nutrition security amongst coffee communities in Chiapas, Mexico. Agroecology and Sustainable Food Systems. 2019 May 28;43(5):579–601.

13. Niles MT, Mueller ND. Farmer perceptions of climate change: Associations with observed temperature and precipitation trends, irrigation, and climate beliefs. Global Environmental Change. 2016;39:133–42.

14. Álvarez-Berríos NL, Soto-Bayó S, Holupchinski E, Fain SJ, Gould WA. Correlating drought conservation practices and drought vulnerability in a tropical agricultural system. Renewable Agriculture and Food Systems. 2018 Jun;33(3):279–91.

15. Lowitt K, Hickey GM, Saint Ville A, Raeburn K, Thompson-Colón T, Laszlo S, et al. Factors affecting the innovation potential of smallholder farmers in the Caribbean Community. Regional Environmental Change. 2015 Oct;15(7):1367–77.

16. Rosset PM, Sosa BM, Jaime AMR, Lozano DRÁ. The Campesino-to-Campesino agroecology movement of ANAP in Cuba: Social process methodology in the construction of sustainable peasant agriculture and food sovereignty. Journal of Peasant Studies. 2011;38(1):161–91.

17. Saint Ville AS, Hickey GM, Phillip LE. Institutional analysis of food and agriculture policy in the Caribbean: The case of Saint Lucia. 2017 [cited 2018 Aug 15]; Available from: http://dx.doi.org/10.1016/j.jrurstud.2017.03.004

18. Scandurra G, Romano AA, Ronghi M, Carfora A. On the vulnerability of Small Island Developing States: A dynamic analysis. Ecological Indicators. 2017;84:382–92.
19. Borges-Méndez R, Caron C. Decolonizing Resilience: The Case of Reconstructing the Coffee Region of Puerto Rico After Hurricanes Irma and Maria. Journal of Extreme Events. 2019 Mar;06(01):1940001.
20. Félix G, Holt-Giménez E. Hurricane María: An Agroecological Turning Point for Puerto Rico? Food First Backgrounder. 2017;23(4).
21. Moulton AA, Machado MR. Bouncing Forward After Irma and Maria: Acknowledging Colonialism, Problematizing Resilience and Thinking Climate Justice. Journal of Extreme Events. 2019 Mar;06(01):1940003.
22. Grothmann T, Patt A. Adaptive capacity and human cognition: The process of individual adaptation to climate change. Global Environmental Change. 2005 Oct;15(3):199–213.
23. Ostrom E. Polycentric systems for coping with collective action and global environmental change. Global Environmental Change. 2010 Oct;20(4):550–7.
24. Niles MT, Lubell M, Brown M. How limiting factors drive agricultural adaptation to climate change. Agriculture, Ecosystems and Environment. 2015;200:178–85.
25. van Valkengoed AM, Steg L. Meta-analyses of factors motivating climate change adaptation behaviour. Nature Climate Change. 2019 Feb;9(2):158–63.
26. Gebrehiwot T, van der Veen A. Farmers Prone to Drought Risk: Why Some Farmers Undertake Farm-Level Risk-Reduction Measures While Others Not? Environmental Management. 2015 Mar;55(3):588–602.

27. Bagagnan A, Ouedraogo I, M. Fonta W, Sowe M, Wallis A. Can Protection Motivation Theory Explain Farmers' Adaptation to Climate Change Decision Making in The Gambia? Climate. 2019 Jan 14;7(1):13.

28. Luu TA, Nguyen AT, Trinh QA, Pham VT, Le BB, Nguyen DT, et al. Farmers' Intention to Climate Change Adaptation in Agriculture in the Red River Delta Biosphere Reserve (Vietnam): A Combination of Structural Equation Modeling (SEM) and Protection Motivation Theory (PMT). Sustainability. 2019 May 27;11(10):2993.

29. Spence A, Poortinga W, Butler C, Pidgeon NF. Perceptions of climate change and willingness to save energy related to flood experience. Nature Climate Change. 2011;1(1):46–9.

30. Brügger A. Understanding the psychological distance of climate change: The limitations of construal level theory and suggestions for alternative theoretical perspectives. Global Environmental Change. 2020 Jan;60:102023.

31. McDonald RI, Chai HY, Newell BR. Personal experience and the "psychological distance" of climate change: An integrative review. Journal of Environmental Psychology. 2015;44:109–18.

32. Spence A, Poortinga W, Pidgeon N. The Psychological Distance of Climate Change. Risk Analysis. 2012;32(6):957–72.

33. Trope Y, Liberman N. Construal-Level Theory of Psychological Distance. Psychological Review. 2010;117(2):440–63.

34. Liberman N, Trope Y. The Psychology of Transcending the Here and Now. Vol. 322, Source: Science, New Series. 2008.

35. Brügger A, Morton TA, Dessai S. “Proximising” climate change reconsidered: A construal level theory perspective. Journal of Environmental Psychology. 2016;46:125–42.
36. Jones C, Hine DW, Marks ADG. The Future is Now: Reducing Psychological Distance to Increase Public Engagement with Climate Change. Risk Analysis. 2017;37(2):331–41.
37. Singh AS, Zwickle A, Bruskotter JT, Wilson R. The perceived psychological distance of climate change impacts and its influence on support for adaptation policy. Environmental Science & Policy. 2017;73:93–9.
38. Acharibasam JW, Anuga SW. Psychological distance of climate change and mental health risks assessment of smallholder farmers in Northern Ghana: Is habituation a threat to climate change? Climate Risk Management. 2018;21:16–25.
39. Larcom S, She P-W, van Gevelt T. The UK summer heatwave of 2018 and public concern over energy security. Nature Climate Change. 2019 May;9(5):370–3.
40. Albright EA, Crow D. Beliefs about climate change in the aftermath of extreme flooding. Climatic Change. 2019 Jul;155(1):1–17.
41. Zanocco C, Boudet H, Nilson R, Satein H, Whitley H, Flora J. Place, proximity, and perceived harm: extreme weather events and views about climate change. Climatic Change. 2018;149(3–4):349–65.
42. Bang HN, Miles LS, Gordon RD. Hurricane Occurrence and Seasonal Activity: An Analysis of the 2017 Atlantic Hurricane Season. American Journal of Climate Change. 2019;08(04):454–81.
43. Bueno R. Puerto Rico, Climatic Extremes, and the Economics of Resilience. Climate Policy Brief. 2017;(6).

44. Bueno R, Herzfeld C, Stanton E a., Ackerman F. The caribbean and climate change: The costs of inaction [Internet]. Stockholm Environment Institute and Global Development and Environment Institute, Tufts University; 2008 [cited 2018 Apr 12] p. 35. Available from: http://www.sei-us.org/climate-and-energy/Caribbean_Inaction_Cost.htm%5Cnhttp://www.gdae.org/CaribbeanClimate.html
45. Carro-Figueroa V. Agricultural Decline and Food Import Dependency in Puerto Rico: A Historical Perspective on the Outcomes of Postwar Farm and Food Policies. Caribbean Studies. 2002;30(2):77–107.
46. Gould WA. Caribbean Regional Climate Sub Hub Assessment of Climate Change Vulnerability and Adaptation and Mitigation Strategies. 2015;67.
47. Myrna Comas-Pagán. Plan de sguridad alimentaria. San Juan, Puerto Rico: Puerto Rico Department of Agriculture; 2014 p. 40.
48. Irizarry-Ruiz C. Desafíos de la agricultura en Puerto Rico para garantizar la seguridad alimentaria. In: Retos que conlleva la reutilización de aguas residuales en la agricultura. San Juan, Puerto Rico; 2016.
49. FAO. Food security and nutrition in small island developing states (SIDS). Policy Paper. 2014;16.
50. Weis T. Small farming and radical imaginations in the Caribbean today. Race & Class. 2007 Oct;49(2):112–7.
51. López-Marrero T, Castro-Rivera A. Actividad ciclónica en Puerto Rico y sus alrededores: 1867 al 2017. Mayagüez, Puerto Rico: Centro Interdisciplinario de Estudios del Litoral; 2018. 12 p.

52. Perfecto I, Hajian-Forooshani Z, Iverson A, Irizarry AD, Lugo-Perez J, Medina N, et al. Response of Coffee Farms to Hurricane Maria: Resistance and Resilience from an Extreme Climatic Event. Scientific Reports [Internet]. 2019 Dec [cited 2019 Nov 5];9(1). Available from: http://www.nature.com/articles/s41598-019-51416-1
53. Puerto Rico's Agriculture and Farmers Decimated by Maria - The New York Times [Internet]. [cited 2020 Sep 25]. Available from: https://www.nytimes.com/2017/09/24/us/puerto-rico-hurricane-maria-agriculture-.html
54. USDA NASS. 2017 Census of Agriculture - Puerto Rico Data [Internet]. 2020 [cited 2020 Jun 12] p. 117. Available from: https://www.nass.usda.gov/Publications/AgCensus/2017/index.php
55. Ray A, Hughes L, Konisky DM, Kaylor C. Extreme weather exposure and support for climate change adaptation. Global Environmental Change. 2017;46:104–13.
56. Haden VR, Niles MT, Lubell M, Perlman J, Jackson LE. Global and Local Concerns: What Attitudes and Beliefs Motivate Farmers to Mitigate and Adapt to Climate Change? PLoS ONE [Internet]. 2012 [cited 2018 Sep 3];7(12). Available from: www.plosone.org
57. Response Rates - An Overview - AAPOR [Internet]. [cited 2020 Sep 23]. Available from: https://www.aapor.org/Education-Resources/For-Researchers/Poll-Survey-FAQ/Response-Rates-An-Overview.aspx
58. Nunnally JC. Psychometric Theory. McGraw-Hill; 1978.
59. Iacobucci D. Everything you always wanted to know about SEM (structural equations modeling) but were afraid to ask. Journal of Consumer Psychology. 2009 Oct;19(4):673–80.

60. Schreiber JB, Nora A, Stage FK, Barlow EA, King J. Reporting Structural Equation Modeling and Confirmatory Factor Analysis Results: A Review. The Journal of Educational Research. 2006 Jul;99(6):323–38.

61. Tang J-W, Chen M-L, Chiu T-H. An Exploratory Study on Local Brand Value Development for Outlying Island Agriculture: Local Food System and Actor-Network Theory Perspectives. Sustainability. 2018 Nov;10(11):4186.

62. Lai K. Estimating Standardized SEM Parameters Given Nonnormal Data and Incorrect Model: Methods and Comparison. Structural Equation Modeling: A Multidisciplinary Journal. 2018 Jul 4;25(4):600–20.

63. USDA NASS. USDA Releases 2018 Puerto Rico Census of Agriculture [Internet]. 2020 [cited 2020 Jun 12]. Available from: https://www.nass.usda.gov/Newsroom/2020/06-09-2020.php

64. Díaz Rolón A. Aspira a que haya más agricultores bonafide [Internet]. El Vocero de Puerto Rico. 2019 [cited 2020 Jun 12]. Available from: https://www.elvocero.com/gobierno/aspira-a-que-haya-m-s-agricultores-bonafide/article_017eaa76-a2b8-11e9-9255-8773684782d5.html

65. Lee TM, Markowitz EM, Howe PD, Ko CY, Leiserowitz AA. Predictors of public climate change awareness and risk perception around the world. Nature Climate Change. 2015;5(11):1014–20.

66. Forero EL, Hernández YT, Zafra CA. Percepción latinoamericana de cambio climático: metodologías, herramientas y estrategias de adaptación en comunidades locales. Una revisión. Revista UDCA Actualidad & Divulgación Científica. 2014 Mar 10;17(1):73–85.

67. Departamento de Recursos Naturales de Puerto Rico. Estudio de percepción pública sobre el riesgo y la resiliencia al cambio climático [Internet]. San Juan, Puerto Rico: Puerto Rico Department of Natural Resources; 2018 Sep [cited 2020 Jun 13] p. 95. Available from: http://drna.pr.gov/wp-content/uploads/2019/01/Informe-final-Estudio-de-percepcion-publica-sobre-cambio-climatico.pdf
68. Viguera B, Alpizar F, Harvey CA, Martínez-Rodríguez MR, Saborío-Rodríguez M. Percepciones de cambio climático y respuestas adaptativas de caficultores costarricenses de pequeña escala. Agronomía Mesoamericana. 2019 May 1;333–51.
69. Viguera B, Alpizar F, Harvey CA, Martínez-Rodríguez MR, Saborío-Rodríguez M, Contreras L. Climate change perceptions and adaptive responses of small-scale farmers in two Guatemalan landscapes. Agronomía Mesoamericana. 2019;30(2):313'331.
70. Prokopy LS, Arbuckle JG, Barnes AP, Haden VR, Hogan A, Niles MT, et al. Farmers and Climate Change: A Cross-National Comparison of Beliefs and Risk Perceptions in High-Income Countries. Environmental Management. 2015 Aug;56(2):492–504.
71. Chatrchyan AM, Erlebacher RC, Chaopricha NT, Chan J, Tobin D, Allred SB. United States agricultural stakeholder views and decisions on climate change. WIREs: Climate Change. 2017 Oct 9;8(5):n/a-N.PAG.
72. Waldman KB, Attari SZ, Gower DB, Giroux SA, Caylor KK, Evans TP. The salience of climate change in farmer decision-making within smallholder semi-arid agroecosystems. Climatic Change. 2019 Oct 1;156(4):527–43.
73. Debela N, Mohammed C, Bridle K, Corkrey R, McNeil D. Perception of climate change and its impact by smallholders in pastoral/agropastoral systems of Borana, South Ethiopia. SpringerPlus. 2015 May 20;4(1):236.

74. López-Marrero T, Castro-Rivera A. Let’s not forget about non-land-falling cyclones: tendencies and impacts in Puerto Rico. Natural Hazards [Internet]. 2019 Aug 6 [cited 2019 Aug 22]; Available from: http://link.springer.com/10.1007/s11069-019-03716-y
75. Demuth JL. Explicating Experience: Development of a Valid Scale of Past Hazard Experience for Tornadoes. Risk Analysis. 2018;38(9):1921–43.
76. Demuth JL, Morss RE, Lazo JK, Trumbo C. The Effects of Past Hurricane Experiences on Evacuation Intentions through Risk Perception and Efficacy Beliefs: A Mediation Analysis. Weather, Climate, and Society. 2016 Oct 1;8(4):327–44.
77. Ranjan P, Church SP, Floress K, Prokopy LS. Synthesizing Conservation Motivations and Barriers: What Have We Learned from Qualitative Studies of Farmers’ Behaviors in the United States? Society & Natural Resources. 2019 Nov 2;32(11):1171–99.
78. Código de incentivos de Puerto Rico. P. de la C. 1635, 60 Jul 1, 2019.
79. Campbell, Donovan, Barker D, McGregor D. Dealing with drought: Small farmers and environmental hazards in southern St. Elizabeth, Jamaica. Applied Geography. 2011;31:146–58.
80. Stancioff C, Stojanov R, Kelman I, Němec D, Landa J, Tichy R, et al. Local Perceptions of Climate Change Impacts in St. Kitts (Caribbean Sea) and Malé, Maldives (Indian Ocean). Atmosphere. 2018 Nov 22;9(12):459.
81. Santos-Burgoa C. Acertainment of the Estimated Excess Mortality from Hurricane Maria in Puerto Rico.pdf [Internet]. 2018 [cited 2019 May 20]. Available from: https://publichealth.gwu.edu/sites/default/files/downloads/projects/PRstudy/Acertainment%20of%20the%20Estimated%20Excess%20Mortality%20from%20Hurricane%20Maria%20in%20Puerto%20Rico.pdf

82. Wilson RS, Herziger A, Hamilton M, Brooks JS. From incremental to transformative adaptation in individual responses to climate-exacerbated hazards. Nature Climate Change. 2020 Feb 10;1–9.

83. Graham B. Profile of the Small-Scale Farming in the Caribbean. Food and Agriculture Organization; 2012 p. 62.

84. Ribot J. Cause and response: vulnerability and climate in the Anthropocene. The Journal of Peasant Studies. 2014 Sep 3;41(5):667–705.

# Supporting information

**S1 Table. Exploratory factor analysis results for pilot study's assessment of the psychological distance of climate change scale**. Factor loadings are shown, as well as the Cronbach's alpha for statements altogether.

**S2 Table. Structural equation model structural results for our hypothesized model.** Standardized coefficients (β), bootstrap standard error (SE), and significance level (*p*) are included.

**S3 Table. Structural equation model structural results for indirect effects of our hypothesized model.** Standardized coefficients (β), bootstrap standard error (SE), and significance level (*p*) are included.

**S4 Table. Structural equation model structural results for total effects of our hypothesized model.** Standardized coefficients (β), bootstrap standard error (SE), and significance level (p) are included.

Fig 1_Hypothesized model

Click here to access/download;Figure;Fig1.tiff.tif

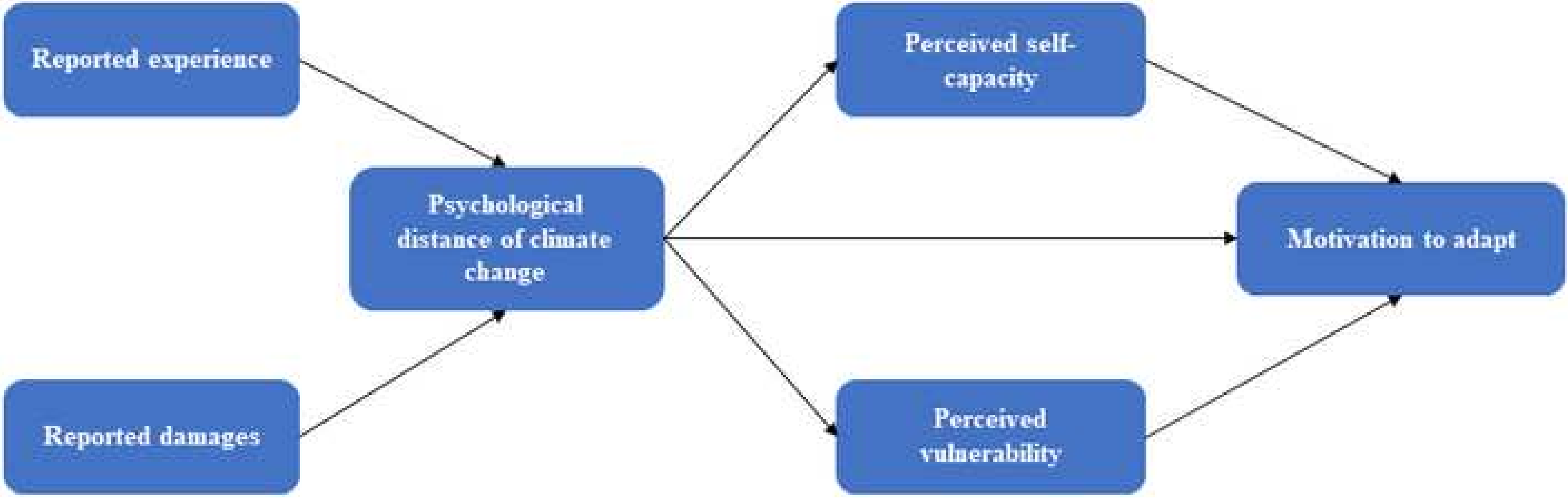

Click here to access/download;Figure;Fig 2.tiff.tif

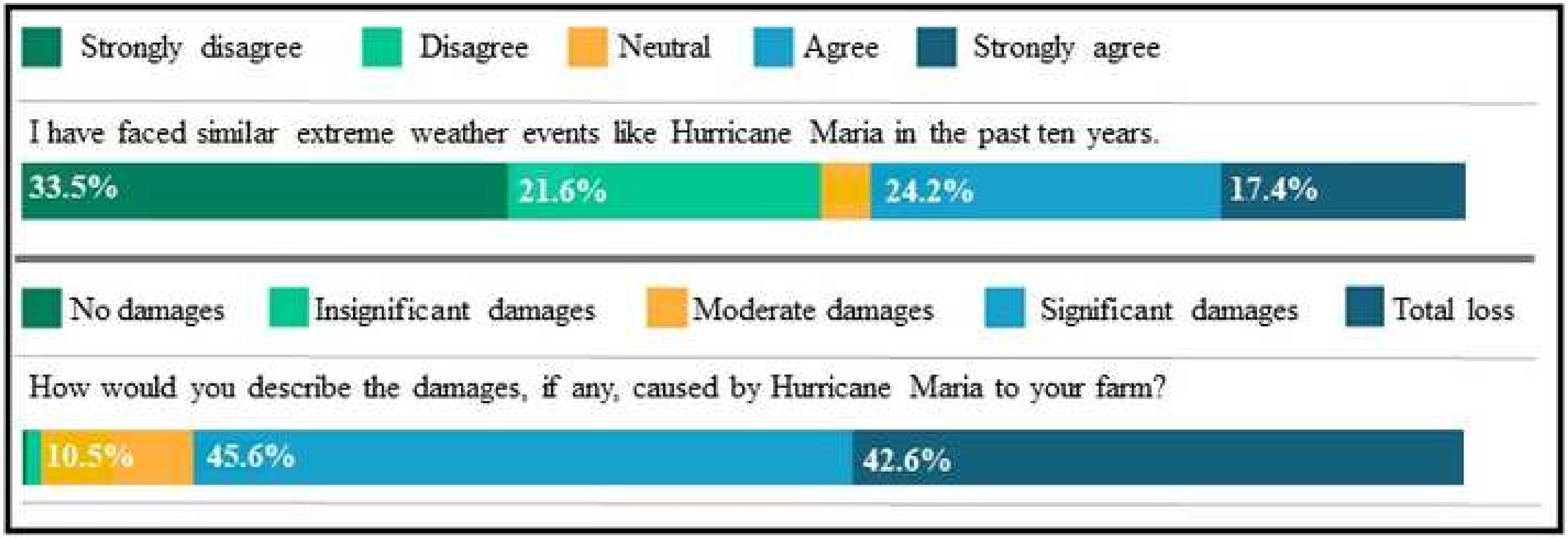

Fig 3_General climate change and adaptation perceptions

Click here to access/download;Figure;Fig 3.tiff.tif

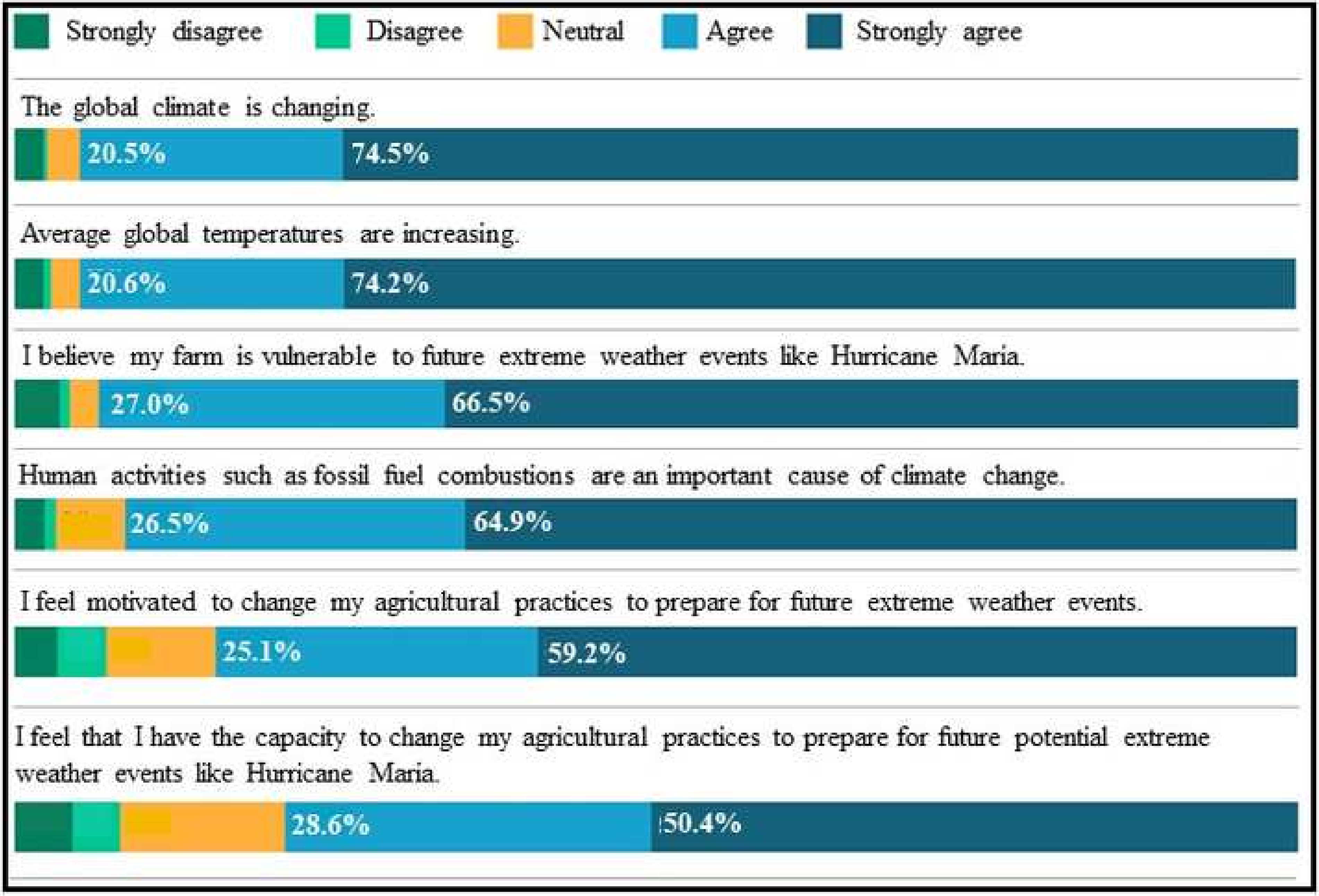

 Click here to access/download;Figure;Fig 5.tiff.tif

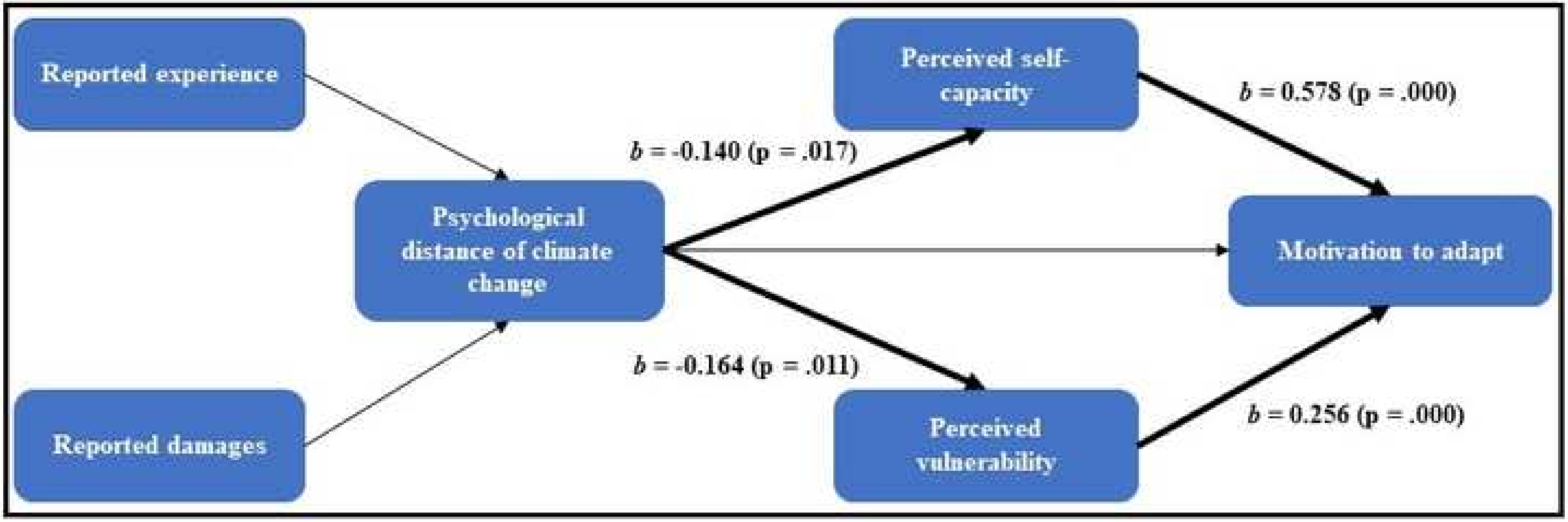

 Click here to access/download;Figure;Fig 4.tiff.tiff

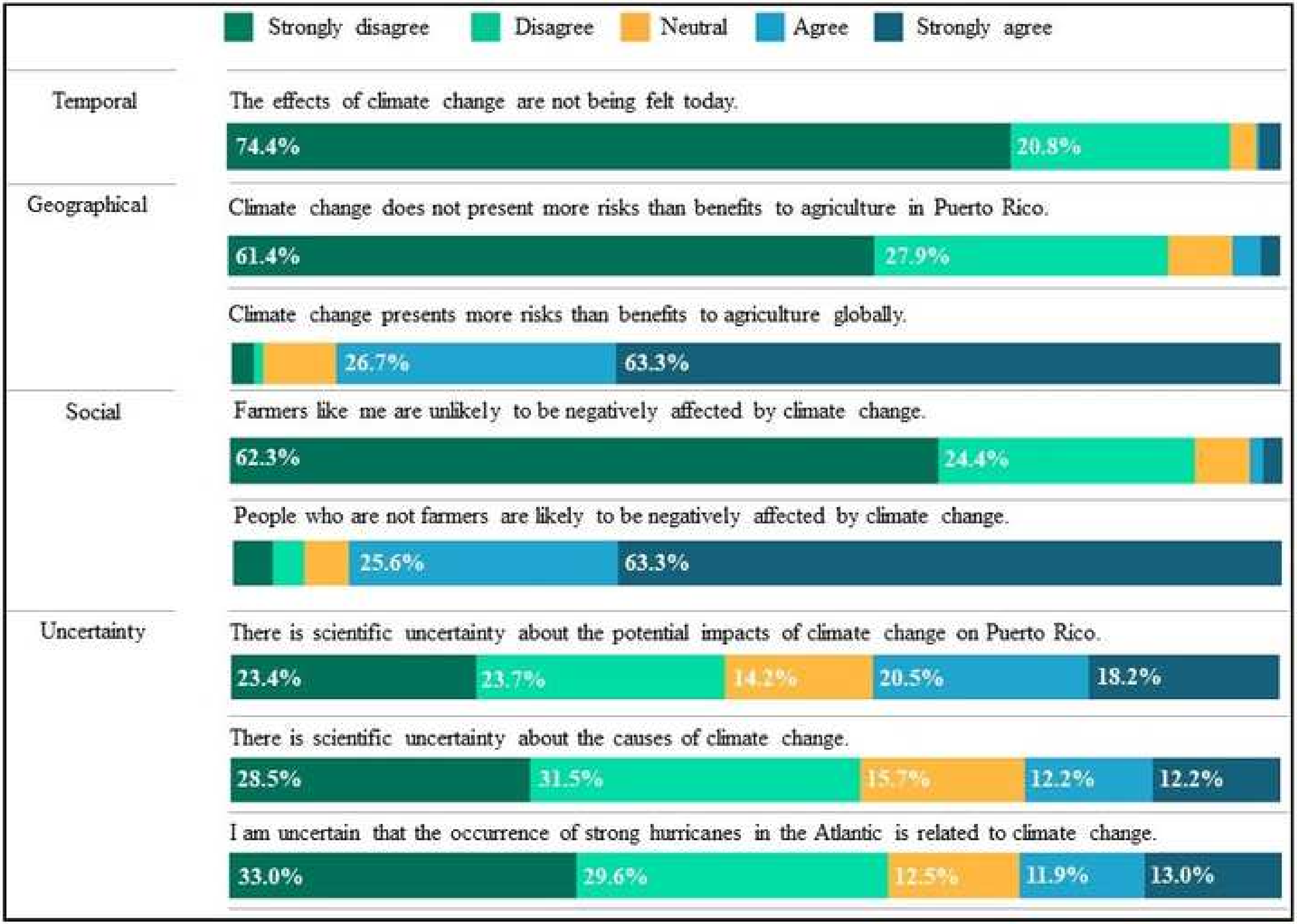

S1 Table

Click here to access/download
**Supporting Information**
S1_Table.docx

S2 Table

Click here to access/download
**Supporting Information**
S2_Table.docx

S3 Table

Click here to access/download
**Supporting Information**
S3_Table.docx

S4 Table

Click here to access/download
**Supporting Information**
S4_Table.docx

# Puerto Rican farmers' psychological awareness of climate change, and adaptation perceptions after Hurricane Maria

Luis Alexis Rodríguez-Cruz[1,3*] & Meredith T. Niles[2,3*]

[1]Food Systems Graduate Program, University of Vermont, Burlington, Vermont

[2]Department of Nutrition and Food Sciences and Food Systems Graduate Program, University of Vermont, Burlington, Vermont

[3]Gund Institute for the Environment, University of Vermont, Burlington, Vermont.

*Corresponding author

E-mail: ~~mtniles~~lrodrig2@uvm.edu (~~MTN~~LARC)

LARC contributions: conceptualized research, survey development, project liaison, data entry and analysis, wrote manuscript draft.

MTN contributions: Conceptualized research, survey development, funding acquisition, data analysis, edited manuscript draft.

# Abstract

Understanding how experience with extreme events are linked to climate change adaptation's social and cognitive drivers is key in strengthening adaptive capacity. Here we use the psychological distance of climate change as a framework to explore Puerto Rican farmers' adaptation perceptions after Hurricane Maria. Farmers were surveyed eight months after the hurricane by Extension agents (n = 405, 87% response rate). A structural equation model assessed how reported experience with past extreme events and hurricane damages related to their psychological distance of climate change, and how these variables were linked to their adaptation perceptions. Results showed that Puerto Rican farmers have a high level of climate change belief, and a psychological distance of climate change that is both near and far, since they show a broad awareness to climate change's impacts both locally and globally in different dimensions (temporal, spatial, and social). Further, we find that reported previous experience with similar extreme events to Maria, and intensity of hurricane damages caused by Maria were not related to their psychological distance of climate change, and these did not relate to their motivation to adapt. These results suggest that we may be reaching a tipping point for extreme events as a driver for climate belief and action, especially in a context where there is a high level of climate change belief and continued threat of extreme events. Instead, these results indicate that factors beyond individual perceptions and beliefs, such as structural, institutional or political factors, may be critical barriers to climate change adaptation. Thus, broadening analysis beyond individual perceptions as drivers of climate adaptation to how structural dynamics are linked to

adaptive capacity may provide better understanding of adaptation barriers. This study is amongst the first to explore farmers’ psychological distance of climate change in an island-archipelago setting after an extreme event.

Strengthening farmers’ adaptive capacity is important for decreasing their vulnerability to natural hazards in this changing climate, and for safeguarding local food systems. One key step in strengthening adaptive capacity is understanding the relationship between farmers’ experience with natural hazards and their perceptions of climate change, and its role in farmers’ decision making around climate change adaptation. Here we explore Puerto Rican farmers’ psychological distance of climate change after experiencing category 4 Hurricane Maria, in order to intersect the roles of experience, perceptions, and motivation for farmers’ decision-making around climate change adaptation. ’mayexperiences withextreme events climate change Farmers throughout Puerto Rico were surveyed by Extension Services’ agents (n = 405, 87% response rate) in 2018, eight months after Hurricane Maria. A structural equation model was used to evaluate how reported experience with past events and direct damages by Hurricane Maria related to farmers’ psychological distance of climate change, and the association of these variables to farmers’ motivation to adapt to climate change. We found that farmers have a high level of climate change belief, and a psychological distance of climate change is that is both near and far, since they show a broad awareness to climate change’s impacts both locally and globally in different dimensions (temporal, geographical, and social). Further, we find that previous experience with extreme events, and intensity of hurricane damages Reported experience and direct damages by the hurricane were not linked related to their psychological distance of climate change, and these did not relate to their motivation to adapt. These results suggest that we may be reaching a tipping point for using extreme events as a driver for climate belief and action, may no longer be

relevant, especially in a context where there is a high level of climate change belief and continued threat of extreme events. Instead, these results indicate that factors beyond individual perceptions and beliefs, such as structural, institutional or political factors, may be critical barriers to climate change adaptation. Thus, broadening analysis beyond individual perceptions as drivers of climate adaptation to how structural dynamics are linked to adaptive capacity could may provide better understanding of adaptation barriers. This study is among the first to study the role of climate change experience and perceptions on farmers' climate change adaption after an extreme weather event in an island archipelago setting.

# Introduction

Farmers across the globe are at the forefront of climate change-related impacts, and are thus important actors in safeguarding our food systems [1,2] . Natural hazards, such as hurricanes and droughts, have made farming more difficult due to an increased frequency, and, in many cases, negatively affected farmers' livelihoods [3,4]. This is notably true of those who farm in the tropics, especially farmers in island states and territories, who are more vulnerable to the impacts of natural hazards given islands' unique characteristics, such as small territories and economies, dependence on imports, and disproportionate exposure to sea-level rise and extreme weather events [5–8].

Exposure, sensitivity, and adaptive capacity are three main determinants of farmers' vulnerability to natural hazards [19,210]. Farmers in small island states and territories, most of which are located in the tropics, farm in regions that are often disproportionately exposed and sensitive to natural hazards [210–412]. Exposure refers to islands being situated in regions highly

prone to extreme weather events (e.g. Atlantic's 'Hurricane Belt'), and sensitivity relates to the degree to which such hazards change physical systems (e.g. farms) [19,412–614]. As such, strengthening adaptive capacity—defined as the set of abilities and resources individuals and populations have to anticipate, withstand, cope with, and recover from a hazard, and the potential or abilities they have to modify the system in order to be more resistant to impacts [311,715,816]—is key in decreasing vulnerability to natural hazards. ~~This paper utilizes the psychological distance of climate change as its framework to explore Puerto Rican farmers' experience with Hurricane Maria, and their perceptions of climate change adaptation to understand potential drivers and barriers for adopting behaviors that strengthen adaptive capacity. This framework, which has been primarily used in non-island settings, allow us to intersect experience, perceptions, and their association to adaptation [17–19]. Focusing on Puerto Rico fills a current gap on food systems research in small island states and territories focused on exposure and sensitivity to natural hazards. Furthermore, it gives us the ability to generate novel information for scholarship focused on Puerto Rico as well.~~

Adaptive capacity is complex, and involves many diverse and interrelated determinants [311,412]. In the case of farmers, the adoption of new adaptation technologies and practices, as well as the access to institutional resources and networks of support, are important for strengthening adaptive capacity [93,104,1120–1322]. Research in the Caribbean and Central America—areas exposed to Atlantic hurricanes—have shown that farms and farmers' characteristics, as well as their access to diverse markets and sources of support are important in reducing vulnerability to natural hazards [93,1423–1276]. Nevertheless, given the heterogeneity of ~~islands'~~ the regions' sociopolitical systems, context-specific research is important to better understand adaptive capacity [412,1827]. This is evident in the Caribbean, given the region's

neocolonial dynamics, where many islands' sovereignty resides on continental countries [19~~28–~~21~~30~~]. This is the case of Puerto Rico as an Unincorporated Territory of the United States. Studying Puerto Rican farmers' climate change adaptation could allow us to understand how ~~reflect~~ broader sociopolitical structures~~'~~ ~~roles~~ reflect in the process of strengthening adaptive capacity.

Understanding the role of perceptions and beliefs as determinants of adaptive capacity is key to determining effective strategies [~~(~~3,6,22] ~~)~~. As such, it is important to understand to what extent beliefs and perceptions ~~play a role~~are linked in farmers' decision-making processes around climate change adaptation, which reflects adaptive capacity ~~(5,6,11)~~[5,6,11]. Climate change adaptation occurs at different scales [11~~20~~,23~~32~~], and it is contextualized, highlighting that understanding the degree to which cognitive beliefs impact individuals' decision-making and willingness to act is important in assessing adaptive capacity [5~~13~~,24~~17~~,22~~31~~,25~~33~~]. Studies have found that individuals' perceived capacity, perceived vulnerability, motivation to adapt, and other social and cognitive variables are key in understanding adaptation behaviors ~~(10,22,24,25)~~[10,22,24,25]. The ways farmers perceived their capacity to adopt new adaptation behaviors, and the risks they are exposed to have been shown to drive the adoption of new agricultural practices and pro-environmental behaviors ~~(10,24,26–28)~~[10,24,26–28]. While existing research~~-~~ demonstrates a link between climate perceptions, ~~and~~ beliefs, and climate change adaptation amongst the general public [29~~34~~], and farmers [24~~17~~], there are varied results [25~~33~~,30~~5~~,31~~6~~]. Importantly, most research focused on perceptions about climate and the role of cognitive beliefs in climate adaptation is from high-income countries [25~~33~~]. There is limited evidence of this topic from small island developing states and territories, whose unique exposure

and sensitivity to climate change and extreme events may influence how residents perceive climate change and their adaptive capacity.

To contribute to fill the current gaps in the literature, we examine survey results from 405 Puerto Rican farmers following Hurricane Maria, a category four hurricane, which impacted Puerto Rico in September 2017. We examine farmers' perceptions and beliefs about climate change, its potential link~~s~~ to their reported damages from~~experience~~ ~~with~~ the hurricane as a proxy for direct experience, their reported past experience with similar events, and ~~as well as~~ the extent to which these perceptions and beliefs influence their motivation to adapt to climate change. We use~~ing~~ the theoretical framework of the psychological distance of climate change—simply put, how near or far people perceive climate change to be from themselves in various contexts [29~~19~~,32~~4~~,33~~7~~,34~~8~~]. We intend to evaluate the degree to which the psychological distance of climate change amongst Puerto Rican farmers manifests after the brunt event of a hurricane. This baseline work can expand our understanding of the role of cognitive beliefs and perceived psychological distance for prompting adaptation behaviors to strengthen adaptive capacity amongst individuals beyond high-income and continental countries. Furthermore, it gives us the ability to generate novel information for scholarship focused on farmers' adaptive capacity in Puerto Rico as well.

## Theoretical framework

Construal Level Theory states that humans imagine the past and future through abstract constructs, since we are embedded in the here and now [33~~7~~,34~~8~~]. Psychological distance, a

component of the aforementioned theory, is a subjective experience that uses the self as a reference point [33~~7~~,34~~8~~]. Psychological distance is measured in four components: temporal, social, ~~geographical~~spatial, and hypothetical (uncertainty of the distance or issue). The more distant~~,~~ we perceive an issue or event to be across these dimensions, the more we rely on abstract constructs to make sense of it. In contrast, things we perceive as closer are more concrete. For example, as an event gets closer in time (e.g. conference presentation), the awareness of the dynamics involved become more concrete (e.g. transportation to the venue, nervousness, time management, and other variables). Thus, Construal Level Theory explains that we use mental constructs to build understanding of what transcends the here and now.

Applied in the realm of climate change communication and adaptation research, the psychological distance of climate change [32~~19~~] refers to how near or far people (e.g. farmers) perceive climate change to be from themselves across the four dimensions: temporal (when climate change will occur or if is occurring), ~~geographical~~ spatial (where it happens), social (to whom it happens), and the hypothetical or uncertainty dimension (certainty of whether it occurs or not). Theoretically, this posits that individuals who are psychologically close to climate change are prone to perceive higher risks to hazards, and a greater motivation to act to adapt or mitigate climate change. Nonetheless, this assumption has not been consistent in research [30~~5~~,31~~6~~].

## Research on the psychological distance of climate change

The aforementioned hypothesis has been the basis for many existing studies on the psychological distance of climate change (e.g. understanding how psychological distance affects the propensity for individuals to take action to mitigate or adapt to climate change). The current body of research focused on the psychological distance of climate change is both experimental [359,3640], and observational [324,3741)]. More specifically, scholarship has explored (1) the interrelatedness of the different dimensions, (2) the role of experiencing extreme weather events on individuals' psychological distance of climate change, and (3) the degree to which psychological distance of climate change affects behavior. Overall, evidence indicates inconsistent outcomes related to the psychological distance of climate change and its capacity to prompt action, as well as the understanding of how experiences with extreme events affects psychological distance, and the relatedness of the four dimensions varies [316]. These results may suggest that context matters; a natural hazard (e.g. hurricane), for example, can be experienced and perceived differently depending on context (e.g. sociodemographic characteristics, beliefs, adaptive capacity) [19,210].

Some studies support that the four dimensions of the psychological distance of climate change are interrelated. Moreover, studies suggest that being psychologically close impacts behavior. For example, Spence and colleagues (2011, 2012) demonstrated that reported experience with floods was linked to individuals' perceptions of climate change in the United Kingdom. Their studies showed that the four dimensions of the psychological distance of climate change were interrelated, and that reduced psychological distance related to promptness prompting to climate change mitigation actions amongst individuals [3219,2934]. Similarly, but based in the United States, Singh et al. (2017) found that the four dimensions were interrelated, and that those psychologically close expressed more support towards climate change adaptation policies. In that

study, psychological distance was linked to experience with extreme events. Tthe temporal and uncertainty dimensions, by their own, did not have significant effect on policy support; however, those that expressed higher uncertainty over climate change and were socially distant, expressed less support towards policies [3741]. Furthermore, research has shown that the dimensions, though often interrelated, depending on context, manifest differently [316]. Focused on New Zealand's farmers, Niles et al. (2015) applied the psychological distance of climate change framework in combination with Liebig's Law of the Minimum to assess adaptation behaviors, finding that decreasing psychological distance may prompt action from farmers. Farmers who perceived climate change impacts as geographically spatially close, were more likely to feel more concern for climate change and motivation to adapt [2417]. Research has also described how experience with extreme weather events relates to being psychologically close [2417,2934,316,3741]. Acharibasam and Anuga (2018) found that farmers who had experienced extreme events were socially and geographically spatially close, and that psychological distance mediated emotional regulation among farmers—the mental processes through which individuals decide how emotions are expressed and experienced [3842]. Nevertheless, a recent meta-analysis performed by van Valkengoed and colleagues (2019) found that experience had small-to-moderate effects on climate change adaptation. Moreover, Larcom (2019) found that experience with a heatwave amongst the general population did not elicit adaptation behaviors in the United Kingdom, but did elevate concern [3943]. Similarly, Albright and Crow (2019) found that direct flooding experience was not a predictor of concern for climate change amongst Colorado, United States, residents [404]. These more recent studies suggest that there may be a threshold of individual exposure to natural hazards and their potential to catalyze

behavioral change; indeed, as the rate of extreme events increases, it could be probable that such events may saturate in people's experiences and become "more normal".

The aforementioned studies also highlight the importance of context, but also bring attention to how psychological distance of climate change research is limited in its generalizability [305]. Furthermore, most research on the matter hasve used data on reported experience, and not on direct experience (e.g. material impacts such as damage). Zanocco et al. (2018) performed case studies of residents whose communities were directly impacted by extreme events, such as tornados and wildfires, or were proximate to where the events occurred, in order to understand the relationship between experience of extreme event and views of climate change. Applying Construal Level Theory perspectives of psychological distance, they found that geographical spatial proximity to the event did not align with climate change views, but reported harm did [415]. This quote from Demski et al. (2017), as cited by Zanocco et al. (2018), underscores the aforementioned: "Self-reported measures of experience that include: direct physical experience and material impacts of an event, well-defined in terms of personal effects and damage, are less susceptible to biased reporting, providing the best proxy available for objective experience" (p. 152) [415] .

This paper attempts to fill several gaps in the research.. by assessing the psychological distance of climate change amongst Puerto Rican farmers after experiencing Hurricane Maria. First, we will assess Puerto Rican farmers' psychological distance of climate change after experiencing an extreme weather event. Hurricane Maria impactedaffected all of Puerto Rico in September 2017, causing bruntsignificant damages to infrastructure, agriculture, and livelihoods. The 2017 Atlantic Hurricane Season is noted for being a devastating one in the Caribbean region (42)[42]. To the best of our understanding, there is are no studiesy that looks atexploring the relationship

~~between actual experience with extreme events and its relationship with~~ the psychological distance of climate change amongst farmers in small island developing states and territories after an extreme weather event. Second, we will assess the extent to which ~~the~~ reported experience with Maria (both reported experience with other similar extreme events, and direct ~~damages caused~~damages reported by Hurricane Maria) relate to psychological distance of climate change. As stated above, ~~most research has looked on reported experience~~there are mixed results on how reported experience and actual experienced damages are linked to climate change perceptions. Third, in order to increase our understanding on the degree to which cognitive beliefs play a role in climate change adaptation and therefore relate to individual adaptive capacity, we examine farmers motivation to adopt adaptation practices, and its~~the~~ relationship to perceived capacity and vulnerability, as well to their ~~of~~ psychological distance of climate change. ~~to farmers' adaptation perceptions.~~

# The present study

This study focuses in Puerto Rico, an unincorporated territory of the United States that imports around 85% of the food it consumes, and that is going through a social-economic crisis because of its billion dollar debt and political situation [42~~6~~–45~~9~~]. In response to Puerto Rico's debt crisis in 2016 , the United States' government created a Fiscal Management and Oversight Board that imposed austerity measures, and made visible Puerto Rico's lack of political agency as a territory of the United States [19~~28~~,20~~9~~]. Simultaneous and previous to this change, the agricultural sector in Puerto Rico was experiencing positive changes, such as a rise in new farmers, production

increases, and increased awareness of food security [14~~23~~,45~~9–~~47~~51~~]. Such agricultural gains were important since Puerto Rico, like the broader Caribbean, was experiencing a steady decline in farms since the 1990's due to trade liberalization, globalization, and other external forces [~~5~~,15~~24~~,47~~51,~~ 48, 49~~52~~]. Nevertheless, such gains were erased when both Hurricane Irma and Hurricane Maria, two of the strongest in the Atlantic's history, hit Puerto Rico in September 2017 ~~(42,51–53)~~[42,51–53].

The local Department of Agriculture (2018) reported that both hurricanes caused $2 billion in total losses, with the majority of losses from Maria ($228 million in production losses, and $1.8 billion in agricultural infrastructure losses). Such damages significantly impacted Puerto Rican agriculture, which, according to the USDA, is comprised of many small-scale farmers with an average farm size of 59 acres (approximately 23 hectares) [50~~3~~], who focus mostly on domestic markets, and who have an average annual income of less than $20,000 [49~~52~~]. Focusing this study on Puerto Rican farmers after experiencing a major storm, not only allow us to advance our understanding of the psychological distance of climate change, but provides us the opportunity to produce important applied knowledge around strengthening adaptive capacity to safeguard local agriculture and food access [24~~17~~,51~~18~~].

## Objective, research questions, and hypotheses

Our study focuses on understanding how the psychological distance of climate change manifests in an island-archipelago setting, amongst a vulnerable population (farmers). Specifically, we intend to assess (1) the psychological distance of climate change amongst farmers in Puerto

Rico, an unincorporated territory of the United States and Caribbean non-UN member Small Island Developing State, after an extreme-weather event, (2) the extent to which psychological distance of climate change is related to reported experience, and reported damages caused by Maria,; and to (3) other cognitive beliefs important in adaptation research, such as motivation to adapt, perceived capacity (self-efficacy or perceived instrumentality), and perceived vulnerability. We ask: 1) How do farm and farmer characteristics, and direct experiences with extreme events (i.e. reported damages and reported experience with similar events) relate to the psychological distance of climate change? And, 2) in light of experiencing an extreme event, what is the role of psychological distance of climate change in motivating Puerto Rican farmers to adapt to climate change? Following research on the psychological distance of climate change, we hypothesize that farmers' psychological distance of climate change will be driven by their experience (e.g. reported damages) with Hurricane Maria (H1). Furthermore, we predict that psychological distance of climate change will be related to perceived capacity, perceived vulnerability, and motivation to adapt (H2). Fig 1 shows our hypothesized model.

**Fig 1. Hypothesized model.** Farm and farmer characteristics' variables included as controls.

# Methodology

## Data gathering

Previous studies of on-farm management and climate change perceptions, and on the psychological distance of climate change [13, 2417,22,3741,524] informed the development of a survey instrument, which was contextualized to Puerto Rico and the general objectives of the main study. This study was carried out in collaboration with the Extension Service of the University of Puerto Rico at Mayagüez. Thus, feedback from Extension partners were also included in the survey's development. The survey was translated into Spanish, and piloted in February 2018 with a pool of Puerto Rican farmers (n = 321), and was also shared amongst Extension agricultural agents for feedback—enumerators of the survey. Minimal language and structural changes were made to the instrument.

Surveys (n = 440) were randomly administered to farmers by Extension agents, according to the five regions the Extension Service divides Puerto Rico: Arecibo, Caguas, Mayagüez, Ponce, and San Juan. Each region has several agents that provide service to the municipalities that comprise each region. -Survey deployment was based on Extension Services' recommendations to access a wide range of farmers, and because agricultural agents had an established presence. Each regional office received a set of surveys; San Juan, Caguas, and Ponce received 100 surveys each, and Mayagüez and Arecibo received 75 surveys each. Participant Extension agents randomly surveyed farmers in the municipalities they worked in. Surveys were administered between May and July 2018 in person. Farmers from 65 of Puerto Rico's 78 municipalities answered the survey. A total of 405 surveys were completed, resulting in an 87% response rate based on the American Association for Public Opinion Response Rate Ccalculator (53)[53].

## Assessing the psychological distance of climate change.

Eight questions were used to assess the four dimensions of the psychological distance of climate change (temporal, social, geographicalspatial, and uncertainty) (Table 1). These questions were adapted from previous research on the topic, and contextualized to Puerto Rico (24,32,37,52)[24,32,37,52]. The temporal dimension was assessed with one item, while the social and geographical spatial dimensions were assessed with two each. Questions assessing the social and geographical spatial dimensions were intended to separately assess local and distant concerns. Uncertainty was assessed through three statements. Variables were reversed where needed, in order to analyze ascending levels of the psychological distance of climate change; "5" in the scale would represent "psychologically distant". We utilized a Cronbach's alpha for these items was assessedto measure internal validity of these constructs in the pilot analysis, demonstrating good internal reliability (alpha=0.86). in the pilot's results. Alpha was 0.86, which shows good internal validity. Furthermore, a factor analysis withusing principle components factor was carried out on these items to further assess consistency. S1 Table shows the pilot data's analysis results, which indicate all items aligned well under one factor (Factor loadings were >.40). This supported our decision to keep a variable scale in the study All items were included as a scale variable for the psychological distance of climate change—Cronbach's alpha for the main study's psychological distance of climate change scale was = 0.74, which suggests good internal validity (58)[58]. "Strongly agree" and "agree" answers for dimensions' statements were combined as "psychologically far" for ease of interpretation. The same was done for "psychologically close" with opposite answers. "Neutral" was kept separate.

## Assessing social and cognitive variables

Adaptation is a set of contextualized decisions that reflect individuals' adaptive capacity.~~ to a great extent~~. Given the role of social and cognitive variables ~~in~~in predicting adaptation behaviors amongst farmers ~~(10,22,24,26,52)~~[10,22,24,26,52], we included several variables that, to the best of our knowledge, have not been evaluated in Puerto Rico. Farmers were asked about their motivation to adapt to climate change through adopting new agricultural practices, their perceived capacity to do so, and their perceived vulnerability to extreme events (Table 1). ~~Table 1 shows the complete premises. All of them were 5-point Likert scales.~~

## Structural equation model

A structural equation model was built to explore the extent to which psychological distance of climate change relates to farmers' reported experience with similar events to Hurricane Maria and reported degree of damage due to Maria, and to their motivation to adapt to climate change. Structural equation models allow quantitative analysis of the direct, indirect, and mediated interactions between variables, and the incorporation of latent constructs [28, 55, 56~~6- 58~~]. Fig 1 shows the hypothesized model. Two variables assessed experience: "reported past experience" and "reported hurricane damages" (Table 1). Variables for perceived capacity and vulnerability, as well as for motivation to adapt, were included. Those variables, alongside psychological distance of climate change, were included to grasp an understanding of the role of cognitive beliefs in the context of adaptive capacity. The structural equation model was deployed in Stata 15.1, using Maximum Likelihood with bootstrapping (1000 iterations) to control for Type I

errors, for non-normal data, and to ~~and~~ increase statistical power ~~(28,57,58)~~[28,57,58]. ~~. (58)~~ The following Goodness-of-fit criteria were used: Chi-square (X2), Standardized Root Mean Square Residual (SRMR), Comparative fit index (CFI), Root mean square error of approximation (RMSEA) [56~~8~~].

**Table 1: Survey variables used in this study.**

| Category | Variable | Question/Statement | Measure |
|---|---|---|---|
| Climate change perceptions | Global climate | The global climate is changing. | 5-point Likert scale—from strongly disagree to strongly agree |
| | Global temperature | Average global temperatures are increasing. | 5-point Likert scale |
| | Anthropogenic causes | Human activities such as fossil fuel combustions are an important cause of climate change. | 5-point Likert scale |
| Psychological Distance of Climate Change | | | |
| Temporal Dimension | Today | The effects of climate change are already being felt today. | 5-point Likert scale |
| ~~Social dimension~~ | ~~Farmers~~ | ~~Farmers like me are not likely to be affected negatively by climate change.~~ | ~~5-point Likert scale~~ |

| Category | Variable | Question/Statement | Measure |
|---|---|---|---|
| | ~~General public~~ | ~~People who aren't farmers are likely to be affected negatively by climate change.~~ | ~~5-point Likert scale~~ |
| ~~Geographical~~ Spatial dimension | Local agriculture | Climate change does not presents more risk than benefits to agriculture in Puerto Rico. | 5-point Likert scale |
| | Global agriculture | Climate change presents more risks than benefits to agriculture globally. | 5-point Likert scale |
| Social dimension | Farmers | Farmers like me are not likely to be affected negatively by climate change. | 5-point Likert scale |
| | General public | People who are not farmers are likely to be affected negatively by climate change. | 5-point Likert scale |
| Hypothetical or u~~U~~ncertainty dimension | Impact uncertainty | There is scientific uncertainty about the potential impacts of climate change on agriculture. | 5-point Likert scale |
| | Cause uncertainty | There is scientific uncertainty about the causes of climate change. | 5-point Likert scale |

| Category | Variable | Question/Statement | Measure |
|---|---|---|---|
| | Hurricane uncertainty | I am uncertain that the occurrence of strong hurricanes in the Atlantic is related to climate change. | 5-point Likert scale |
| Cognitive beliefs | Motivation to adapt | I feel motivated to change my agricultural practices to prepare for future extreme weather events like Hurricane Maria. | 5-point Likert scale |
| | Perceived self-capacity | I feel that I have the capacity to change my agricultural practices to prepare for future potential extreme weather events like Hurricane Maria. | 5-point Likert scale |
| | Perceived vulnerability | I believe my farm is vulnerable to future extreme weather events like Hurricane Maria. | 5-point Likert scale |
| Experience with extreme weather events | Reported experience | I have faced similar extreme weather events like Hurricane Maria in the past ten years. | 5-point Likert scale |

| Category | Variable | Question/Statement | Measure |
| --- | --- | --- | --- |
| | Reported damages | How would you describe the damages, if any, caused by Hurricane Maria to your farm? | 5-point Likert—from no damages to total loss |
| Farmer and farm characteristics* | Age | In which year you were born? | Continuous |
| | Bonafide[a] | Do you participate in the bonafide program of the Puerto Rico Department of Agriculture? | Binary—yes or no |
| | Education level | What is the highest level of education you have completed? | Ordinal—from elementary school to PhD degree |
| | Farm size | How many *cuerdas*[1] of terrain do you manage in your farm? | Continuous |
| | Gender | What is your gender? | Binary—female or male |
| | Household income | What is your approximate household income, including all far and off-farm income? | Ordinal—from Less than $20,000 to more than $90,000 |

[a]The bonafide[a] program of the Puerto Rico Department of Agriculture provides farmers with agricultural incentives, and benefits then with various tax exemptions.

# Results

## Participants and the impact of Hurricane María

Farmers had an average age of 54, and farmed an average of 58 *cuerdas* (approximately 56 acres). The great majority of respondents had attended college (67%) (Table 2). Table 3 contrasts farmers' demographics and farm characteristics with those reported in Puerto Rico's 2017 Agricultural Census [503]. Respondents generally were similar to average census statistics, with the exception of having higher rates of formal higher education and somewhat higher percentage of income from farming. It is important to note that the recent census is not comparable to the previous one done in 2012 [503]. Moreover the 2017 census, performed in 2018, had a lower response rate, and USDA reported some difficulty in performing the census given post Hurricane Maria conditions [503,5960]. Farmer respondents were distributed throughout Puerto Rico with 15% in the Arecibo region, 22% in the Caguas region, 19% in the Mayagüez region, 25% in the Ponce region, and 22% in the San Juan region. (Percentages do not sum 100% because some farms extend beyond one municipality that border with another). The majority of farmers experienced damages from Hurricane María; 42.6% reported total loss of their farms, while 45.6% reported significant damages (Fig 2). The majority of farmers (55.1%) disagreed and strongly disagreed (hereafter disagreed) that they had faced similar events to Hurricane Maria.

**Table 2: Mean statistics of control variables.**

| Variable | Scale | Frequency (%) | Mean ± SD | n |
|---|---|---|---|---|
| Age | Continuous | - | 54.0 ± 13.5 | 391 |
| Bonafide | Yes | 210 (52.8) | - | 398 |
| | No | 188 (47.2) | | |
| Education level | Elementary School | 21 (5.2) | - | 401 |
| | Junior High School | 13 (3.2) | | |
| | Some High School | 15 (3.7) | | |
| | High School Diploma | 82 (20.5) | | |
| | Some College | 42 (10.5) | | |
| | Technical Degree | 25 (6.2) | | |
| | Associate Degree | 41 (10.2) | | |
| | Bachelor’s Degree | 109 (27.2) | | |
| | Master’s Degree | 46 (11.5) | | |
| | PhD | 7 (1.8) | | |
| Farm size | Continuous | - | 58.1 ± 98.5 | 383 |
| Gender | Female | 55 (14.0) | - | 395 |
| | Male | 340 (86.0) | | |
| Household income | Less than $20,000 | 138 (36.4) | - | 379 |
| | $20,000 - $40,999 | 125 (33.0) | | |
| | $41,000 - $60,999 | 52 (13.7) | | |
| | $61,000 - $80,999 | 36 (9.5) | | |

| Variable | Scale | Frequency (%) | Mean ± SD | n |
|---|---|---|---|---|
| | More than $90,000 | 28 (7.4) | | |

**Table 3: Demographic variables of our study's surveyed farmers in comparison to USDA 2017 Agricultural Census data for Puerto Rico.**

| Category | Present study | USDA 2017 Census data |
|---|---|---|
| Average farm size | 58.1 *cuerdas* (majority farmed > 20 *cuerdas*) | 59.3 *cuerdas* (majority farmed > 20 *cuerdas*) |
| Years farming | Majority > 10 years | Majority > 10 years |
| Average age | 54 | 61 |
| Gender | Great majority is male | Great majority is male |
| Education | Majority reported High School Diploma or more | Majority reported High School Diploma or less |
| Income from farming | > 50% | < 50% |
| Household income | Majority reported < $20,000 | Majority reported < $20,000 |

Note: Data from the census is for farms' principal operators. In Puerto Rico, this does not necessarily ~~means~~mean that they are the sole owner of the farm. Neither the census or the local Department of Agriculture detail the number of Puerto Rican farmers who participate in the bonafide program; however, in a media report in 2019 (64), according to the Secretary of Agriculture, Carlos Flores, among Puerto Rico's 17,000 farmers, 4,000 are part of the bonafide program; 24% in comparison to the 53% in our study. It is also important to note that the Secretary's comment contrast with the number of farmers provided by the USDA census, which was conducted in 2018, and had a low response rate (54).

**Fig 2: Farmer~~'~~s' past experience with events similar to Hurricane Maria (top) and reported hurricane damages (bottom).**

Fig 3 shows results for farmers' perceptions around climate change and adaptation. An overwhelming majority of Puerto Rican farmers believe that the global climate is changing (86% strongly agree and agree~~; hereafter agreed~~), and that it has anthropogenic causes (91.4%). As well, 84.4% of Puerto Rican farmers agreed and strongly agreed that they feel motivated to change their agricultural practices to better prepare for future extreme events. Though farmers perceive their farms to be vulnerable to future extreme weather events (93.5%), they understand themselves capable to adapt to climate change (79.0%).

**Fig 3: Climate change perceptions and behavioral motivations among Puerto Rican farmers.**

## Puerto Rican farmers' psychological distance of climate change

We found that Puerto Rican farmers understand climate change as both a local and global phenomenon, being both psychologically close and psychologically distant across various measures. While we find evidence across all four dimensions of psychological distance for perceived "closeness", we also find that farmers demonstrate awareness for climate change effects beyond Puerto Rico in all dimensions (psychologically far) (Fig 4).

**Fig 4: Results for variables of the psychological distance of climate change.** ~~To ease understanding, answers were collapsed: “agree” and “strongly agreed” are shown as “psychologically far”, and “disagree” and “strongly disagree” are shown as “psychologically close”.~~

Respondents demonstrated a temporally close relationship to climate change with the majority disagreeing and strongly disagreeing (95.1%) that the effects of climate change are not being felt today. Regarding the ~~geographical~~ spatial dimension, Puerto Rican farmers perceive that climate change will both negatively impact agriculture at both local and global scales (Fig 4). The majority disagreed and strongly disagreed (89.4%) with the statement, ~~that~~ “climate change does not present~~s~~ more risks than benefits to agriculture in Puerto Rico” (e.g. Puerto Rico’s agriculture is at risk). Farmers also agreed and strongly agreed (90.0%) with the statement focused on the global scale, “Climate change presents more risks than benefits to agriculture globally”. At the social dimension, farmers expressed that climate change will both affect farmers (people like them), and the general public (people not like them). The majority (92%) disagreed and strongly disagreed that farmers like them will likely be affected negatively by climate change (psychologically close); 89% agreed and strongly agreed with the ~~that~~ ~~people~~statement, “people who aren’t farmers are~~will also~~ likely to be ~~affected~~ negatively affected by climate change” (psychologically far).

We found the greatest variability in the uncertainty or hypothetical dimension~~s~~ of psychological distance of climate change. Thirty nine percent (39%) of respondents agreed and strongly agreed

that there is scientific uncertainty about the potential impacts of climate change on ~~agriculture~~Puerto Rico, 14% were neutral, while 47% disagreed and strongly disagreed with the statement. The majority (60%) disagreed and strongly disagreed that there is scientific uncertainty about the causes of climate change; 16% were neutral, and 24% agreed and strongly agreed with the statement. Regarding the statement related to feeling uncertain about the relationship between climate change and occurrence of strong hurricanes in the Atlantic, 25% agreed and strongly agreed, 13% were neutral, and 63% disagreed and strongly disagreed. Furthermore, respondents overwhelmingly agreed that human activities are an important cause of climate change (3.2% disagreed and strongly disagreed, 5.4% were neutral, and 91.4% agreed and strongly agreed).

## Structural equation model

The structural equation model (S2~~1~~ table, S3~~2~~ table, S4~~3~~ table, and fig 5) demonstrates that four of the seven hypothesized pathways were statistically significant ($p < 0.05$); S1 table~~-~~ shows results for all variables, including control variables. Goodness-of-fit criteria suggests the model is acceptable: RMSEA = 0.059, CFI = 0.861, SRMR = 0.058. Furthermore, the model showed that for motivation to adapt, our main dependent variable, $R^2 = 0.410$.

**Figure 5: Structural equation model results for the hypothesized model.** Thick arrows show significance ($p < 0.05$). Standardize coefficients are also shown (*b*). The model controls for farmer and farm characteristics, including age, gender, household income, farm size, education, and bonafide. Goodness-of-fit criteria suggest an overall good fit: RMSEA = 0.059, CFI = 0.861,

SRMR = 0.058. $R^2$ for motivation to adapt was 0.410. Results of indirect effects and total effects are in S2 table and S3 table, respectively.

The model shows that ~~direct experience (~~reported hurricane damages~~)~~ and reported past experience with similar extreme events to Maria are not significantly predictive of the psychological distance of climate change amongst Puerto Rican farmers, a rejection of H1. Moreover, none of the control variables (age, gender, farm size, bonafide, education, and income) were linked to the psychological distance of climate change (S2~~1~~ table). However, both perceived capacity and vulnerability are significantly negatively related to the psychological distance of climate change ($p < 0.05$) (H2). In other words, farmers that perceived climate change as more distant were more likely to report lower rates of perceived capacity ($b$ = -0.140, p = 0.017), and were less likely to report higher rates of perceiving their farms as vulnerable to future extreme weather events ($b$ = -0.164, p = 0.011). We find no significant direct relationship between psychological distance of climate change and motivation to adapt; however, both perceived capacity ($b$ = 0.578, p = 0.000) and perceived vulnerability ($b$ = 0.256, p = 0.000) were positively linked to motivation to adapt.

We find no significant relationships between control variables and reported past experience with similar extreme events (S2~~1~~ Table). Regarding reported hurricane damages, farmers who are participants of the bonafide program of the Puerto Rico Department of Agriculture were more likely ($b$ =0.134, p = 0.034) to report higher rates of damages to their farms due to Hurricane Maria. Furthermore, education level was negatively linked to reported damages by Hurricane Maria ($b$ = -0.177, p = 0.002), meaning that~~,~~ increasing levels of formal education were linked to reporting lower rates of damages.

# Discussion

This study examined Puerto Rican farmer~~’~~s’ psychological distance of climate change, and its relationship to reported damages following Hurricane Maria and reported experience with past extreme weather events, in order to better understand potential drivers and barriers to strengthen adaptive capacity. We expand the current literature to utilize psychological distance of climate change beyond continental and high-income countries, and to understand how farmers’ psychological distance of climate change relates to other cognitive beliefs in a vulnerable island-archipelago setting. We found that Puerto Rican farmers recognize climate change as a local and global issue (fig 4). Hence, they are neither psychologically close nor far, but psychologically aware of climate change’s impacts across multiple time frames, geographies, and social constructs. We also found that neither reported experience with past extreme weather events, nor the ~~direct~~ reported damages effected by Maria were linked to farmers’ psychological distance of climate change. Furthermore, farmers overwhelmingly perceived themselves self-capable and motivated to adapt to climate change. Nevertheless, motivation was not directly linked to their psychological distance of climate change.

Studies have used psychological distance to climate change to better understand whether this framing can prompt individual mitigation or adaptation actions. Moreover, the trend in the literature is that reduced psychological distance—mainly through experience with extreme weather events—will correlate with action [24, 30–32, 35, 37, 51~~17–19,35,36,39,41~~]. Our results provide a different picture—we found that Puerto Rican farmers cannot be characterized as

psychologically close or psychologically distant to climate change, but are psychologically aware of the impacts related to climate change at different scales. Furthermore, our model we showed that variables assessing hurricane damage and reported experience with similar extreme weather events direct experience with extreme weather events may not be the principal link to reduceexplain farmers' psychological distance to climate change (Fig 5). As well, psychological distance did not have a direct effect on motivation to adapt, but it did have an indirect effect on it through perceived self-capacity and vulnerability (S32 table and S43 table).

These results show that Puerto Rican farmers recognize climate change as being both near and far, which indicates that in the Caribbean context, where climate change is recognized as happening (61,62)[61,62], a psychological distance framing may not be relevant.- Our results on overall climate change perceptions, and farmers' psychological distance of climate change, align with a study by the Puerto Rican Department of Natural Resources that state that Puerto Ricans believe in, are aware of, and are concerned about climate change [63]. Puerto Rican farmers, overwhelmingly, understand that the climate is changing. This awareness of climate change as a threat is strong in Latin America and the Caribbean (61,62)[61,62], where farmers and non-farmers are aware of the impacts related to climate change [62, 64, 6564–66]. Surveyed farmers understand that climate change is happening now (temporal), that it will affect farmers and non-farmers (social), and that it will affect local and global agriculture (geographicalspatial). These results are in contrast to some existing research from the mainland of the U.S., where climate change belief among farmers is not nearly as high, especially in the perception of its anthropogenic causes [(e.g.(66,67)]). -Conversely, these results are more aligned with farmer perceptions of climate change from low-income countries, where climate change belief is overwhelmingly high (e.g.(68,69)[e.g. 68,69]. ) Theise results could suggest that when a

threshold for climate change belief is passed (potentially achieved by continued exposure to extreme events or a collective social understanding), individuals recognize it as a problem that goes beyond their own self-interest. Furthermore, it could be that there is an understanding of the interactions (e.g. feedback loops) and interconnectedness amongst scales in and beyond a place, and indicates that there may be a saturation point for the psychological distance of climate change framing. In other words, when enough people recognize ~~the~~ climate change as a problem, both locally and globally, it may no longer catalyze action for climate mitigation or adaptation~~—~~ ~~–~~other barriers may exist. This should be further studied.

Our respondents were heavily affected by Hurricane María; 42.6% reported total loss, while 45.6% reported significant losses. Given the absolute prevalence of damage across our population, these outcomes may relate to the fact that we find no relationship of reported damage to psychological distance. It is important to underscore that the last hurricane to directly impact Puerto Rico before Maria was 1998's Hurricane Georges. Moreover, Puerto Rico, as well as the Caribbean, have experienced concurrent hazards and been subject to non-landing hurricanes ~~(4,70,71)~~[4,70,71]. And given the age span of our participants, assuming they have lived in Puerto Rico, we can say that they have experienced over 50 tropical storms and hurricanes ~~(70,71)~~[70,71]. ~~-~~As such, these results ~~might~~ further lend evidence to the idea of a "extreme events" ~~suggest that there may also~~~~could~~ ~~be a~~ threshold ~~for level of damage and frequency of extreme events~~ ~~.~~, whereby a certain frequency and intensity of extreme events no longer impacts climate change belief or ~~that may then fail to impact~~ psychological distance. ~~-When a group of people consistently face extreme events, especially ones that are wholly devastating, they could no longer think about such events as being close and driving climate change perceptions~~. Nevertheless, ~~we saw~~ "experience" ~~here~~in this study reported ~~as reporting~~ actual damage by the

hurricane, as well as reporting experiencing similar events to Maria in the past 10 years. Given that experience has been measured in different ways, resulting in varied results, a future study should inquire deeply about what it means to "experience" an extreme event in Puerto Rico (72,73)[72,73]. Furthermore, looking at experience and climate change perceptions from a qualitative perspective could also elicit a broader understanding about their linkage to experience and adaptation (74)[74].

Our structural equation model showed that farmers who are bonafide participants in the Puerto Rico Department of Agriculture (52.8%) were more likely to report higher rates of damages ($R^2$ = .050). To participate in that program, 50% or more of farmers' income must come from agriculture. Farmers who are bonafide are exempted from some state and municipal taxes, and have access to other agricultural incentives. In our study, those that were bonafide had an average farm size of 85 *cuerdas*, and 76% of their income came from agriculture. This could point to the relationship of structural determinants' (e.g. policy structures) to climate change's effects on farmers. We did not have variables looking at type of agricultural incentives, or access to governmental, community-based resources or assets; future studies could look at how policy structures, coupled with federal laws and Puerto Rico's context as a US territory, impact farmers' adaptive capacity. Furthermore, another line of research could focus on the extent to whichh dependence on Puerto Rico Department of Agriculture incentives relate to on-farm management and farm systems. This is timely given that Puerto Rico's recent incentives code, Law 60 of 2019, changed various regulations that have been in place for over 30 years [7567]. Furthermore, given that farmers overwhelmingly reported feeling self-capable and motivated to adapt—variables that have been shown positively linked to decision-making around climate change adaptation [2533]—future research and practitioners could focus on actual adaptation of

agricultural practices (beyond intentions and cognitive factors), and those drivers and barriers at broader levels (e.g. community, institutional, regional, national) to strengthen adaptive capacity. The structural equation model (Fig 5) shows that perceiving climate change to be psychologically distant is linked to reporting lower rates of perceived self-capacity. The same was shown for ~~and~~ perceived vulnerability to extreme weather events; the more distant climate change is perceived, the less vulnerable farmers perceived their farms to be to the impacts of future extreme weather events. ~~This aligns with the psychological distance of climate change assumptions on how one's awareness relates to our promptness to act to mitigate or adapt to climate change~~. Nevertheless, farmers' psychological distance of climate change (and other variables in the model) does not explain much of the variability in both perceived self-capacity ($R^2 = 0.019$) and vulnerability ($R^2 = 0.026$). This further suggests that in areas constantly affected by extreme weather events, other variables beyond the ones explored here may be more relevant in explaining and fostering adaptation. Farmers in Latin America, the Caribbean, and Africa have expressed concern for climate change at different scales, but such perceptions are poorly linked to actual climate change adaptation [38, 64, 65, 76~~42,65,66,68~~]. Puerto Rican farmers overwhelmingly feel motivated to adapt to climate change, and that is positively linked to perceived self-capacity and perceived vulnerability. Nonetheless, taking into account the damage caused by Hurricane María and its aftermath, as well as the poor subsequent recovery efforts, our findings suggest that to strengthen farmers' adaptive capacity we must look at other determinants beyond the individual level.

In small island developing states and territories, where climate change impacts are already being felt to a greater extent than in high-income continental countries, cognitive beliefs nor psychological distance to climate change may be strong components of adaptive capacity.

Farmers in Latin America [64~~5~~,65~~6~~], and the Caribbean [76~~68~~,77~~69~~] have been noted to lack institutional structures of support. In Puerto Rico, Perfecto and colleagues (2019) found that social capital and support networks were pivotal for Puerto Rican coffee farmers' recovery after Hurricane María, and that their agroecological practices, such as agroforestry, and other management styles were not sufficient for farms to be resilient and resistant within the catastrophic context of Hurricane Maria [78~~0~~]. Hurricane María affected all of Puerto Rico, causing 2,975 deaths [79~~1~~], and decimated the archipelago's agriculture. As such, we argue that reasons and circumstances beyond the self (cognitive beliefs) deserve more attention to better understand Puerto Rican farmers' decision-making, and increase their adaptive capacity. This conclusion aligns with a recent review by Wilson and colleagues (2020), which showed that studies on adaptation have taken two roads:~~,~~ one that seeks to understand the role of social and cognitive variables in adaptation behaviors, and another on how structural factors enact adaptation ~~(80)~~[80]. It is important to fill in the research gap on how societal and governance structures interact with social and cognitive beliefs in eliciting adaptation behaviors [80].

Taken results together, our study~~these results~~ shows that the psychological distance of climate change may not be ~~-~~an appropriate framework to understand cognitive beliefs' role in adaptive capacity, especially in island settings, where ~~in an island setting, but potentially increasingly as~~ climate change beliefs are overwhelmingly recognized.~~might be becominge saturated, and thus, not being important drivers in adaptation towards acknowledgement of the issue~~.

Outside of island contexts, growing public sentiment ~~is growing~~ that climate change is widely accepted as an important phenomenon is growing. ~~We understand we are reaching a point where climate change-related conversations and perceptions are widely accepted. Future research could~~

~~further generate understanding on that.~~ While f~~F~~armers in island and disadvantaged areas, who are at the forefront of constant climate change-related impacts, ~~might~~ may have already reached a climate change belief saturation, it could be forthcoming in other regions and types of people. These results suggest that, if such things occur, climate change belief will likely not ~~gotten to a point where experiencing climate change is not the~~be the most important driver of ~~concern nor motivation to adapt to climate change~~climate change adaptation. Instead, structural, political, and other non-individual barriers to adaptation may be increasingly important. In the case of ~~.~~ Caribbean farmers, they face institutional barriers to better adapt to climate change [14, 15, 45, 47, 81~~6,23,24,49,51~~], and are localized in a region that still today is subject to colonial relationships and power dynamics that increase their vulnerability to natural hazards [~~28~~19–21~~30~~]. It is important to understand the degree to which psychological awareness (not distance) of climate change relates to other cognitive beliefs and decision-making, but this should co-exist with further efforts to more clearly examine non-individual barriers to adaptation.~~cannot be done through ignoring the circumstances in which farmers are embedded~~. Climate change adaptation cannot be reduced to something that is dependent on the individual because then we could be contributing on the assertion that the genesis of vulnerability lies on the individual [8~~7~~2]. Vulnerability to natural hazards is a systemic issue, and farmer~~'~~s' adaptation to climate change, as a decision-making process, is subject to structural interactions that cannot be overlooked.

# Limitations

We note some limitations in this study. First, to the best of our knowledge, there is no previous study looking at Puerto Rican or Caribbean farmers' psychological distance of climate change

before and after an extreme weather event. We lack information on the issue before Hurricane Maria or other events. This limits our ability to understand if their psychological awareness is driven by experience. Nonetheless, Extension Service's internal data before Hurricane Maria showed that farmers were aware of climate change's impacts at different scales. Second, farmers surveyed showed higher percentage of income from farming than that reported in census data [53], and we had an overrepresentation of bonafide farmers. Nevertheless, most of the demographic factors of surveyed farmers here align with those of the latest agricultural census for Puerto Rico (table 3). Third, the variables we used to measure experience might not have capture a nuanced understanding of experience with extreme weather events ~~(75)~~[75]. Nonetheless, we use variables that have been used in past research regarding the psychological distance of climate change. Furthermore, given that most farmers surveyed for this study receive information from, or are linked to the Extension Service, this might suggest that they have higher access to information and resources, which may affect their understanding of climate change and other related issues. As research in island systems on this topic continues to grow, there is opportunity for broader engagement with farmers, including those not directly integrated with ~~Cooperative~~ the Extension Service.

# Conclusion

Farmers' adaptive capacity must be strengthened to decrease their vulnerability to natural hazards. In order to do so, cognitive beliefs ~~must~~ should be considered given that climate change adaptation—a set of decisions at the individual level—is one key action to increase adaptive capacity. Here we assessed Puerto Rican farmers' climate change adaptation perceptions after

Hurricane Maria to understand the role of experience with extreme events on the psychological distance of climate change, and its relation to motivation to adapt. We found that Puerto Rican farmers are psychologically aware of climate change impacts at different levels—local to global~~—and that such awareness is not driven by their experience with extreme events~~. These findings contrast with research in continental and high-income countries that seeks to reduce psychological distance to climate change amongst individuals to prompt mitigation and adaptation actions. Furthermore, we expand literature cemented on the assumption that being psychologically close enacts change, and that such psychological distance is driven by experience. We showed that neither reported experience with extreme events, nor farmers’ ~~actual experienced~~ reported damages by Hurricane Maria were determinants of farmer~~’~~s’ psychological distance of climate change. Furthermore, here we demonstrate that context matters, and that the adaptive capacity of individuals embedded in vulnerable contexts—farmers in an island disproportionately exposed and sensitive to climate change’s impacts—is not highly dependable on individual actions. We suggest that variables, such as institutional frameworks of support, must be better understood. Furthermore, research on the psychological distance of climate change should consider that we ~~might~~ may have reached a ~~be arriving to a~~ threshold where climate change perceptions are not significant drivers of change. Hence, we suggest that understanding peoples’ “psychological awareness of climate change” and its relation to context could provide a more nuance comprehension of climate change adaptation than focusing on how being “close” or “far” enacts change.

# Acknowledgements

We acknowledge all the Puerto Rican farmers that participated in this project, and the Extension Service's agricultural agents who enumerated the surveys. We are grateful to Dr. Aníbal Ruiz-Lugo, assistant ~~interim~~ dean of the Extension Service, for leading the logistics and efforts on-site to carry out this project. Much thanks to Maritzabel Morales and the administrative team of the Extension Service for scanning all the surveys and assisting with administrative logistics, and to Olivia Peña for assisting in data entry. We are also thankful to Drs. María del Carmen Rodríguez-Rodríguez, Robinson Rodríguez-Pérez, and Fernando Pérez-Muñoz for assisting in the completion of the project. We are grateful to Dr. Jim Vigoreaux, Agro. Kiria Hurtado, and Carmen González-Toro for proofreading the Spanish version of the survey. We also acknowledge the colleagues who read the first draft of this paper, and provide us with meaningful review comments. Specially, we thank Amy Trubek, Teresa Mares, Maya Moore, Carolyn Hricko, and Caitlin B. Morgan. Much thanks as well to the University of Vermont's Food Systems Graduate Program and College of Agriculture and Life Sciences for providing funding in a timely manner to complete this research.

# References

1. Adger WN. Vulnerability. Global Environmental Change. 2006 Aug;16(3):268–81.

2. Wisner B, Gaillard J, Kelman I. Framing disaster: Theories and stories seeking to understand hazards, vulnerability and risk. In: The Routledge Handbook of Hazards and Disaster Risk Reduction. 1st ed. London; New York: Routledge; 2012. p. 18–33.
3. López-Marrero T. An integrative approach to study and promote natural hazards adaptive capacity: a case study of two flood-prone communities in Puerto Rico: An integrative approach to study and promote natural hazards adaptive capacity. Geographical Journal. 2010 Feb 25;176(2):150–63.
4. López-Marrero T, Wisner B. Not in the Same Boat: Disasters and Differential Vulnerability in the Insular Caribbean. Caribbean Studies. 2012;40(2):129–68.
5. Adger WN, Vincent K. Uncertainty in adaptive capacity. Comptes Rendus Geoscience. 2005 Mar;337(4):399–410.
6. Smit B, Wandel J. Adaptation, adaptive capacity and vulnerability. Global Environmental Change. 2006 Aug;16(3):282–92.
7. Brooks N, Adger WN. Assessing and Enhancing Adaptive Capacity. In: Adaptation Policy Frameworks for Climate Change: Developing Strategies, Policies and Measures. Cambridge, UK: Cambridge University Press; 2005. p. 165–82.
8. Wisner B, Blaikie P, Cannon T, Davis I. At Risk: Natural hazards, people's vulnerability and disasters. 2nd ed. New York and London: Routledge; 2004.
9. Holt-Giménez E. Measuring farmers' agroecological resistance after Hurricane Mitch in Nicaragua: a case study in participatory, sustainable land management impact monitoring. Agriculture, Ecosystems & Environment. 2002 Dec;93(1–3):87–105.
10. Shinbrot XA, Jones KW, Rivera-Castañeda A, López-Báez W, Ojima DS. Smallholder Farmer Adoption of Climate-Related Adaptation Strategies: The Importance of

Vulnerability Context, Livelihood Assets, and Climate Perceptions. Environmental Management. 2019 May;63(5):583–95.

11. Adger WN. Social Capital, Collective Action, and Adaptation to Climate Change. Economic Geography. 2003;79(4):387–404.

12. Fernandez M, Méndez VE. Subsistence under the canopy: Agrobiodiversity's contributions to food and nutrition security amongst coffee communities in Chiapas, Mexico. Agroecology and Sustainable Food Systems. 2019 May 28;43(5):579–601.

13. Niles MT, Mueller ND. Farmer perceptions of climate change: Associations with observed temperature and precipitation trends, irrigation, and climate beliefs. Global Environmental Change. 2016;39:133–42.

14. Álvarez-Berríos NL, Soto-Bayó S, Holupchinski E, Fain SJ, Gould WA. Correlating drought conservation practices and drought vulnerability in a tropical agricultural system. Renewable Agriculture and Food Systems. 2018 Jun;33(3):279–91.

15. Lowitt K, Hickey GM, Saint Ville A, Raeburn K, Thompson-Colón T, Laszlo S, et al. Factors affecting the innovation potential of smallholder farmers in the Caribbean Community. Regional Environmental Change. 2015 Oct;15(7):1367–77.

16. Rosset PM, Sosa BM, Jaime AMR, Lozano DRÁ. The Campesino-to-Campesino agroecology movement of ANAP in Cuba: Social process methodology in the construction of sustainable peasant agriculture and food sovereignty. Journal of Peasant Studies. 2011;38(1):161–91.

17. Saint Ville AS, Hickey GM, Phillip LE. Institutional analysis of food and agriculture policy in the Caribbean: The case of Saint Lucia. 2017 [cited 2018 Aug 15]; Available from: http://dx.doi.org/10.1016/j.jrurstud.2017.03.004

18. Scandurra G, Romano AA, Ronghi M, Carfora A. On the vulnerability of Small Island Developing States: A dynamic analysis. Ecological Indicators. 2017;84:382–92.

19. Borges-Méndez R, Caron C. Decolonizing Resilience: The Case of Reconstructing the Coffee Region of Puerto Rico After Hurricanes Irma and Maria. Journal of Extreme Events. 2019 Mar;06(01):1940001.

20. Félix G, Holt-Giménez E. Hurricane María: An Agroecological Turning Point for Puerto Rico? Food First Backgrounder. 2017;23(4).

21. Moulton AA, Machado MR. Bouncing Forward After Irma and Maria: Acknowledging Colonialism, Problematizing Resilience and Thinking Climate Justice. Journal of Extreme Events. 2019 Mar;06(01):1940003.

22. Grothmann T, Patt A. Adaptive capacity and human cognition: The process of individual adaptation to climate change. Global Environmental Change. 2005 Oct;15(3):199–213.

23. Ostrom E. Polycentric systems for coping with collective action and global environmental change. Global Environmental Change. 2010 Oct;20(4):550–7.

24. Niles MT, Lubell M, Brown M. How limiting factors drive agricultural adaptation to climate change. Agriculture, Ecosystems and Environment. 2015;200:178–85.

25. van Valkengoed AM, Steg L. Meta-analyses of factors motivating climate change adaptation behaviour. Nature Climate Change. 2019 Feb;9(2):158–63.

26. Gebrehiwot T, van der Veen A. Farmers Prone to Drought Risk: Why Some Farmers Undertake Farm-Level Risk-Reduction Measures While Others Not? Environmental Management. 2015 Mar;55(3):588–602.

27. Bagagnan A, Ouedraogo I, M. Fonta W, Sowe M, Wallis A. Can Protection Motivation Theory Explain Farmers’ Adaptation to Climate Change Decision Making in The Gambia? Climate. 2019 Jan 14;7(1):13.

28. Luu TA, Nguyen AT, Trinh QA, Pham VT, Le BB, Nguyen DT, et al. Farmers’ Intention to Climate Change Adaptation in Agriculture in the Red River Delta Biosphere Reserve (Vietnam): A Combination of Structural Equation Modeling (SEM) and Protection Motivation Theory (PMT). Sustainability. 2019 May 27;11(10):2993.

29. Spence A, Poortinga W, Butler C, Pidgeon NF. Perceptions of climate change and willingness to save energy related to flood experience. Nature Climate Change. 2011;1(1):46–9.

30. Brügger A. Understanding the psychological distance of climate change: The limitations of construal level theory and suggestions for alternative theoretical perspectives. Global Environmental Change. 2020 Jan;60:102023.

31. McDonald RI, Chai HY, Newell BR. Personal experience and the “psychological distance” of climate change: An integrative review. Journal of Environmental Psychology. 2015;44:109–18.

32. Spence A, Poortinga W, Pidgeon N. The Psychological Distance of Climate Change. Risk Analysis. 2012;32(6):957–72.

33. Trope Y, Liberman N. Construal-Level Theory of Psychological Distance. Psychological Review. 2010;117(2):440–63.

34. Liberman N, Trope Y. The Psychology of Transcending the Here and Now. Vol. 322, Source: Science, New Series. 2008.

35. Brügger A, Morton TA, Dessai S. "Proximising" climate change reconsidered: A construal level theory perspective. Journal of Environmental Psychology. 2016;46:125–42.
36. Jones C, Hine DW, Marks ADG. The Future is Now: Reducing Psychological Distance to Increase Public Engagement with Climate Change. Risk Analysis. 2017;37(2):331–41.
37. Singh AS, Zwickle A, Bruskotter JT, Wilson R. The perceived psychological distance of climate change impacts and its influence on support for adaptation policy. Environmental Science & Policy. 2017;73:93–9.
38. Acharibasam JW, Anuga SW. Psychological distance of climate change and mental health risks assessment of smallholder farmers in Northern Ghana: Is habituation a threat to climate change? Climate Risk Management. 2018;21:16–25.
39. Larcom S, She P-W, van Gevelt T. The UK summer heatwave of 2018 and public concern over energy security. Nature Climate Change. 2019 May;9(5):370–3.
40. Albright EA, Crow D. Beliefs about climate change in the aftermath of extreme flooding. Climatic Change. 2019 Jul;155(1):1–17.
41. Zanocco C, Boudet H, Nilson R, Satein H, Whitley H, Flora J. Place, proximity, and perceived harm: extreme weather events and views about climate change. Climatic Change. 2018;149(3–4):349–65.
42. Bang HN, Miles LS, Gordon RD. Hurricane Occurrence and Seasonal Activity: An Analysis of the 2017 Atlantic Hurricane Season. American Journal of Climate Change. 2019;08(04):454–81.
43. Bueno R. Puerto Rico, Climatic Extremes, and the Economics of Resilience. Climate Policy Brief. 2017;(6).

44. Bueno R, Herzfeld C, Stanton E a., Ackerman F. The caribbean and climate change: The costs of inaction [Internet]. Stockholm Environment Institute and Global Development and Environment Institute, Tufts University; 2008 [cited 2018 Apr 12] p. 35. Available from: http://www.sei-us.org/climate-and-energy/Caribbean_Inaction_Cost.htm%5Cnhttp://www.gdae.org/CaribbeanClimate.html

45. Carro-Figueroa V. Agricultural Decline and Food Import Dependency in Puerto Rico: A Historical Perspective on the Outcomes of Postwar Farm and Food Policies. Caribbean Studies. 2002;30(2):77–107.

46. Gould WA. Caribbean Regional Climate Sub Hub Assessment of Climate Change Vulnerability and Adaptation and Mitigation Strategies. 2015;67.

47. Myrna Comas-Pagán. Plan de sguridad alimentaria. San Juan, Puerto Rico: Puerto Rico Department of Agriculture; 2014 p. 40.

48. Irizarry-Ruiz C. Desafíos de la agricultura en Puerto Rico para garantizar la seguridad alimentaria. In: Retos que conlleva la reutilización de aguas residuales en la agricultura. San Juan, Puerto Rico; 2016.

49. FAO. Food security and nutrition in small island developing states (SIDS). Policy Paper. 2014;16.

50. Weis T. Small farming and radical imaginations in the Caribbean today. Race & Class. 2007 Oct;49(2):112–7.

51. López-Marrero T, Castro-Rivera A. Actividad ciclónica en Puerto Rico y sus alrededores: 1867 al 2017. Mayagüez, Puerto Rico: Centro Interdisciplinario de Estudios del Litoral; 2018. 12 p.

**Formatted:** Font: (Default) Times New Roman, 12 pt, Spanish (Puerto Rico)

**Formatted:** Font: (Default) Times New Roman, 12 pt

**Formatted:** Font: (Default) Times New Roman, 12 pt, Spanish (Puerto Rico)

52. Perfecto I, Hajian-Forooshani Z, Iverson A, Irizarry AD, Lugo-Perez J, Medina N, et al. Response of Coffee Farms to Hurricane Maria: Resistance and Resilience from an Extreme Climatic Event. Scientific Reports [Internet]. 2019 Dec [cited 2019 Nov 5];9(1). Available from: http://www.nature.com/articles/s41598-019-51416-1
53. Puerto Rico's Agriculture and Farmers Decimated by Maria - The New York Times [Internet]. [cited 2020 Sep 25]. Available from: https://www.nytimes.com/2017/09/24/us/puerto-rico-hurricane-maria-agriculture-.html
54. USDA NASS. 2017 Census of Agriculture - Puerto Rico Data [Internet]. 2020 [cited 2020 Jun 12] p. 117. Available from: https://www.nass.usda.gov/Publications/AgCensus/2017/index.php
55. Ray A, Hughes L, Konisky DM, Kaylor C. Extreme weather exposure and support for climate change adaptation. Global Environmental Change. 2017;46:104–13.
56. Haden VR, Niles MT, Lubell M, Perlman J, Jackson LE. Global and Local Concerns: What Attitudes and Beliefs Motivate Farmers to Mitigate and Adapt to Climate Change? PLoS ONE [Internet]. 2012 [cited 2018 Sep 3];7(12). Available from: www.plosone.org
57. Response Rates - An Overview - AAPOR [Internet]. [cited 2020 Sep 23]. Available from: https://www.aapor.org/Education-Resources/For-Researchers/Poll-Survey-FAQ/Response-Rates-An-Overview.aspx
58. Nunnally JC. Psychometric Theory. McGraw-Hill; 1978.
59. Iacobucci D. Everything you always wanted to know about SEM (structural equations modeling) but were afraid to ask. Journal of Consumer Psychology. 2009 Oct;19(4):673–80.

Formatted: Font: (Default) Times New Roman, 12 pt

60. Schreiber JB, Nora A, Stage FK, Barlow EA, King J. Reporting Structural Equation Modeling and Confirmatory Factor Analysis Results: A Review. The Journal of Educational Research. 2006 Jul;99(6):323–38.

61. Tang J-W, Chen M-L, Chiu T-H. An Exploratory Study on Local Brand Value Development for Outlying Island Agriculture: Local Food System and Actor-Network Theory Perspectives. Sustainability. 2018 Nov;10(11):4186.

62. Lai K. Estimating Standardized SEM Parameters Given Nonnormal Data and Incorrect Model: Methods and Comparison. Structural Equation Modeling: A Multidisciplinary Journal. 2018 Jul 4;25(4):600–20.

63. USDA NASS. USDA Releases 2018 Puerto Rico Census of Agriculture [Internet]. 2020 [cited 2020 Jun 12]. Available from: https://www.nass.usda.gov/Newsroom/2020/06-09-2020.php

64. Díaz Rolón A. Aspira a que haya más agricultores bonafide [Internet]. El Vocero de Puerto Rico. 2019 [cited 2020 Jun 12]. Available from: https://www.elvocero.com/gobierno/aspira-a-que-haya-m-s-agricultores-bonafide/article_017eaa76-a2b8-11e9-9255-8773684782d5.html

65. Lee TM, Markowitz EM, Howe PD, Ko CY, Leiserowitz AA. Predictors of public climate change awareness and risk perception around the world. Nature Climate Change. 2015;5(11):1014–20.

66. Forero EL, Hernández YT, Zafra CA. Percepción latinoamericana de cambio climático: metodologías, herramientas y estrategias de adaptación en comunidades locales. Una revisión. Revista UDCA Actualidad & Divulgación Científica. 2014 Mar 10;17(1):73–85.

**Formatted:** Font: (Default) Times New Roman, 12 pt, Spanish (Puerto Rico)

**Formatted:** Font: (Default) Times New Roman, 12 pt

**Formatted:** Font: (Default) Times New Roman, 12 pt, Spanish (Puerto Rico)

67. Departamento de Recursos Naturales de Puerto Rico. Estudio de percepción pública sobre el riesgo y la resiliencia al cambio climático [Internet]. San Juan, Puerto Rico: Puerto Rico Department of Natural Resources; 2018 Sep [cited 2020 Jun 13] p. 95. Available from: http://drna.pr.gov/wp-content/uploads/2019/01/Informe-final-Estudio-de-percepcion-publica-sobre-cambio-climatico.pdf
68. Viguera B, Alpizar F, Harvey CA, Martínez-Rodríguez MR, Saborío-Rodríguez M. Percepciones de cambio climático y respuestas adaptativas de caficultores costarricenses de pequeña escala. Agronomía Mesoamericana. 2019 May 1;333–51.
69. Viguera B, Alpizar F, Harvey CA, Martínez-Rodríguez MR, Saborío-Rodríguez M, Contreras L. Climate change perceptions and adaptive responses of small-scale farmers in two Guatemalan landscapes. Agronomía Mesoamericana. 2019;30(2):313'331.
70. Prokopy LS, Arbuckle JG, Barnes AP, Haden VR, Hogan A, Niles MT, et al. Farmers and Climate Change: A Cross-National Comparison of Beliefs and Risk Perceptions in High-Income Countries. Environmental Management. 2015 Aug;56(2):492–504.
71. Chatrchyan AM, Erlebacher RC, Chaopricha NT, Chan J, Tobin D, Allred SB. United States agricultural stakeholder views and decisions on climate change. WIREs: Climate Change. 2017 Oct 9;8(5):n/a-N.PAG.
72. Waldman KB, Attari SZ, Gower DB, Giroux SA, Caylor KK, Evans TP. The salience of climate change in farmer decision-making within smallholder semi-arid agroecosystems. Climatic Change. 2019 Oct 1;156(4):527–43.
73. Debela N, Mohammed C, Bridle K, Corkrey R, McNeil D. Perception of climate change and its impact by smallholders in pastoral/agropastoral systems of Borana, South Ethiopia. SpringerPlus. 2015 May 20;4(1):236.

**Formatted:** Font: (Default) Times New Roman, 12 pt

74. López-Marrero T, Castro-Rivera A. Let's not forget about non-land-falling cyclones: tendencies and impacts in Puerto Rico. Natural Hazards [Internet]. 2019 Aug 6 [cited 2019 Aug 22]; Available from: http://link.springer.com/10.1007/s11069-019-03716-y

75. Demuth JL. Explicating Experience: Development of a Valid Scale of Past Hazard Experience for Tornadoes. Risk Analysis. 2018;38(9):1921–43.

76. Demuth JL, Morss RE, Lazo JK, Trumbo C. The Effects of Past Hurricane Experiences on Evacuation Intentions through Risk Perception and Efficacy Beliefs: A Mediation Analysis. Weather, Climate, and Society. 2016 Oct 1;8(4):327–44.

77. Ranjan P, Church SP, Floress K, Prokopy LS. Synthesizing Conservation Motivations and Barriers: What Have We Learned from Qualitative Studies of Farmers' Behaviors in the United States? Society & Natural Resources. 2019 Nov 2;32(11):1171–99.

78. Código de incentivos de Puerto Rico. P. de la C. 1635, 60 Jul 1, 2019.

79. Campbell, Donovan, Barker D, McGregor D. Dealing with drought: Small farmers and environmental hazards in southern St. Elizabeth, Jamaica. Applied Geography. 2011;31:146–58.

80. Stancioff C, Stojanov R, Kelman I, Němec D, Landa J, Tichy R, et al. Local Perceptions of Climate Change Impacts in St. Kitts (Caribbean Sea) and Malé, Maldives (Indian Ocean). Atmosphere. 2018 Nov 22;9(12):459.

81. Santos-Burgoa C. Acertainment of the Estimated Excess Mortality from Hurricane Maria in Puerto Rico.pdf [Internet]. 2018 [cited 2019 May 20]. Available from: https://publichealth.gwu.edu/sites/default/files/downloads/projects/PRstudy/Acertainment%20of%20the%20Estimated%20Excess%20Mortality%20from%20Hurricane%20Maria%20in%20Puerto%20Rico.pdf

**Formatted:** Font: (Default) Times New Roman, 12 pt, Spanish (Puerto Rico)

**Formatted:** Font: (Default) Times New Roman, 12 pt

82. Wilson RS, Herziger A, Hamilton M, Brooks JS. From incremental to transformative adaptation in individual responses to climate-exacerbated hazards. Nature Climate Change. 2020 Feb 10;1–9.

83. Graham B. Profile of the Small-Scale Farming in the Caribbean. Food and Agriculture Organization; 2012 p. 62.

84. Ribot J. Cause and response: vulnerability and climate in the Anthropocene. The Journal of Peasant Studies. 2014 Sep 3;41(5):667–705.

# Supporting information

**S1 Table. Exploratory factor analysis results for pilot study's assessment of the psychological distance of climate change scale.** Factor loadings are shown, as well as the Cronbach's alpha for statements altogether.

**S2 Table. Structural equation model structural results for our hypothesized model.** Standardized coefficients (β), bootstrap standard error (SE), and significance level (*p*) are included.

**S~~2~~3 Table. Structural equation model structural results for indirect effects of our hypothesized model.** Standardized coefficients (β), bootstrap standard error (SE), and significance level (*p*) are included.

**S~~3~~4 Table. Structural equation model structural results for total effects of our hypothesized model.** Standardized coefficients (β), bootstrap standard error (SE), and significance level (p) are included.

**Response to Reviewers**

**Dear Editor and Reviewers,**

**We thank you for taking the time and energy to review our manuscript. Your thoughtful comments and recommendations have been valuable in improving our manuscript's clarity and rigor. Below you can find our responses to specific reviewer comments in bold. At the end, we also include the updated Competing Interests section, as requested in the PLOS ONE Decision email.**

PONE-D-20-19200

Reviewer #1: This article reports the results of a survey of Puerto Rican farmers aimed at measuring their motivation to adapt to climate-exacerbated hazards (hurricanes) as a function of psychological distance, vulnerability and capacity. They also examine the effect of prior and recent (Maria) experience on distance through a structural equation model. While I found the study to be timely and an interesting contrast to the majority of the literature in this space (e.g., subsistence farmers in developing contexts, big scale commodity farms in developed contexts, etc), I have some issues with the theoretical framing and the conclusions that are drawn given the variability in the data and the way that variables were operationalized. I think perhaps the most interesting story in this data is this idea that psychological distance may not be a critical lens for understanding motivation in some contexts. The dominant narrative is to "bring the risk closer", but there is some evidence that this is not always better, and this data in particular demonstrates that the risk is both near and far, but that it is the perceived capacity to adapt that best explains motivation. I do agree with the author's conclusions that moving beyond individual or psychological variables may be necessary to really understand motivation and adaptation in contexts like this (and others). This is an argument made in a recent review in Nature Climate Change by Wilson et al.

While the analysis seems appropriate and rigorous, I do worry about the fit of the data for a parametric analysis - it is not normally distributed. And given that reality, along with some issues in measurement, it causes me to question some of the conclusions. I do not see that the data has been made available, but perhaps I missed this. And while the writing is fairly strong, there are some grammatical issues that need addressed. My specific line by line comments are in the attached PDF, but I would make these major suggestions.

1) Be more clear in your introduction about your theoretical framework. The intro focuses largely on psychological distance, and to some degree the role of experience, in explaining motivation to adapt. But then other variables are brought into play (vulnerability, capacity). Why are these included? Why are these the likely theoretical drivers of one's motivation to adapt? Why would we expect distance to help explain vulnerability and capacity? What have other studies found about this suite of variables? Again, you focus on experience and distance, but this must be a reason you included these other variables in your model. And given they turn out to be the most important variables, you need a better review up front. While this may be the first study of farmers in small island developing contexts, there are likely other studies that look at actual

experience with a hazard and distance...this was not clear from your review. I think you need to think carefully about what background literature is necessary to situate your findings - and it seems that you need to speak to what is known about experience and distance broadly (and in particular actual experience), and then you need to speak to the role of distance, vulnerability and capacity on motivation. I would start by broadly summarizing what might be known in the adaptation literature at large, but then narrowing in on what is known specifically in the context of agriculture (where that exists).

**Response: We have restructured our introduction, and have broadened specific lines, as well as in the literature review section, to be more specific about the extent to which experience and psychological distance relate, and about why we included the variables you mentioned. We focused on literature that used the psychological distance of climate change (PDCC) as its framework to understand behavior or literature that evaluated PDCC after an event. The main argument of such literature is that reducing psychological distance will prompt mitigation or adaptation behaviors. Here, instead of evaluating adoption of practices, for example, we assessed the extent to which PDCC is linked to reported levels of perceived capacity, vulnerability, and motivation to adapt, which are variables important in adaptive capacity research from a social and psychological perspective. We argued that in order to understand adaptation, which reflects adaptive capacity, we must understand social and cognitive drivers. Thus, we used PDCC to understand how Puerto Rican farmers perceive climate change after the brunt event of Hurricane Maria, how that then that was linked to sociodemographic and reported experience variables, and how PDCC was linked to other adaptation perceptions. Hence, our main goal in this paper was to assess variables that to the best of our understanding have not been studied in Puerto Rico. In the reviewed discussion we underscored that future studies should inquire more deeply about what explains farmers' perceived capacity, vulnerability, and motivation to adapt from different angles, given that those variables have been shown to be pivotal in adaptation research. It is important to reiterate that this paper is about understanding farmers' PDCC after a category 4 hurricane.**

**In terms of your comments regarding the discussion, we appreciate that you highlighted Wilson et al. (2020). We were not aware of that review paper, and we think that their argument aligns we our discussion. We included it in our discussion, thank you.**

**Regarding your comments on the structural equation model (SEM), we have used bootstrapping (n=1,000), a documented method to control for a number of issues in structural equation models, including non-normal data. We have clarified in the methods section that using Bootstrap in SEM is recommended for non-normal data. We also intend to share the non-identified data in a repository after the acceptance of the paper.**

2) Similarly, you kind of gloss over some of the variables in your methods in terms of how they were operationalized. I also have some other issues with your operationalization and then the conclusions you draw. To what extent might experience have played a bigger role if it was a more nuanced measure? See excellent work by Julie Demuth on measuring experience with natural hazards, her work is largely on tornados, but might be worth perusing. I am not

convinced that experience does not matter, it just seems that your metric of experience did not matter. One could argue that accumulated experience with hurricanes over time has moved climate change from a distant to a distant and near threat, but that your study does not capture this dynamic process. Greater clarification on your measures is necessary in general - why these items? Are they validated scales? Have they been used elsewhere?

**Response: Thank you for your comments on experience and for bringing Demuth's work to our attention. We clarified in the paper about our use of the experience variables, both reported hurricane damages and reported experience with similar events. The word "reported" was added to be specific about the variables. Furthermore, in the reviewed discussion, we expanded on "experience" to capture your comments and recommendations. It was also included in the limitations section. Nevertheless, it is important to note that in in the literature review section (Research on the psychological distance of climate change), we highlighted the work by Zanocco et al. (2018) to inform why used reported damages as one measure of experience. Other PDCC studies have used the single reported experience scale, and other studies have been done in an area after an extreme event without asking about that experience. We clarified in the methods about the studies that informed our survey instrument, and how variables have been used in previous studies. Nevertheless, as Demuth shows, the use of different experience measures is a current barrier in existing studies. We recommended the further investigation of what it means to experience an extreme event in Puerto Rico, and we also added historical information on landing and non-landing hurricanes and tropical storms in Puerto Rico. It is important to note that our questions were framed to capture participants' opinions and perceptions around Hurricane Maria. Puerto Rico was still recovering when the survey was carried out. We also included a section to explain the assessment of social and cognitive variables, and we included more information on how the PDCC scale was built. Finally, we included supplementary material on the pilot's PDCC analysis, which in demonstrating reliable validity, was another factor in our design.**

3) Finally, I think the discussion could better situate your findings in the context of other research on behavioral/psychological factors and climate adaptation, as well as other work on farmers and climate beliefs. You mentioned the van Valkengoed and Steg meta-analysis - how do your results match or not with their findings? You mention in your intro that experience was not significant int heir analysis, but what about your other variables? Vulnerability? Capacity? To what extent have these variables been studied in farmer specific studies? Also, there were several studies out of Purdue on Midwestern US farmer climate beliefs. It would be helpful to situate Puerto Rican farmers within this broader context of global agriculture - how are they similar? How are they different? It is not surprising to me that they are taking this threat more seriously, but that the biggest issue might be capacity to act. A more nuanced discussion of this decision process for this group vs. other farmer groups studied would be useful. Behavior change is often a function of both motivation and ability - Puerto Rican farmers seem to have the motivation (concern, perceived personal risk, etc) but not the ability. While other US farmers (like in big Midwest commodity ag) may lack that initial motivation or concern.

**Response: Thank you for your comments and recommendations. We have expanded our discussion. We think that understanding the decision-making process around adaptation**

**should be further looked into in future studies. We explain in our discussion why this is important, and how our results support those recommendations.**

Reviewer #2: This paper provided a comprehensive understanding about the relationships among Puerto Rican farmers' psychological distance of climate change, their experience of the category 4 Hurricane Maria and their perceptions of climate change. Then the paper explored further the association of these variables to farmers' motivation to climate change adaptation.

It is worth understanding the roles of extreme weather events experience and perceived psychological distance in farmers' decision making around climate change adaptation. Besides, this paper supplement a research in an island-archipelago setting to a more comprehensive view of perceptions and adaptation of climate change, which is valuable for future researches and practices.

However, there are some issues (such as the definition of psychological distance and its analysis in the models) that need to be better considered according to the survey and objective design. Also, the results need to be better interpreted for a more concrete conclusion. Below are my comments:

1. Main: about the psychological distance

In the Theoretical framework section (P6), it gave the explanation of psychological distance with the four components. Then according to the Objective, research questions, and hypotheses section (P11), it's mainly on how the degree of psychological distance (how far or how close) linked to the extreme experience or damage, and how it associates with the cognitive perceptions and motivation of adaptation. However, in Methodology on Assessing the psychological distance (P13, line 285) and the survey (Table 1), it redefined "psychologically far" and "psychologically close" for each item. There are some questions about it:

1）Line 285, "Strongly agree" and "agree" answers for dimensions' statements were combined as "psychologically far" — How about the reversed items? Usually, negatively worded statements were used as reversed items for controlling the response bias with reverse scoring. But here, it seems only the meaning of local or global were used as the reversed items, instead of negative statements. Need to be careful about this for the further analysis.

**Response: Thank you for the opportunity to further clarify our method, and we apologize for the initial confusion. The psychological distance of climate change (PDCC) scale was developed from eight, separate 5-point Likert scale items. Your reference above is related to our presentation of results in figure 4. Statements were not combined for statistical analysis. Given that the scale measured ascending PDCC (e.g. the higher the number, the more distant the farmer perceives climate change), we presented the results as "psychologically far" for the combination of "agree" and "strongly agree" responses, and "psychologically close" for "disagree" and "strongly disagree responses". We have decided to eliminate that way of showing results to prevent confusion, and we clarified how we analyzed results in the Methods section.**

2) According to such redefinition and analysis, it has regarded close and far as a one-dimensional variable (either close or far) instead of two-dimensional (how close and how far in those four components at the same time). Nevertheless, the design of the survey items divided close and far into two items, which made it a bit confusing. The results also showed the farmers could perceive both close and far. So maybe there is also a need to consider the close or far into two dimensions for analysis.
It could change to the exploration of the relationship between how close they perceived (the degree of close) in four components and their experience or beliefs. Further, how far they perceived (the degree of far) in those components need to be separated for the two items (General public and Global agriculture).

**Response: All the items were evaluated as 5-point Likert scales. To ideally control for "straight-lining" in a survey, we intentionally had some questions worded oppositely so that we could conduct data quality checks. Some questions were reversed so that responses were shown in ascending levels of PDCC. In terms of interpretation, we interpreted "agree" and "strongly agree" to be "psychologically far", and "agree" and "strongly agree" to be psychologically close. We have clarified in the manuscript how we analyzed and interpret these items and the PDCC scale.**

3) Based on above, if focusing on the degree of psychological distance perceived, maybe keeping 5-point Likert scale coding for analyses would be suitable than 3-points.

**Response: We clarified in the manuscript that they were interpreted and statistically analyzed as 5-point Likert scales.**

4) on Line 284, it showed the Reliability Analysis with Cronbach's alpha value for the all items in psychological distance scale, could you also provide the Cronbach's alpha value for each component (more than one item) to see the internal consistency?

**Response: We included in the Methods section more information on how we created the scale from the pilot study's data. We report reliability analysis with Cronbach's alpha and factor analysis results. Alpha is recommended for scales with three or more items. We expanded in our Methods section about what informed our variable use.**

5) Besides Reliability Analysis, could you also provide the results of validity analysis, such as factor analysis? This could help to see whether the analysis of psychological distance scale could provide the support for the two dimensions on close and far, or four dimension on theoretical components. Then according to the factor analysis, it could be better consider whether still regard psychological distance as one variable in the models.

Based on the above, I suggest that the authors better consider and interpret the psychological distance and related results. Would they be confident of their final results that "the experiences were not linked to their psychological distance of climate change, and these did not relate to their motivation to adapt", when using the current ways on the definition and analyses.

**Response: Thank you for your comments and recommendations. We better explained what we meant by "experience" in the paper, and we included historical records of landing and non-landing hurricanes in Puerto Rico to our discussion. We also performed a reliability analysis and factor analysis with the pilot data, which informed our PDCC analysis. We expanded on Methods, and included the new supplementary materials.**

2. Main: about the experience (with events and damages)

1）Line 184-185, it seems the direct experience in this paper means the reported degree of damage. But how does one distinguish the reported experience from reported damage level? People could perceive the events and reported the experience through the damage experience. So, it is hard to say that those researches only used data on reported experience that were not related to direct experience, such as Spence's work cited in this paper.

**Response: In our revision, we clarified on what we meant by experience, how we assessed it, and what can be done in the future to further understand what it means to experience an extreme weather event in Puerto Rico. We had two variables, one that asked about reported experience with similar events in the past 10 years, and one that asked about farm-level damaged caused by Maria. It is important to note that Hurricane Maria affected all of Puerto Rico. It has been the most devastating storm to hit Puerto Rico in 89 years. People were still recovering when the study was carried out. We also included historical records in the discussion to further expand on experience and PDCC.**

2）Line 201, it said the paper would assess the extent to which the experience with Maria (both reported experience with other extreme events…). And in Objective section, the Objective 2 and H1 all mentioned the experience with Maria. While based on the only one item for the reported experience in table 1, is the statement framing mainly for the experience with other extreme weather events except Hurricane Maria or for the experience with Hurricane Maria?

**Response: Thank you, we clarified the variables in the objectives, methods, and overall discussion.**

3）If the reported experience is focusing on the experience with other extreme weather events except Hurricane Maria, could you provide more introduction on the extreme weather events happened in this area before Maria?

**Response: We included data on historical records regarding hurricanes in the discussion.**

4）Besides, it is needed to clarify the interpretation framing with the experience related results, if the experience from the survey is not experiencing Maria, e.g. in Abstract "Reported experience and direct damages by the hurricane were not linked to their psychological distance".

**Response: We clarified how we understand and assessed experience.**

5）Line 180, it mentioned the extreme events may have become "more normal" and this view was also mentioned in discussion to explain the weak role of experience in the model. It also

could be better if you could provide more background information on the historical extreme events the people there may experienced.

**Response: We have included these suggestions.**

Other comments:

3. line 267, could you clarify the ways that the surveys were randomly administered to farmers in Data gathering section?

**Response: We expanded on this in the methods section.**

4. Adding the Cronbach's alpha value into the table 1 if it is available.

**Response: Cronbach's alpha for the main study's scale is included in the text of the Assessing the psychological distance of climate change section in Methods.**

5. Adding the citation into table 1 if there are some items referring to other researches.

**Response: Thank you for the recommendation. We added the citations in text.**

6. Could combine table 2 and table 3 into one comprehensive table, then could consider to put it into supplementary sheet if there is more table/figures than requested.

**Response: We have decided to keep the tables separate. Thank you for the recommendation.**

Here is our update Competing Interests section:

> "I have read the journal's policy and the authors of this manuscript have the following competing interests: MTN is a member of the board of directors of The Public Library of Science (PLOS). This role has in no way influenced the outcome or development of this work or the peer review process, nor does it alter our adherence to PLOS ONE policies on sharing data and materials. This does not alter our adherence to PLOS ONE policies on sharing data and materials."
