# Supplementary material for: Awareness of climate change's impacts and motivation to adapt are not enough to drive action: A look of Puerto Rican farmers after Hurricane Maria
Source: PLoS One. 2021 Jan 27;16(1):e0244512. doi: 10.1371/journal.pone.0244512 (PMC7840010; doi:10.1371/journal.pone.0244512)
Supplement: Attachment — Submitted filename: Response to Reviewer.docx [file pone.0244512.s009.docx]

**Response to Reviewers**

**Dear Editor and Reviewers,**

**We thank you again for taking time and energy in these challenging times to review our manuscript. We have appreciated your comments, which have elevated the manuscript’s clarity and rigor. We have included below the responses to Reviewer 1 comments.**

PONE-D-20-19200R1

Reviewer #1: Unfortunately, I do not feel that my prior comments were sufficiently addressed. This paper still suffers from a lack of proper set-up in the introduction to support the analyses and related discussion. The paper is framed as a study of experience, psychological distance and adaptation. However, the study then diverges to include other key beliefs that may influence one's motivation to adapt. Yet, the inclusion of these beliefs in the SEM, and the theoretical framework guiding their inclusion (and placement in the proposed structure) is not addressed. For example, why would we expect distance to influence vulnerability and self-capacity? I can intuit the former, but not the latter. Theoretically, the perceived distance of climate change and perceived vulnerability would work in parallel with self-capacity to influence adaptation decisions. e.g., One can be concerned about local impacts and be motivated to act, but not able to act due to limited capacity. I don't think the paper properly deals with these constructs - the issues begin the measurement of these ideas with single items, but extend to the proper theoretical hypotheses for their relationships. The authors in fact make this argument in the discussion that perhaps the bigger issue in Puerto Rico are the structural barriers to action, not a lack of appropriate concern about climate change, but then why do the respondents report such high self capacity? If you are intending to differentiate between motivation and intention, then be more clear about this. You didn't measure intention to adapt or actual adaptation, you measure "motivation" - but I think most of the behavioral literature would argue that motivation can be high, but ability low, and then there is a failure to act.

I would suggest a few paths forward for the authors.

1) Rethink your conceptual model. If the strength of your study is the experience, distance and motivation piece, then stick to that. Your measures of vulnerability and self-capacity are not strong (single item) and their position in the proposed model is not well defined or defended. In fact, their position (self-capacity in particular) runs contrary to much of your conclusion around the importance of structural barriers. If structural barriers are so key? Then why are your respondents reporting such high capacity? If you focus on distance and experience, which your intro sets up sufficiently, then perhaps split your distance items in your SEM to look at separate paths through each dimension. Or perhaps through the "near" vs. "far" dimensions. If you measure is capturing a continuum from near to far and respondents score strongly on both, then combining those items will wash out any potential effect. Separating them out would allow you to test if a particular perspective is driving their motivation - you may find that experience does drive the "near" dimension, and that the near dimension does drive motivation. The paper almost reads like you intended this to be a study on distance and experience, but then there were no effects so you threw in a few other relevant items to have a story to tell. I don't disagree with your story and conclusions in theory, but I don't think your data support that story.

**Response: Thank you for your recommendations. We agree that single-item measurements are a limitation, which we include in our limitations section. To address your concerns, we have expanded sections of the paper, especially those areas that focused on perceived self-capacity to better set up the context and framing for their inclusion. Your comments were helpful to improve consistency regarding concepts used, especially in motivating us to better clarify on the differences between “adaptive capacity” and “climate change adaptation”. It is important to note that this paper was based upon Spence et al. (2011), where they look at United Kingdom residents’ climate change mitigation support in light of their experience with floods, in function of experience driving concern for climate change, uncertainty, perceived capacity, and perceived vulnerability. Their study has been the baseline for studies using the psychological distance of climate change as a framework. Our model is built upon their findings, including the similar constructs. We hope that the additions of our clarifications, including the focus of Spence as a guiding framework, helps further clarify our work. However, in reviewing your feedback, we also considered our model further, specifically as it related to your comment about why if people had so much perceived capacity is there a problem? Ultimately, our model was only measuring motivation to adopt, not actual adoption, and literature suggests that such constructs are critically different. Thus, in this revision, we have added a new dependent variable: actual adoption of agricultural practices and management strategies after Maria. The addition of this variable helps complete a more nuanced understanding, and connection between capacity, motivation and actual behavioral change. As a result of adding this construct, we believe that a more complete story is told with these results- while perceived capacity is high, and motivation to adapt is high, actual adoption of adaptation practices is not related to motivation to adapt. This clearly shows the gap and barriers between motivation and perceived capacity and actual behaviors, which are hindered potentially by other factors, including structural factors, that we mention in our discussion.**

2) Think carefully about your language and terminology throughout the paper - you talk about motivation to adapt, adaptive capacity, self capacity, cognitive beliefs, context, structure, adaptation beliefs, etc. etc. It isn't always clear what you are talking about and if you are using terms interchangeably (e.g., motivation to adapt vs. adaptive capacity) or if you are using certain phrases to refer to sets of your constructs (e.g., adaptation beliefs for everything vs. cognitive beliefs for three of your measures). Choose one set of terms for the idea in the paper and define them clearly at first mention, then use them consistently throughout. And be sure that your terminology reflects best practice and what will be most intuitive to those familiar with this literature.

**Response: Thank you for providing this additional feedback, and your pdf comments on the concepts used. We have done a careful review of our terms and reviewed them in our revisions. We better defined the concepts, and made revisions for them to be constant in the manuscript.**

My specific line by line comments are attached in the PDF.
